# Antinociceptive activity of *Laportea* species mediated by anti-inflammatory and antioxidant mechanisms: a systematic review and meta-analysis of in vivo animal studies

**DOI:** 10.1186/s12906-026-05262-0

**Published:** 2026-02-03

**Authors:** Nurasi Lidya E Marpaung, Ronny Lesmana, Enny Rohmawaty, Melisa Intan Barliana

**Affiliations:** 1 Doctoral Program of Medical Sciences, Faculty of Medicine, Universitas Padjadjaran, Jl. Raya Bandung-Sumedang, KM.21, Hegarmanah, Jatinangor, Sumedang, West Java 45363 Indonesia; 2 Department of Physiology, Faculty of Medicine, Universitas Papua, Manokwari, West Papua 98444 Indonesia; 3 Physiology Division, Department of Biomedical Sciences, Faculty of Medicine, Universitas Padjadjaran, Jatinangor, Sumedang, West Java 45363 Indonesia; 4 Pharmacology Division, Department of Biomedical Sciences, Faculty of Medicine, Universitas Padjadjaran, Jatinangor, Sumedang, West Java 45363 Indonesia; 5 Department of Biological Pharmacy, Faculty of Pharmacy, Universitas Padjadjaran, Jatinangor, Sumedang, West Java 45363 Indonesia; 6 Center of Excellence for Pharmaceutical Care Innovation, Universitas Padjadjaran, Jatinangor, Sumedang, West Java 45363 Indonesia

**Keywords:** Analgesic, Cytokines, Inflammation, Laportea, Oxidative stress, Pain, Urticaceae

## Abstract

**Background:**

*Laportea* species (a genus of the nettle family, *Urticaceae)* have a history of traditional use for relieving pain. Recent preclinical evidence suggests their antinociceptive activity is mediated through modulation of inflammation and oxidative stress, yet no integrated synthesis of these mechanisms has been reported. We performed a systematic review and meta-analysis of in vivo animal studies to address this gap, by assessing the pain-relieving efficacy of *Laportea* species, identifying key biochemical markers of their action, and evaluating the impact of methodological considerations on pharmacological results.

**Methods:**

PubMed, Scopus, ScienceDirect, EBSCO, and Google Scholar were systematically searched for in vivo animal experiments, examining *Laportea* species extracts and elucidate associated anti-inflammatory and antioxidant mechanisms. Data extracted on nociceptive behavior, cytokine profiles, oxidative stress markers, and antioxidant enzyme activity. Effect sizes for treatment versus control were calculated as SMDs with 95% CI, and subgroup analyses examined the impact of dosage, extraction type, administration route, species variation, and treatment duration.

**Results:**

There were sixteen studies included. Extracts of *Laportea* species significantly suppressed cytokines (IL-2, IL-1β, IFN-γ, TNF-α, IL-6) and reduced oxidative damage biomarkers (malondialdehyde, protein carbonylation, nitric oxide), enhancing anti inflammatory cytokines (IL-10 and TGF-β) and endogenous antioxidant (peroxidase, glutathione, glutathione peroxidase, superoxide dismutase, glutathione transferase, catalase, and glutathione reductase). These molecular effects correlated with reduced pain behaviors, including decreased writhing and increased pain reaction time. The strength of these effects varied depending on extraction methods, duration of treatment, administration route, tissue specificity, animal species model used, and *Laportea* species varieties.

**Conclusion:**

The antinociceptive activity of *Laportea* species is closely related to their anti-inflammatory and antioxidant properties, suggesting a multi-targeted mechanism of action. These findings support the potential of *Laportea* species extracts as phytotherapeutic agents for managing pain and related inflammatory conditions. Further translational studies are needed to clarify clinical relevance and optimize bioactive formulations.

**Supplementary Information:**

The online version contains supplementary material available at 10.1186/s12906-026-05262-0.

## Background

The management of pain, particularly chronic and inflammatory pain, remains a formidable global public health challenge, evidenced by its widespread prevalence across diverse populations [1]. Traditional pharmacological strategies, dominated by opioid analgesics and non-steroidal anti-inflammatory drugs (NSAIDs), are increasingly limited by significant constraints. Opioids present severe risks concerning tolerance development, dependency, and addiction, contributing to a worldwide crisis. Simultaneously, the long-term clinical utility of NSAIDs are associated with substantial side effect profiles, including gastrointestinal and cardiovascular toxicities [2, 3].

A deeper understanding of the mechanisms underlying pain reveals a complex process involving immunological and oxidative pathways. Inflammation, a protective tissue response, triggers nociceptor sensitization, and this process is critically compounded by oxidative stress (OS). Oxidative stress occurs when the production of reactive oxygen species (ROS) overwhelms endogenous antioxidant capacity, directly sensitizing nociceptive neurons and reinforcing the transition to chronic pain. Therefore, the most promising therapeutic strategies are those that exert dual functionality, simultaneously mitigating pro-inflammatory mediators (e.g., NF-kB or COX-2) while enhancing endogenous antioxidant defenses (e.g., via Nrf2 activation) [4, 5].

This mechanistic hypothesis provides a robust framework for evaluating multi-targeted natural compounds.The medicinal potential of natural products has been extensively acknowledged, especially because of their affordability, availability, and proven safety profiles, and inherent capacity for multi-target engagement [6–8]. Within this domain, the genus *Laportea* (family *Urticaceae*), which comprises approximately 28 species found primarily across subtropical and tropical regions of Asia, Africa, and South America, possesses a significant history of traditional use. Species within the *Laportea* genus have been employed ethnopharmacologically for generation to manage pain, inflammation, and related conditions, utilizing various plant parts including the leaves, roots and stems [9, 10].

*L.bulbifera* is utilized by ethnic minorities (China) to treat conditions such as arthritis and pain, while tribes in Tripura, India, employ *L. interrupta* to alleviate complications like chest pain and general inflammation. These traditional preparations are typically administered orally as infusions or decoctions, or topically as poultices. Phytochemical screening of various *Laportea* species has confirmed a rich profile of secondary metabolites, including flavonoids, phenols, alkaloids, and terpenoids, which underpin its pharmacological potential. Specifically, the prominent presence of phenols and flavonoids directly supports the dual-mechanism hypothesis, as these compounds are well-known for their potent antioxidant capacity, which neutralizing free radicals and protecting against oxidative damage, and their effective anti-inflammatory activity [11].

Preclinical studies investigating *Laportea* species have demonstrated promising antinociceptive, anti-inflammatory, and antioxidant effects in vivo animal models [12–15]. However, these findings remain fragmented and suffer from considerable methodological heterogeneity, which is unavoidable in natural product research. This systematic review and meta-analysis fill this critical gap by providing a statistically robust, quantitative synthesis of the in vivo animal studies evidence. The application of advanced biostatistical techniques is essential for translating traditional knowledge into evidence-based therapeutic applications. This quantitative rigor is necessary not only to confirm the overall antinociceptive efficacy but also to identify the optimal experimental parameters that correlate with the strongest therapeutic outcomes.

Therefore, the primary objective of this systematic review and meta-analysis is to quantitatively evaluate the overall antinociceptive efficacy of *Laportea* species extracts and isolated compounds in in vivo animal studies. Secondary objectives include: firstly, verifying and quantifying the role of anti-inflammatory and antioxidant mechanisms by correlating behavioral antinociception with measurable biochemical biomarkers. Secondly, to identify optimal parameters by conducting subgroup analyses based on methodological variables such as *Laportea* species, extraction solvent, dose, animal species and model, route of administration, treatment duration. This comprehensive quantitative assessment provides a crucial foundation for advancing *Laportea* from traditional use toward evidence-based clinical consideration.

## Materials and methods

This systematic review and meta-analysis followed the PRISMA 2020 guidelines [16]. Registration of the study protocol was made on PROSPERO database (CRD42024497367).

### Literature search strategies and selection criteria

Four researchers independently searched five online databases: PubMed, Scopus, ScienceDirect, EBSCO, and Google Scholar, covered the period from the inception of the database until February 2024. Supplementary searches were performed to identify grey literature, including academic theses, dissertations, and conference proceedings, alongside non-indexed primary studies published in the Indonesian language, primarily utilizing advanced features within Google Scholar.

The search strategy was designed to maximize retrieval sensitivity by combining controlled vocabulary (e.g., MeSH terms in PubMed) with free-text terms. This dual approach is vital for preclinical SRMAs, ensuring comprehensive identification of in vivo animal studies concerning the analgesic, anti-inflammatory, or antioxidant effects of *Laportea* species in animal models. The core search logic employed was: (Laportea) AND (anti-inflammatory OR anti-inflammation OR antioxidant OR analgesic OR anti-nociceptive OR in-vivo OR rat OR mice). The full search strings, which were necessarily adapted for the specific field codes and controlled vocabulary of each of the five databases to ensure replicability, are provided in supplementary (See Additional File 1).

Articles were managed using Mendeley software, with duplicates removed prior to screening. Two reviewers (N.L.E.M. and R.L.) independently assessed the titles and abstracts; differences were resolved through agreement or by the intervention of an independent adjudicator.

### Inclusion and exclusion criteria

Studies was chosen according to the PICOS structures: (a) population: animal models subjected to pain, inflammation, or oxidative stress; (b) intervention: *Laportea* extract administered orally, topically or other routes, (c) comparison: untreated or vehicle control group; (d) outcomes: quantitative markers of inflammation, oxidative stress, or antinociceptive; (e) study design: in vivo animal experimental trials.

The inclusion criteria were: (1) experimental studies using *Laportea* species compared to a control group; (2) studies within the scope of health and medicine relevant to humans, from the inception of the database until February 2024; (3) in vivo animal studies; (4) original studies articles including grey literatures published in English or Indonesian. Exclusion criteria were: (1) case reports, reviews; clinical trials, in vitro; (2) multi-herbal formulations including *Laportea* species.; (3) insufficient or non-extractable data on relevant outcomes.

### Data extraction

Studies meeting the inclusion criteria were reviewed, and the following information was extracted: bibliographic details and study design, *Laportea* species, extract type, sample size, animal species, intervention, dosage, and primary outcomes. Data from the final time point were extracted when outcomes were presented at multiple intervals. In instances where results were presented solely in graphical format, data were extracted utilizing WebPlotDigitizer 4.8 (https://apps.automeris.io/wpd4/).

### Study quality assessment

Study quality assessment was performed independently by three investigators (NM, RL, ER) through the SYRCLES Risk of Bias (RoB). This tool is adapted from the Cochrane RoB instrument for clinical studies and incorporates the essential methodological criteria recommended by the Collaborative Approach to Meta-Analysis and Review of Animal Data from Experimental Studies (CAMARADES) reporting standards [17].

The bias assessment included the following items: selection, performance, detection, attrition, reporting and other sources of bias. Selection bias examines whether groups were comparable at the start of the study and whether the process of assigning animals to groups was truly random and concealed. This includes assessing sequence generation (the method used to randomize groups), baseline characteristics (if groups were comparable in terms of weight, age, etc.), and allocation concealment (whether investigators were prevented from knowing the next group assignment beforehand) [18].

Performance bias addresses potential differences in care or environmental conditions during the experiment that could influence outcomes. This is evaluated by random housing (whether animals were handled or housed similarly) and blinding of caregivers/investigators (if the personnel administering the intervention were unaware of the group assignment). Detection bias scrutinizes whether the measurement of outcomes was fair and unbiased. It assesses random outcome assessment (whether the selection of animals or samples for analysis was random) and blinding of outcome assessors (if the personnel measuring the results, such as reading assays or counting responses, were unaware of the treatment groups) [18].

Attrition bias checks for incomplete outcome data, ensuring that any loss of animals or data (e.g., dropouts) was minimal, balanced, and appropriately accounted for across all groups. Reporting bias assesses selective outcome reporting by checking whether all outcomes that were planned or measured in the methods section of the original study were fully and transparently reported in the results. Other bias allows reviewers to flag any other specific design flaws, ethical issues, or external factors that could potentially introduce a high risk of bias into the study findings.

Each criterion was classified as low risk of bias (+), high risk of bias (-), or unclear risk (?). Low risk indicates that the study adequately described and performed measures to minimize bias for that specific item. High risk indicates that a study’s methodology was flawed or inappropriately executed, suggesting a high potential for systematic error in the results. Unclear risk indicates that insufficient detail or information was provided in the primary study’s manuscript to definitively judge the risk of bias as either low or high (a critical reporting gap, often marked by “yellow” in graphical summaries) [18]. Disagreements were resolved by a fourth investigator (MB).

### Statistical analysis

R software (version 4.2.2, with the packages metafor and meta) was used to conduct the meta-analysis. Standardized mean differences (SMD) with 95% confidence intervals (CIs) were used to express the treatment effects. Statistical significance was defined as a p-value (less than 0.05). The effect size was determined using the outcome of interest and the means and SDs for each study group. Forest plots were utilized to visually depict effect sizes and 95% confidence intervals. Effect sizes were categorized as follows: large effect (> 0.8), moderate effect (0.5–0.2), and small effect (< 0.2) [19].

The I² statistic was employed to evaluate heterogeneity, with values exceeding 50% signifying substantial heterogeneity, prompting the use of a random-effects model. A fixed-effects model was utilized in the absence of substantial heterogeneity [20]. Subgroup analyses were performed to explore potential sources of heterogeneity, and sufficient data were provided. Variables included: oral dosage (100 mg/kgBW; 101–500 mg/kgBW; >500 mg/kg BW), topical dosage (< 0.6%; 0.6–1.5%; >1.5%), animal species (rat, mice), extraction solvent (methanol, ethanol, aquous, crude), topical form (cream, patch), duration of intervention (1–3 days; 4–7 days, more than 7 days), *Laportea* species (*L.aestuans*,* L.bulbifera*,* L.ovalifolia*,* L.interupea*,* L.decumana*), methodological (pain / inflammatory / oxidative stress animal model), organ / tissue examined (paw, gastric, liver, heart, lung, kidney, serum, brain). Egger’s test and funnel plots were used to evaluate publication bias; a p-value of less than 0.05 denotes statistical significance [21].

## Results

### Study selection

**Fig. 1:**
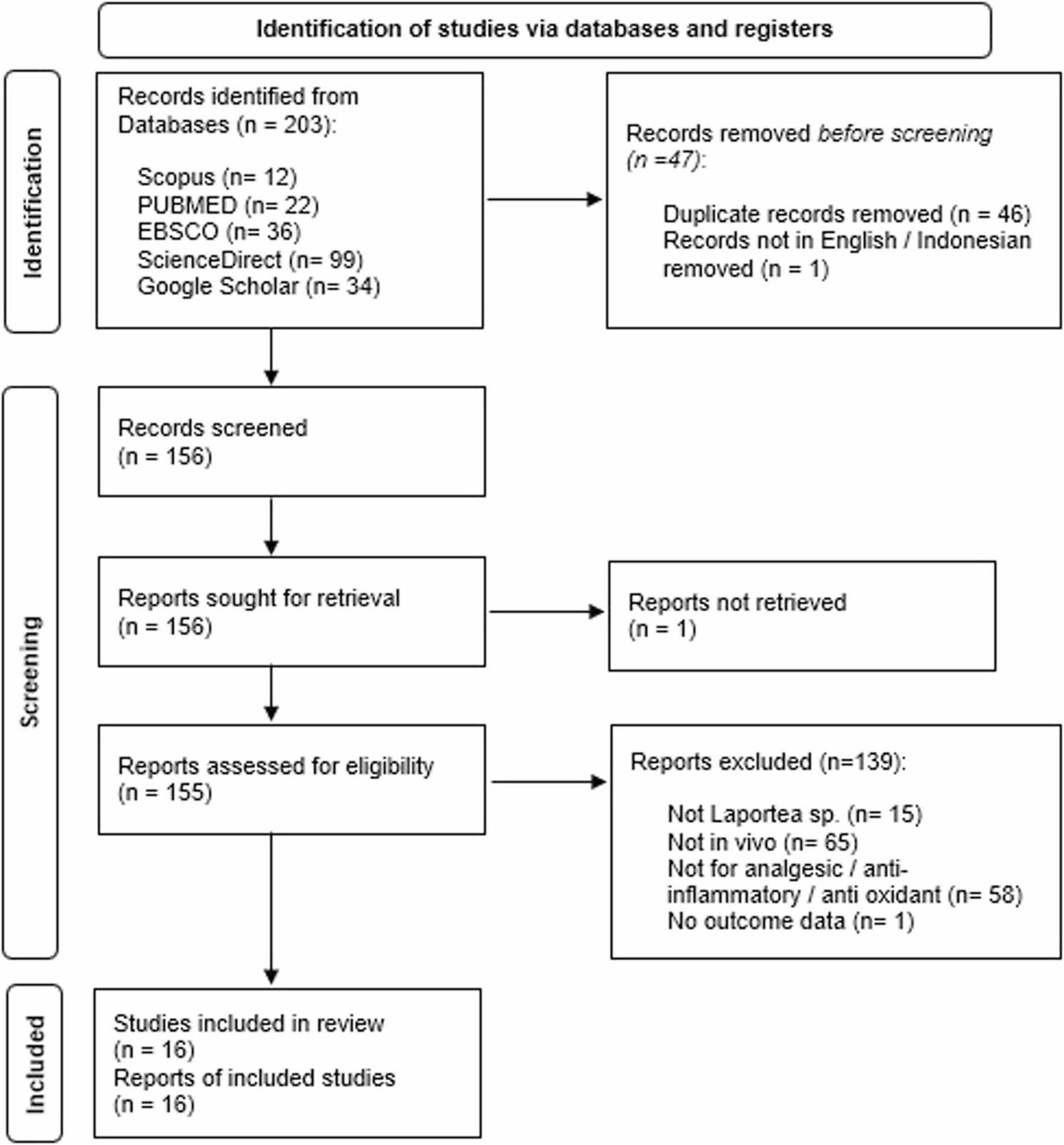
PRISMA flowchart (PRISMA figure adapted from PRISMA 2020)

Figure 1 illustrates the procedure used to choose the articles. The search process identified 12 in SCOPUS, 99 in ScienceDirect, 23 in PubMed, 34 in EBSCO, 35 in Google Scholar, yielding a total of 203 articles. The remaining 158 titles and abstracts were reviewed after 45 duplicate papers were eliminated. The meta-analysis finally contained 16 articles after excluding those that were not published in English or Indonesian or those unrelated to the focus of this study.

### Characteristics of included studies

Table 1 provides a concise summary of the characteristics of the studies included in this systematic review and meta-analysis. The investigations were conducted across diverse geographical regions, highlighting the global research interest in the *Laportea* genus, with studies originating from Nigeria [22–27], Indonesia [13, 28–30], Cameroon [15, 31], Bangladesh [32], Ghana [33], and China [34]. The evaluation focused specifically on five *Laportea* species: *L.aestuans*, *L.bulbifera*, *L.ovalifolia*, *L.interupea*, and *L.decumana*. The majority of the research utilized the leaf parts of the plant (15 studies), while only one study focused on the root. The *Laportea* extracts were administered via two routes of administration, primarily oral, concentrated on evaluationg antinociceptive, anti-inflammatory, and antioxidant effects, whereas topical preparations predominantly assessed antinociceptive and anti-inflammatory effects.

**Table 1:** Characteristics of the studies

No.	Authors, year / country, (code),	Study design				Assessed markers	Main results
		*Laportea *species/ Part of plant/ Extraction / Route Administration	Animal species; strain (n/group)	Objective	Methodological		
1.	Dongmo, 2008/ Cameroon, (1-5) [31]	*L. ovalifolia*/ leaves/ methanol/ per oral/ 100;200;1000 mg/kg BW	Mice; Swiss albino (7)	Analgesic activities	a. Acetic acid-induced writhing	a. Frequency of writhing	a.↓ the number of writhings
					b. Formalin inj	b. Pain reaction time	b.100 and 200 mg/kg ↓ PRT
2.	Luo, 2011/ China, (1-3) [34]	*L.bulbifera*/ (Total coumarins isolated from *L. bulbifera* root (TC)/ / ethanol/ per oral/ 20; 40; 60 mg/kg BW	Mice; Balb/c (12)	Inflammatory effect	CFA inj. - induced arthritis:	TGF-β; IL-2; IL-10; IFN-γ	a.40 and 60 mg/kg BW TC ↑ IL-10 and TGF-β.
							b. All doses TC: ↓ IL-2, IFN-γ
3.	Islam, 2014/ Bangladesh, (1-6) [32]	*L.interrupea*/ leaves/ methanol/ per oral/ 25;50;100 mg/kgBW	Mice; Swiss albino (5)	Analgesic effect	a. Acetic acid-induced writhing test:	a. Frequency of writhing	a. ↓ the mean number of writhing
					b. Tail immersion test	b. Pain reaction time (PRT)	b. The PRT increased with the increase of doses
4.	Dapaah, 2015/ Ghana, (1-3) [33]	*L. ovalifolia*/ leaves/ methanol/ per oral/ 30; 100; 300 mg/kg BW	Rat; Sprague Dawley (4)	Inflammatory activity	Carrageenan Inj.	Reduction of oedema	Oedema ↓
5.	Njina, 2016/ Cameroon, (1-15) [15]	*L.ovalifolia*/leaves/ aquous/ per oral/ 100; 400; 800 mg/kg BW	Rat; Wistar (6)	Oxidative stress activity	Castrated rats were given simultaneously naphthalene solution (110 mg/kg, p.o.)	SOD; CAT; PO; NO	a.CAT ↑: 100; 400 mg/kg BW in lungs
							b. SOD ↑in heart with 100 mg/kg BW LO and 400 mg/kg BW in liver
							c. PO ↑ in lungs with 100 mg/kg BW
							d. 100 mg/kg BW ↓ NO in heart, lungs, and kidney. 400 mg/kg BW ↓ NO in liver and heart. 800 mg/kg BW ↓ NO in liver
6.	Ashidi, 2017/ Nigeria, (1-6) [12]	*L. aestuans (L) Chew*/ leaves/ ethanol/ per oral/ 50; 100; 150 mg/kg BW	Rat; Wistar (5)	analgesic and anti-inflammatory effect	a. Hot plate test	a. Pain reaction time	a. The extract has a significant and dose-dependent elevation of the PRT
					b. Carrageenan inj	b. Reduction of oedema	b. dose-dependent inhibition of carrageenan-induced oedema.
7.	Elizabeth, 2018/ Nigeria, (1-3) [23]	*L. aestuans*/ leaves/ methanol/ per oral/ 200; 400; 800 mg/kg BW	Rat; Wistar (6)	oxidative stress effect	Diclofenac - induced brain stress:	PCO; MDA; CAT; SOD; GSH	PCO, MDA ↓
8.	Omolola, 2018/ Nigeria, (1-3) [25]	*L. aestuans (L) Chew*/ leaves/ hydro-ethanol/ per oral/ 100; 200; 400 mg/kg BW	Rat; Wistar (5)	antioxidant effect	Prostatic rats	CAT; SOD; GSH	CAT, SOD, GSH ↑
9.	Onadeko, 2021a/ Nigeria, (1-2) [26]	*L.aestuans*/ ethanol/ per oral/ 200; 400 mg/kg BW	Rat; Wistar (4)	Stres oxidative response	Aspirin - induced ulcerated rats:	GSH, CAT	a. 200; 400 mg/kg BW ↑ GSH
							b. Only 200 mg/kg BW: ↑ CAT
10.	Onadeko, 2021b/ Nigeria, (1-2) [22]	*L.aestuans*/ ethanol/ per oral/ 200; 400 mg/kg BW	Rat: Wistar (4)	Stres oxudative response	Aspirin - induced ulcerated rats:	SOD; MDA	200; 400 mg/kg BW decreased MDA, but not increased SOD
11.	Simaremare, 2022/ Indonesia, (1-3) [28]	*L.decumana*/ leaves/ crude/ topical/ patch 0.38%; 1.11%; 1.8%	Mice, Mus musculus (5)	Analgesic effect	Acetic acid-induced writhing test	Frequency of writhing	All patch showed analgesic effect
12.	Tijani, 2022/ Nigeria, (1-2) [27]	*L.aestuans*/ leaves/ methanol/ per oral/ 200; 400 mg/kg BW	Rat: Wistar (8)	Inflammatory and oxidative stress activity	Gastric ulcer induced ethanol	a.TNF-α; IL-1β	a.TNF-α and IL-1β ↓
						b.SOD; CAT; GPx; MDA, GSH, GPx, GST	b.SOD, CAT, GPx ↑; MDA ↓
13.	Bambungan, 2023/ Indonesia, (1-3) [29]	*L.decumana* / leaves/ ethanol/ oral/ 250; 500; 1000 mg/kg BW	Mice, Mus musculus (5)	Analgesic affect	Acetic acid inj	Frequency of writhing	writhing ↓
14.	Ganiyu, 2023/ Nigeria, (1-2) [24]	*L.aestuans*/ leaves/ aquous/ per oral/ 50; 100; mg/kg BW	Mice; N/A (5)	Oxidative stress activity	Acetaminophen induced liver injury	CAT; SOD; GPx; GR	a.↑ CAT, SOD, GPx, and GR.
							b. Only 2% cream reduced paw edema IL-6 level
15.	Mewar, 2023/ Indonesia, (1-6) [13]	*L.decumana*/ leaves/ 70% ethanol/ topical/ cream 0.5%; 1%; 2%	Rat; N/A (5)	Inflammatory and analgesic effect	a. Formalin inj	a. Number of paw licking	a.↓ the paw licking
					b. Carrageenan Inj	b. Reduction of oedema; IL-6	b. Only 2% cream reduced paw edema IL-6 level
16.	La Basy, 2024/ Indonesia, (1-6) [30]	*L.decumana* / leaves/ ethanol/ oral/ 100; 200; 400 mg/kg BW	Mice; Balb/c (6)	Analgesic activity	a. Heat induction (hotplate)	a. Pain reaction time	a.↑ dose - dependent
					b. Acetic acid inj.	b.% inhibition	b.↑ dose – dependent

*Abbreviations*: *CAT* Catalase, *CFA* Complete Freund Adjuvan, *GSH* Glutathione, *GPx* Glutathione Peroxidase, *GR* Glutathione Reductase, *GST* Glutathione transferase,  *IFN-γ* Interferon Gamma, *IL* Interleukin, *MDA* Malondialdehyde, *NO* Nitrite Oxidase, *PCO* Protein Carbonylation, *PO* Peroxidase, *PRT* Pain Reaction Time, *SOD* Superoxida dismutase, *TC* Total Coumarin, *TGF-β* Transforming Growth Factor – Beta, *TNF-α* Tumor Necrosis Factor – alpha, ↑ increase, ↓ decrease

Furthermore, the studies utilized various extraction solvents, including ethanol, methanol, and aqueous solutions. Dosages varied substantially, ranging from 20 mg/kg to 1000 mg/kg body weight for oral preparations, and 0.5% to 2% concentrations for topical applications. The animal models employed were principally rodents, comprising rats (*Rattus norvegicus*) in nine studies and mice (*Mus musculus*) in seven studies. Specific rat strains included the *Wistar* strain (4 studies) and the *Sprague-Dawley* strain (1 study), with four studies reporting only the species name. Mice strains included *Balb/c* (2 studies) and Swiss albino (2 studies), with three studies lacking specific strain information. Sample sizes across the studies ranged from 5 to 12 animals per group. The observed heterogeneity, which is attributable to factors such as *Laportea* species, dosage variations, extraction methods, and the specific animal model utilized, was subsequently investigated through subgroup meta-regression analyses.

### Assessment

#### Methods for assessing antinociceptive effects

Antinociceptive effects were assessed using the formalin injection method, acetic acid injection (5 studies), tail immersion test (1 study), and hot plate test (1 study). The outcomes measured included the number of paw licks or leg movements, as well as pain reaction time (PRT). A reduction in paw licking or movement (negative effect size) or an increase in PRT (positive effect size) indicated an antinociceptive effect.

#### Methods for assessing anti-inflammatory effects

Anti-inflammatory assessments were conducted using methods such as carrageenan injection, induction of gastric ulceration with 70% ethanol, and injection of type II collagen in complete Freund’s adjuvant (CFA). Key markers assessed included paw edema, inflammatory cytokines: Tumor Growth Factor – Beta (TGF-β), Tumor Necrosis Factor – alpha (TNF-α), Interferon Gamma (IFN – γ), Interleukin (IL-2, IL-10, IL-1β, IL-6). *Laportea* species have an anti-inflammatory effect, it they can reduce the levels of pro-inflammatory markers (Interleukin (pg/ml), IFN – γ (pg/ml), TNF-α (pg/ml)), dan increase anti inflammatory levels (TGF-β (pg/ml), IL-10 (pg/ml)).

#### Methods for assessing antioxidant effects

Markers of oxidative stress evaluated included antioxidant enzyms (e.g., catalase [CAT], superoxide dismutase [SOD], glutathione [GSH], glutathione peroxidase [GPx], glutathione reductase [GR], and glutathione transferase [GST]), and tissue damage markers (e.g., malondialdehyde [MDA], nitrite oxide [NO], protein carbonylation [PCO], peroxidase). A negative effect size indicated oxidative stress reduction by decreased levels of MDA (nmol/mg protein), NO (ng/mg protein), and PCO (µmol/mg protein), and peroxidase, while a positive effect size reflected antioxidant effects with increased levels of CAT (nmol/mg protein), SOD (µmol/mg protein), GPx (nmol/mg protein), GR (nmol/mg protein), and GST (nmol/mg protein).

### Coded studies for meta-analysis

The 16 selected studies contributed a total of 70 coded studies, which were incorporated into the meta-analysis dataset. These coded studies were categorized based on difference dosages and methods for assessing antinociceptive effects, as well as the parameters used to evaluate anti-inflammatory and antioxidant effects. Study codes in the form of numbers pinned after author’s (Table 1).

### Risk of bias

**Fig. 2:**
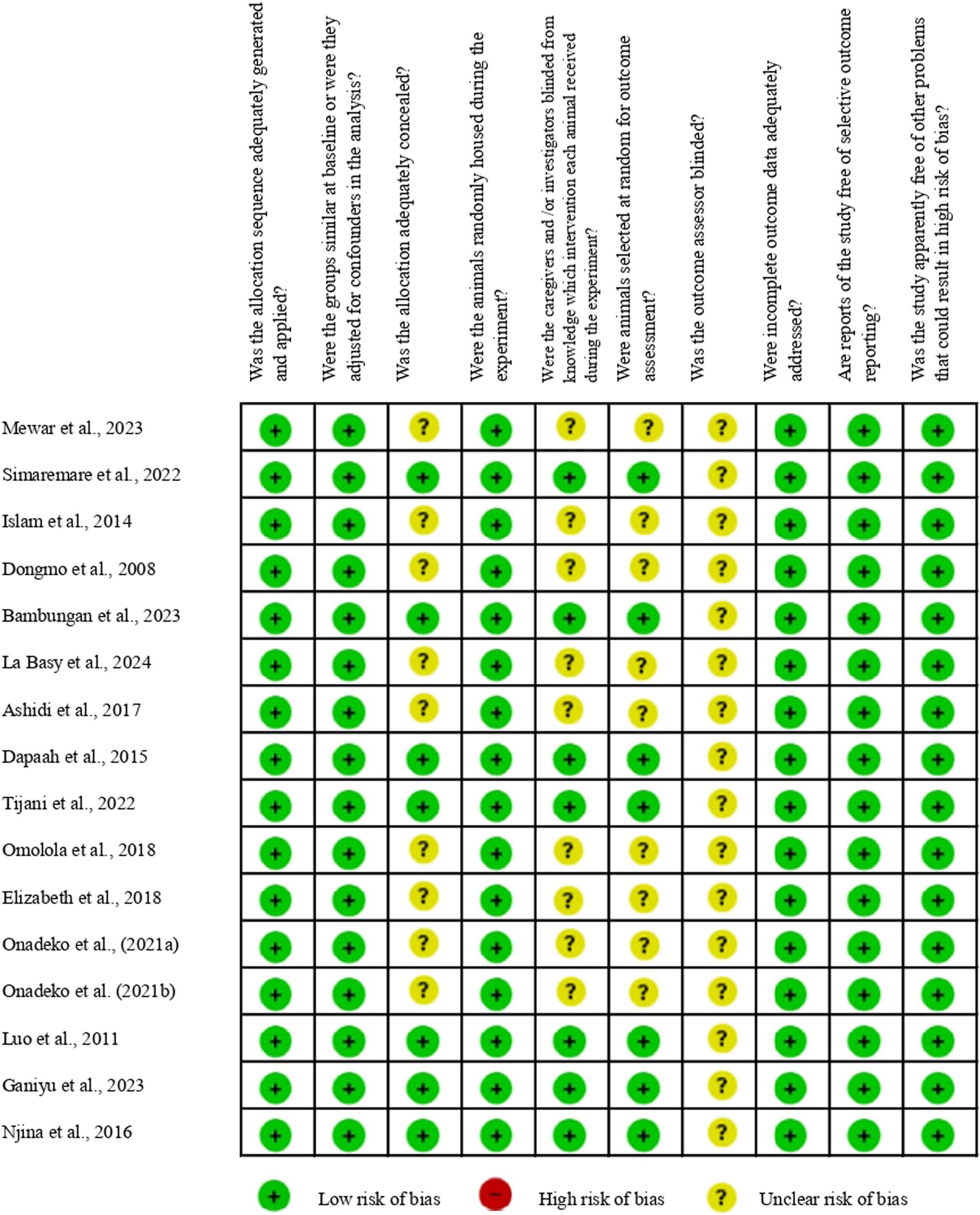
Risk of bias summary

Figure 2 summarizes the risk of bias for the sixteen included studies, where most studies demonstrated a low risk in key methodological areas such as sequence generation, baseline characteristics, random housing, and addressing incomplete outcome data.

**Fig. 3:**
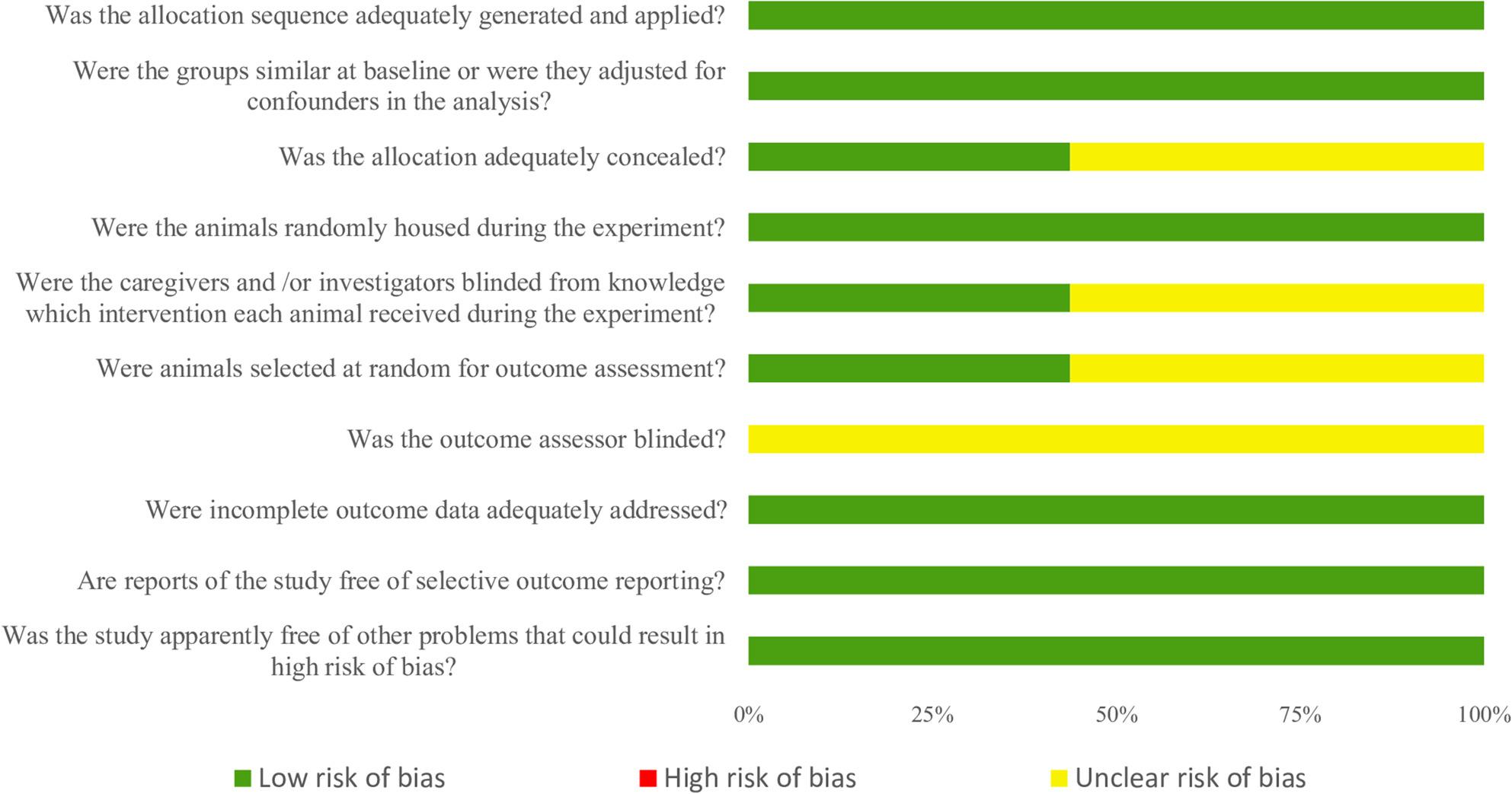
Risk of bias graph

However, the overall methodological quality assessment using the SYRCLE/CAMARADES Risk of Bias tool ultimately revealed a critical deficiency in the reporting of key experimental details, rather than an explicit indication of poor conduct, as no study was categorized as high risk in any domain (Fig. 3). Specifically, a high frequency of “unclear risk” was observed across several domains, including allocation concealment (50%), blinding of caregivers/investigators (50%), random outcome assessment (50%), and universally for blinding of outcome assessor (100%). This pervasive lack of explicit reporting regarding blinding, particularly the complete absence of reported outcome assessor blinding, introduces a substantial risk of detection bias, which has the potential to lead to an overestimation of the reported pharmacological effects and consequently undermine the internal validity of the analgesic results.

### Data synthesis

The meta-analysis outcomes were categorized into three major groups: antinociceptive, anti-inflammatory, and antioxidant effects, to evaluate the efficacy of *Laportea* species across different pharmacological activities. These effects were further analyzed based on the mode of preparation (topical vs. oral), reflecting the common administration routes in traditional use.

#### Antinociceptive

##### Oral antinociceptive effects of *Laportea* species extracts

This analysis presents a meta-analysis concerning the oral antinociceptive effects (pain-relieving properties) of *Laportea* species extracts. The analysis was structured into two separate meta-analyses based on the type of measured outcome. Negative effect sizes were assessed using indicators of pain reduction, such as the frequency of writhing or time spent licking. Conversely, positive effect sizes were assessed by measuring pain tolerance, specifically the Pain Reaction Time (PRT).

##### Analysis of writhing frequency

**Fig. 4:**
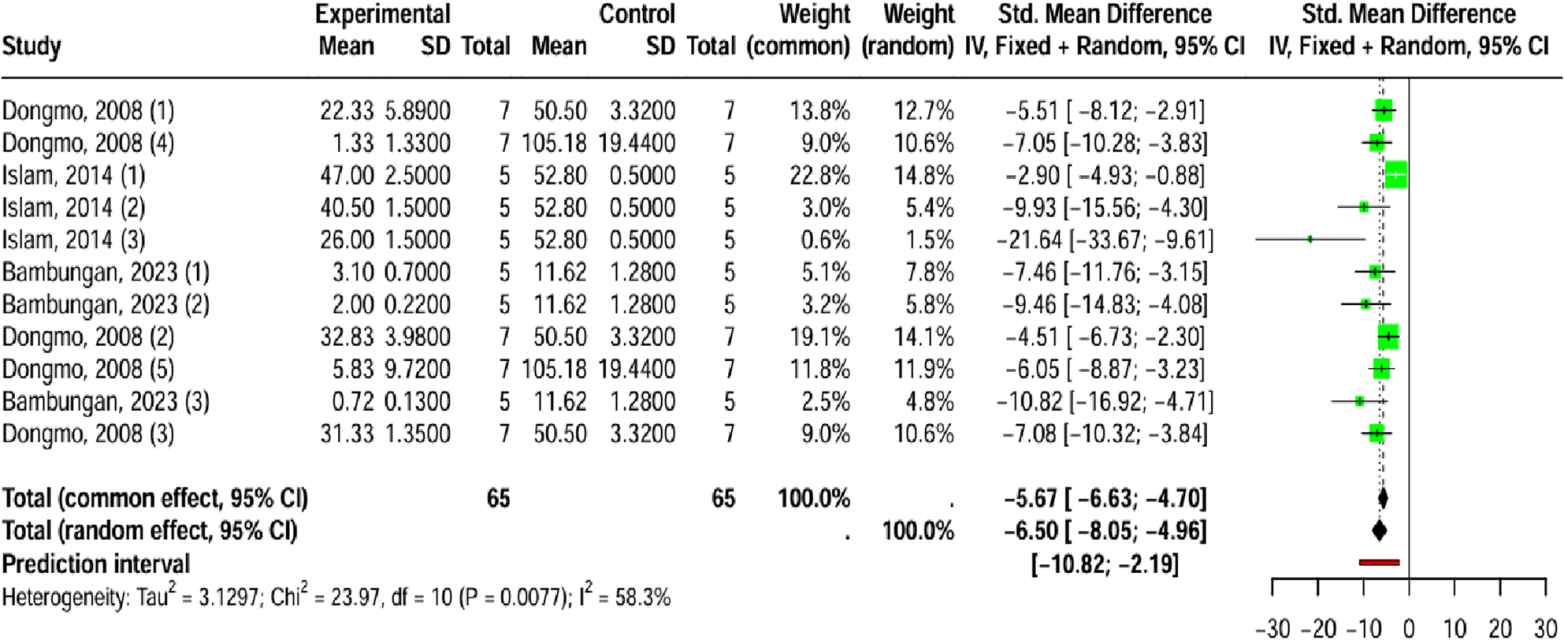
Forest plot depicting the effect of *Laportea* species on pain, measured by the frequency of writhing outcome

The analysis of writhing frequency, a proxy for acute pain, demonstrated a strong and significant reduction in pain response following *Laportea* extract administration. The pooled result from 130 subjects, using a random-effects model (I² = 58.3%; *p* = 0.007), showed a large negative effect size (SMD = -6.50; 95% CI [-8.05 to -4.96]; *p* < 0.0001), confirming a potent antinociceptive effect (Fig. 4).

Although Egger’s test indicated potential asymmetry/publication bias (t = -9.30; *p* < 0.0001) (See Additional File 2A-2B), the subsequent mixed-effects meta-regression suggested that the tested experimental covariates (*Laportea* species, extraction method, duration of therapies, dosage, study methods, tissue used) failed to significantly explain the observed heterogeneity (R^2^ = 40.06%; Q_M_: *p* = 0.20). This implies that the efficacy of *Laportea* extract in reducing writhing frequency is robust and consistent across the tested experimental variations (See Additional File 2C).

##### Analysis of pain reaction time (PRT)

**Fig. 5:**
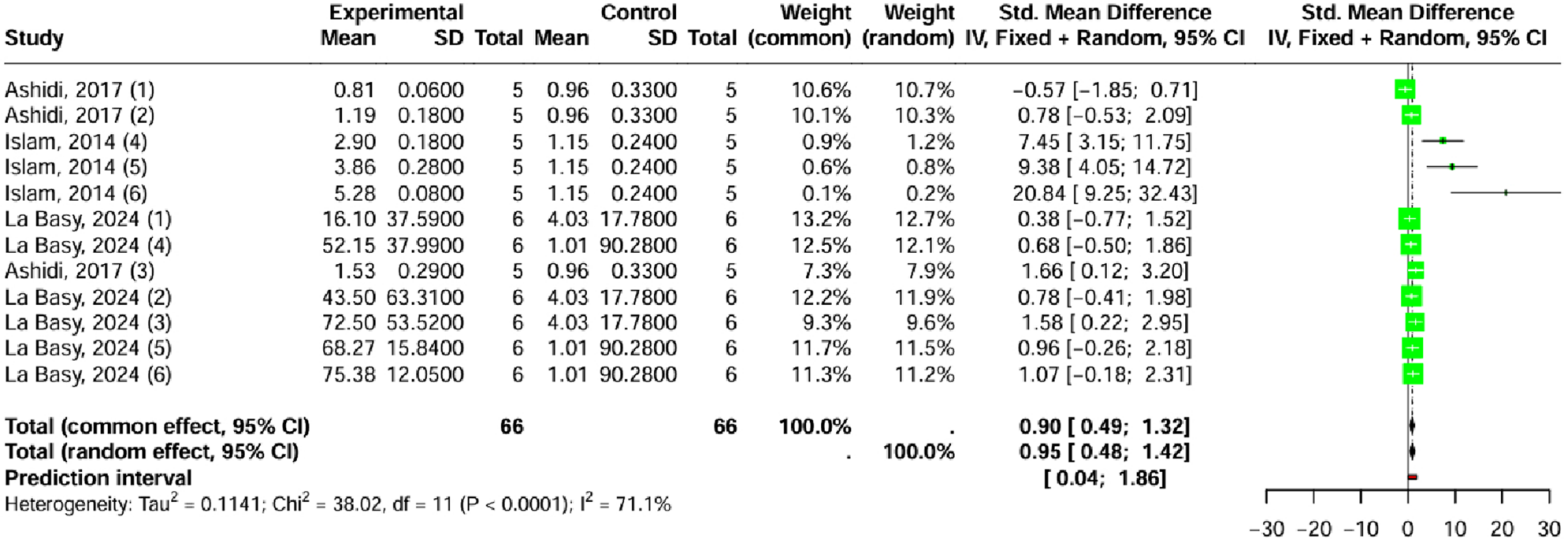
Forest plot depicting the effect of *Laportea* species extract on pain, measured by the pain reaction time outcome

The meta-analysis of PRT, which assesses increased pain tolerance, also found a significant positive effect. The random-effects model (I² = 71.1%; *p* = 0.0001) yielded a pooled effect size (SMD = 0.95; 95% CI [0.48 to 1.42]; *p* = 0.0001) across 132 subjects, confirming the extract’s ability to significantly prolong the time required to react to a painful stimulus (Fig. 5).

Funnel plots showed asymmetry and supported by the significant results of Egger’s test (t: 7.26, *p* < 0.0001) (See Additional File 3A-3B). To account for the high level of variability (I² = 71.1%; *p* = 0.0001), a mixed-effects meta-regression was conducted, incorporating moderators such as *Laportea* species, extraction method, animal species, dosage, studies method, and tissue used (with therapy duration consistent across studies). In contrast to the writhing analysis, the meta-regression for PRT revealed that the experimental covariates significantly contributed to the observed heterogeneity (R^2^ = 100%; Q_M_, *p* < 0.0001). Three variables were identified as highly significant predictors of the effect size: the extraction method, the study method (type of pain assay), and the tissue used (site of pain application).

Analysis of individual coefficients revealed three highly significant predictors: the extraction method, studies method and tissue used, all demonstrating a strong positive association with the study effect size (β = 1.36; *p* < 0.0001) (See Additional File 3C). These categorical factors were subsequently investigated through dedicated subgroup analyses.

Dedicated subgroup analyses confirmed that the antinociceptive effect was significantly stronger subgroups (*p* = 0.0005), under specific conditions: methanol extraction (SMD = 10.37; 95% CI [4.95; 15.78]) yielded a far greater effect than ethanol extraction (SMD = 0.76; 95% CI [0.34; 1.18]); the tail immersion method (SMD = 10.37; 95% CI [4.95; 15.78]) was more potent than the hot plate test (SMD = 0.76; 95% CI [0.34; 1.18]); and testing on tail tissue (SMD = 10.37; 95% CI [4.95; 15.78]) resulted in a stronger effect than paw tissue (SMD = 0.76; 95% CI [0.34; 1.18]) (See Additional File 3D-3F).

These subgroup findings underscore that the antinociceptive effectiveness of *Laportea* is highly conditional on specific experimental parameters, particularly the choice of extraction method, the type of pain assay, and the target tissue used.

##### Topical antinociceptive

**Fig. 6:**
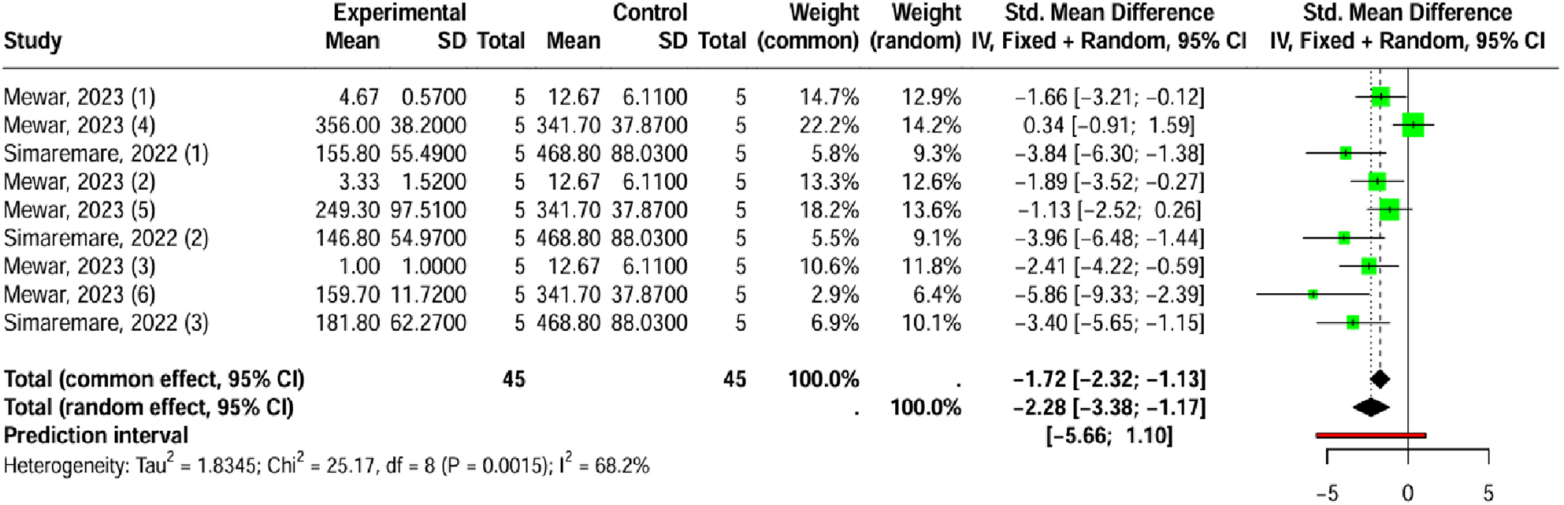
Forest plot of the effect of *Laportea* species extract on pain administered topically

According to a random-effects model (I² = 68.2%, *p* = 0.0015), *Laportea* species extract applied topically significantly reduced pain (SMD = -2.28; 95% CI [-3.38 to -1.17]; *p* < 0.001) (Fig. 6). Publication bias could not be performed because the analysis included fewer than ten trials.

To understand the substantial variation observed between studies (I² = 68.2%, *p* = 0.0015), a meta-regression analysis was performed. Potential sources of heterogeneity investigated included the extraction technique, animal species, therapy duration, study methodology, topical formulation, and therapeutic dose. The analysis revealed that the collective set of covariates significantly contributed to explaining the observed heterogeneity (R^2^ = 98.89%; Q_M_, *p* < 0.0009). This is an important finding, as it means almost all the differences in outcomes among studies are due to differences in their experimental design.

Analysis of individual coefficients revealed three highly significant predictors and demonstrated a strong positive association with the study effect size: the extraction method (β = 0.88; *p* < 0.003), animal species (β = 17.18; *p* < 0.04), and topical formulation (β = 10.28; *p* < 0.04) (See Additional File 4A). The analysis will be to conduct dedicated subgroup analyses for each of these significant categorical moderators.

Dedicated subgroup analyses confirmed the profound influence of these three factors. Regarding the extraction method, a significant difference was observed (*p* = 0.028), where crude extraction (SMD = -3.71; 95% CI [-5.1; -2.32]) was found to be more potent than ethanol extraction (SMD = -1.66; 95% CI [-2.87; -0.46]). Similarly, the form of topical application also showed a significant difference (*p* = 0.028); the extract in a patch formulation (SMD = -3.71; 95% CI [-5.1; -2.32]) provided a stronger pain reduction compared to the cream formulation (SMD = -1.66; 95% CI [-2.87; -0.46]). Significant variation was noted based on the experimental subject (*p* = 0.028), with studies using mice (SMD = -3.71; 95% CI [-5.1; -2.32]) reporting a stronger antinociceptive effect than those using rats (SMD = -1.66; 95% CI [-2.87; -0.46]) (See Additional File 4B-4D).

#### Inflammatory effect

The inflammatory effects were analyzed in two subgroups based on the method of administration: oral and topical.

##### Oral anti-inflammatory effect

The inflammatory effects of *Laportea* species extract were analyzed by categorizing oral administration outcomes into two groups: the reduction of pro-inflammatory markers (such as edema size, IL-2, IFN-γ, TNF-α, and IL-1β) and the promotion of anti-inflammatory markers (including IL-10 and TGF-β).

##### Reduction of pro-inflammatory markers

According to a random-effects model (I² = 82.5%, *p* = 0.0001), *Laportea* species significantly reduced overall inflammation (SMD = -2.62; 95% CI [-3.99 to -1.24]; *p* < 0.0002). Futher analysis revealed significant differences between the parameters (*p* = 0.0001). The most substantial effect was the significant decrease in IL-1β levels (SMD = -8.77; 95% CI [-12.43; -5.10]; *p* < 0.0001; I² = 47.7). This was followed by significant reductions in TNF-α level (SMD = -4.63; 95% CI [-8.72; -0.53]; *p* = 0.026: I² = 84.4%), IFN-γ levels (SMD = -1.28; 95% CI [-1.80; -0.76], *p* < 0.0001; I² = 0%), and IL-2 levels (SMD = -0.75; 95% CI [-1.23; -0.26]; *p* = 0.0025; I² = 0%) compared to the control group.

**Fig. 7:**
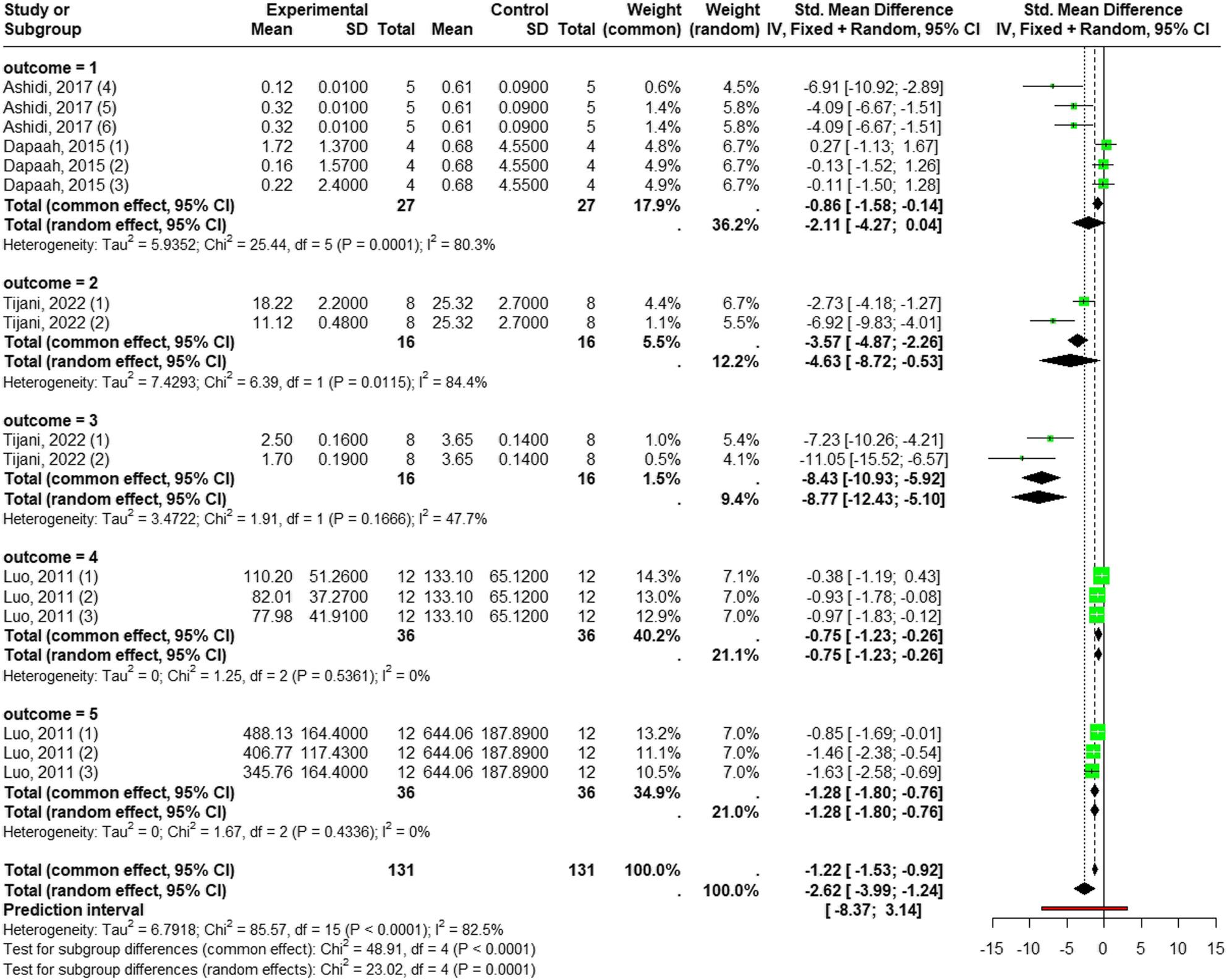
Forest plot of *Laportea* species extract effect on pro-inflammatory outcomes: outcome = 1: oedema size; outcome = 2: TNF-α; outcome = 3: IL-1β; outcome = 4: IL-2; outcome = 5; IFN-γ

Conversly, when assessing physical inflammatory outcomes, the *Laportea* species extract did not significantly reduce paw’s oedema size compared to the control group (SMD = -2.11, 95% CI [-4.27; 0.04], *p* = 0.05; I² = 80.3%) (Fig. 7).

Substantial heterogeneity was exclusively found in TNF-α level analysis (I² = 84.4%; *p* = 0.0115), where dosage was the only one different variable. A dedicated subgroup analysis was thus performed for TNF-α and confirmed significant differences between dose groups (*p* = 0.0115). Specifically, a dose of 400 mg/kg BW (SMD = -6.92) was found to be more potent in lowering TNF-α levels than a dose of 200 mg/kg BW (SMD = -2.73), underscoring a dose-dependent effect for TNF-α (See Additional File 5).

Publication bias was not assessed for any cytokine outcome, due to the inclusion of fewer than ten studies were included, nor for the oedema outcome, which did not yield statistically significant results.

##### Promotion of anti-inflamatory markers

**Fig. 8:**
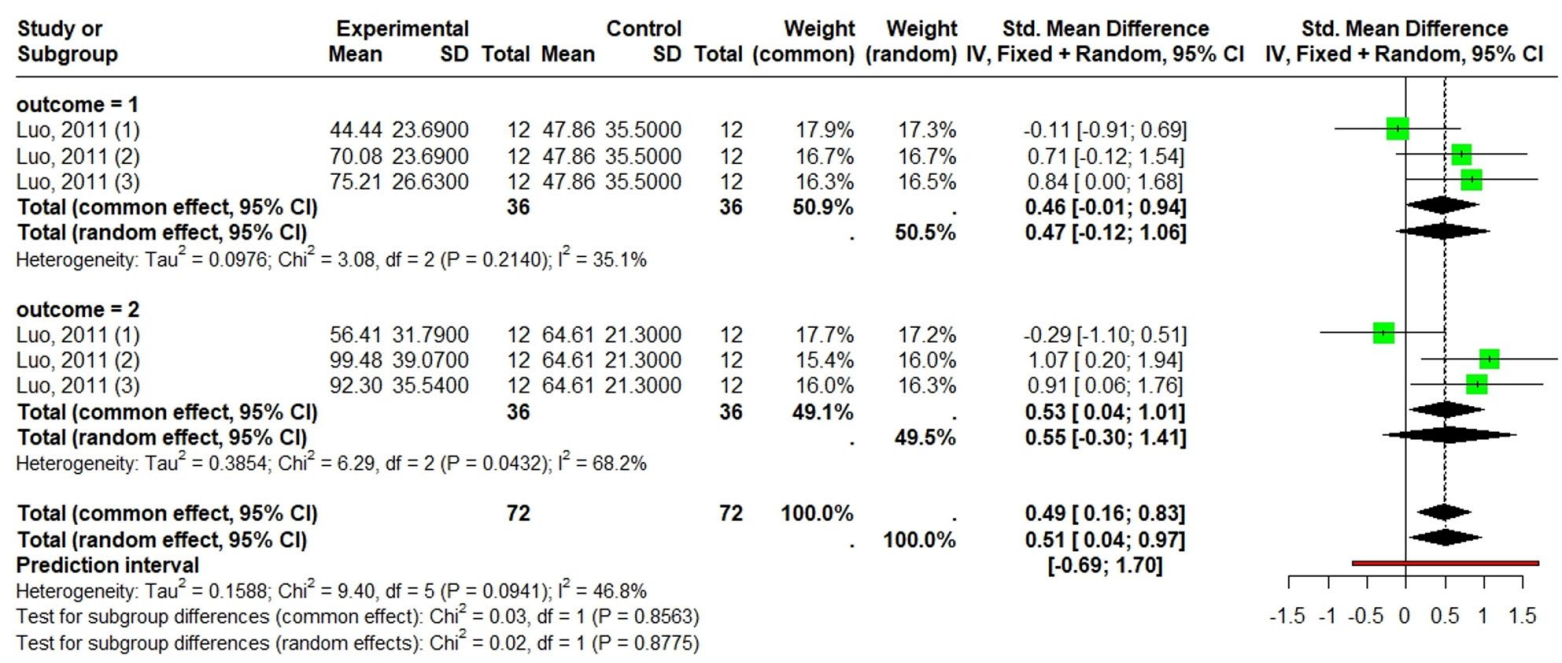
Forest plot of *Laportea* species extract effect on anti-inflammatory paramaters: outcome = 1:IL-10 outcome = 2: TGF-β

According to a fixed-effects model (I² = 46.8%, *p* = 0.09), *Laportea* species significantly reduced inflammation with increasing anti-inflamatory markers (SMD = 0.49; 95% CI [0.15; 0.83]; *p* < 0.0043). Futhtermore, there were no significant differences (*p* = 0.86), between the effects on TGF-β (SMD = 0.55) and IL-10 (SMD = 0.46) (Fig. 8).

Publication bias and meta-regression were not assessed due to the low number of included studies and low heterogeneity, respectively.

##### Topical inflammatory effects

The topical application of *Laportea* species extract also demonstrated a significant overal reduction in inflamation. Using a random-effects model (I² = 62.3%, *p* = 0.02), the pooled analyis yielded effect size (SMD = -1.10; 95% CI [-2.16; -0.04]; *p* < 0.0043).

**Fig. 9:**
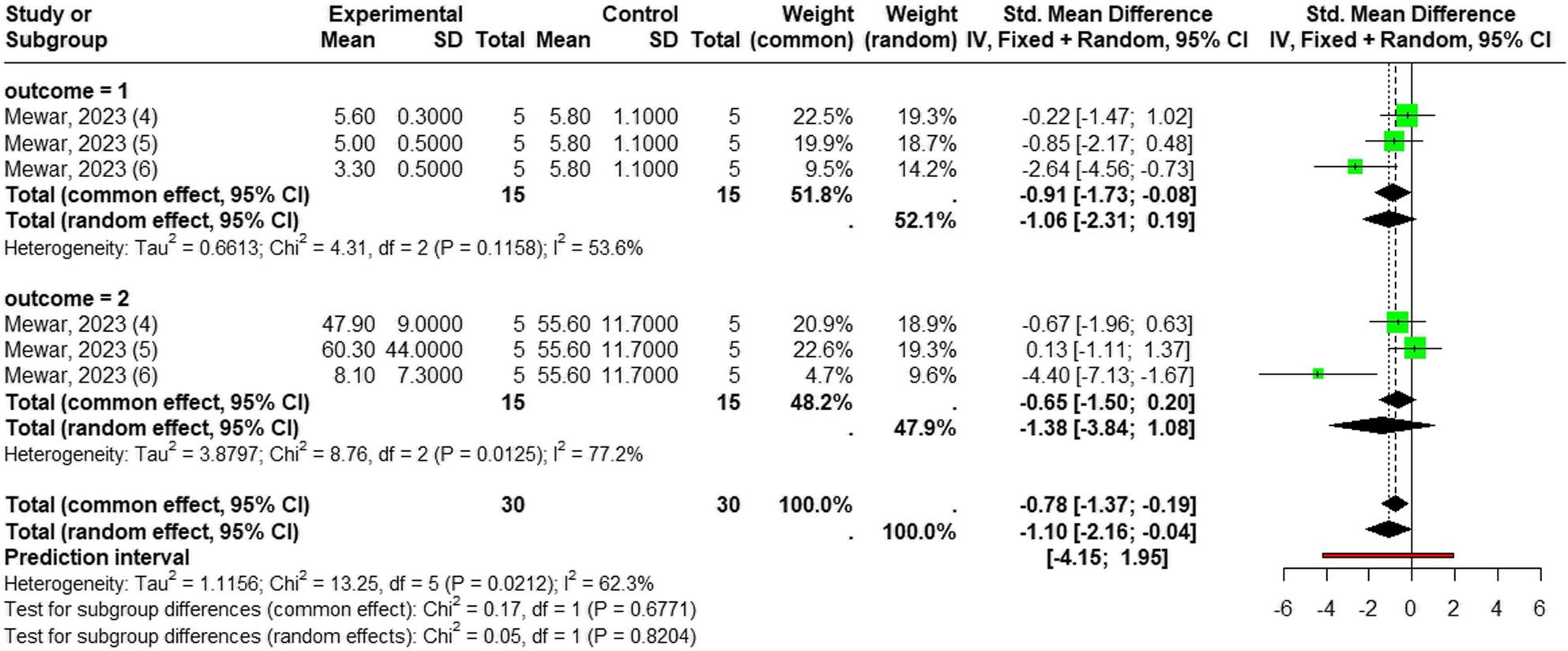
Forest plot of *Laportea* species extract effect on inflammatory outcomes administrated topically: outcome = 1: Il-6; outcome = 2: paw’s oedema size

There was no difference between IL-6 and paw oedema size (*p* = 0.82), although the result of each parameters were not significant: IL-6 levels (*n* = 30; SMD = -1.06; 95% CI [-2.31; 0.19], *p* = 0.096, I² = 53.6% ), and paw oedema size (*n* = 30; SMD = -1.38, 95% CI [-3.84; 1.08], *p* = 0.27; I² = 77.2%) (Fig. 9).

Despite the lack of significance in the individual paw edema outcome, moderate to high heterogeneity was detected (I² = 77.2%; *p* = 0.012), and at Il-6 levels ((I² = 53.6%; *p* = 0.11). Since dosage was the only variable that differed across these specific trials, a subgroup analysis was conducted. This analysis revealed a significant difference between dose groups: a topical preparation dose of 2% was shown to significantly reduce paw edema size and IL-6 level (SMD = -3.24), while lower concentrations of 1% and 0.5% did not achieve statistical significance. This strongly suggests a dose-dependent threshold required for the topical anti-edema and IL-6 level effect to manifest (See Additional File 6).

Publication bias was not assessed due to the limited number of studies.

#### Oxidative stress effect

The analysis of the extract’s effect on oxidative stress was divided into two areas: cell damage markers (PCO, MDA, and NO) and antioxidant parameters (including GSH, GR, GST, GPx, SOD, CAT, and peroxidase).

##### Reduction of cell damage markers

The pooled analysis of cell damage markers revealed that *Laportea* species extract significantly reduced oxidative stress by lowering the levels of these markers. Utilizing a random-effects model (I² = 77.7%, *p* = 0.0001), the overall effect yielded a negative SMD (SMD = -2.42; 95% CI [-3.17; -1.68]; *p* < 0.0001).

**Fig. 10:**
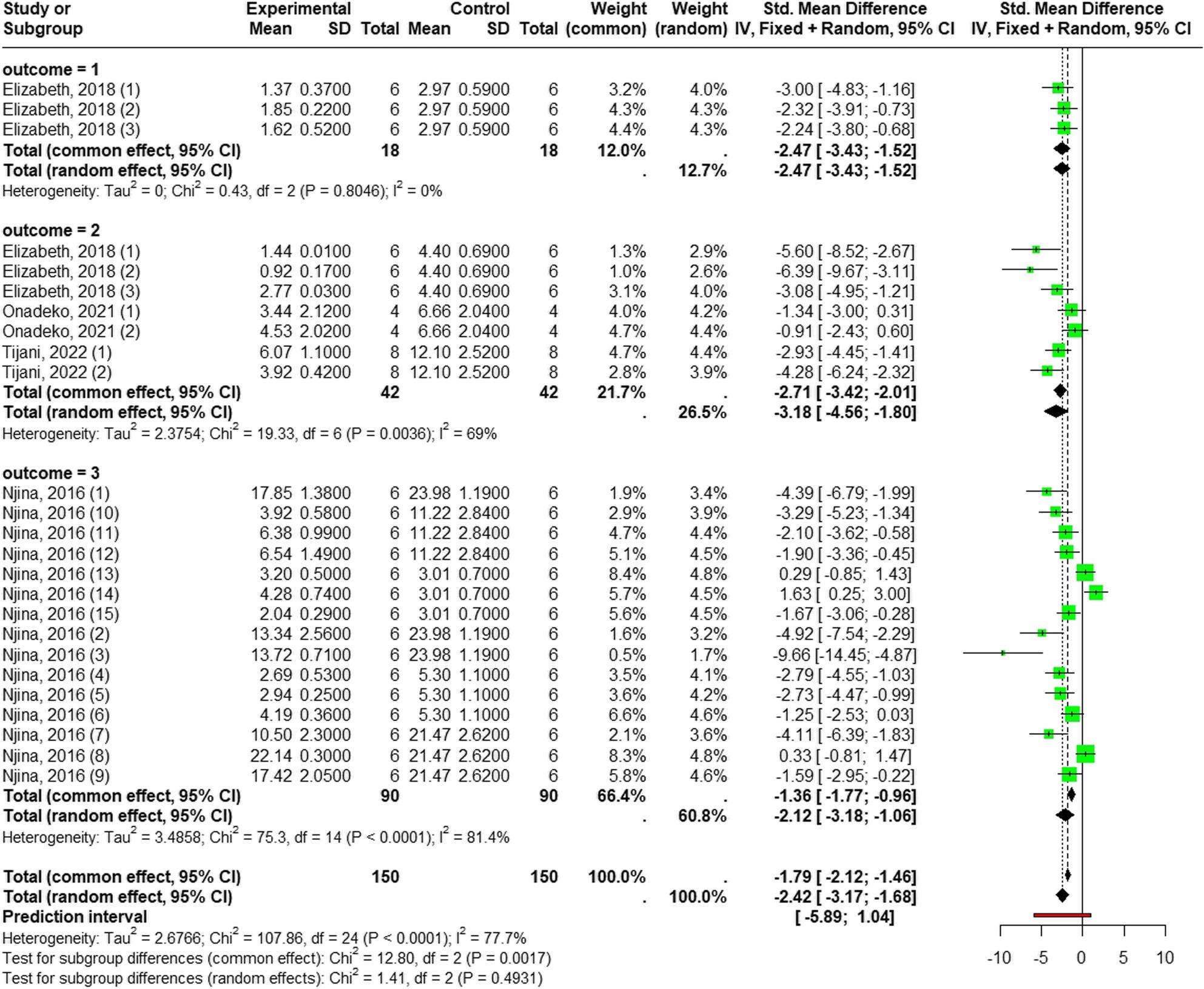
Forest plot of *Laportea* species extract effect on cell damage caused by oxidative stress: outcome = 1: PCO; outcome = 2: MDA; outcome = 3: NO

There was no significant difference detected in the overall effect magnitude among the three individual markers (*p* = 0.49), though each marker showed a highly significant reduction: PCO (*n* = 36; SMD = -2.47; 95% CI [-3.43; -1.52]; *p* < 0.0001; I² = 0% ), MDA (*n* = 84; SMD = -3.18, 95% CI [-4.56; -1.79]; *p* < 0.0001; I² = 69% ) and NO (*n* = 180; SMD = -2.12; 95% CI [-3.18; -1.06]; *p* < 0.0001); I² = 81.4%) (Fig. 10).

Publication bias was assessed only for NO, as fewer than ten studies were included for PCO and MDA. The funnel plot for NO indicated asymmetry, statistically comfirmed by a highly significant of Egger’s test (t = − 6.54, p-value < 0.0001) (See Additional File 7A, 7B). Meta-regression analysis was conducted only for the NO (I² = 81.4%; *p* = 0.0001) and MDA (I² = 69%; *p* = 0.0036) due to their significant heterogeneity.

To investigate the causes of NO heterogenity, a meta-regression analysis was performed using the following variables: dosage, and tissue used. The mixed-effects models revealed that the covariates significantly contributed to the heterogeneity observed in this analysis (R^2^ = 39.89%; Q_M_, *p* = 0.0078). Analysis of individual coefficients revealed only tissue used highly significant predictors, demonstrating a strong positive association with the study effect size (β = 0.33; *p* < 0.002) (See Additional File 7C). Subgroup analysis across different tissues (liver, heart, lungs, kidneys, serum) revealed significant differences (*p* = 0.005). Further findings demonstrated that *Laportea* species extract significantly reduced NO levels in the liver (SMD = -5.50; 95% CI [-7.71; -3.29]), kidneys (SMD = -2.28; 95% CI [-3.21; -1.36]), and heart (SMD = -2.11; 95% CI [-3.21; -1.01]), but no significant reduction was observed in the lung and serum tissues (See Additional File 7D).

For MDA, the tested covariates (dose, extract type, method, duration, tissue used) significantly contributed to explaining the heterogeneity (R^2^ = 92.54%; Q_M_, *p* < 0.026). The analysis of individual coefficients revealed that the extraction method was a highly significant predictors (β = 0.94; *p* < 0.012) (See Additional File 8A).

A dedicated subgroup analysis comparing extraction methods found a significant difference (*p* = 0.0004), methanol extract significantly reduced MDA levels (SMD = -3.94, 95% CI [-5.05; -2.82]), while ethanol extract did not show a significant reduction (SMD = -1.10, 95% CI [-2.22; 0.01]) (See Additional File 8B).

##### Enhancement of antioxidant markers

The overall meta-analysis of antioxidant parameters (including GSH, GPx, GR, GST, SOD, CAT, Peroxidase) demonstrated that *Laportea *significantly reduced oxidative stress by increasing the activity of these endogeneous markers (SMD = 2.77; 95% CI [2.11; 3.42]; *p* < 0.0001). *Laportea* species significantly reduced oxidative stress by increasing antioxidant markers.

Utilizing a random-effects model, the overall differences between the markers were marginally insignificant (*p* = 0.05). High levels of activity were observed across several enzyms, notably GPx level (*n* = 52; SMD = 4.38; 95% CI [1.08; 7.68]; *p* = 0.01; I² = 86.8%), GR levels (*n* = 20; SMD = 4.09; 95% CI [2.22; 5.95]; *p* < 0.0001; I² = 8.4%), GST levels (*n* = 52; SMD = 4.08; 95% CI [2.95; 5.21]; *p* < 0.0001; I² = 49.1%), CAT levels (*n* = 298; SMD = 2.73; 95% CI [1.61; 3.86]; *p* < 0.0001; I² = 84.2%); SOD levels (*n* = 314; SMD = 2.72; 95% CI [1.21; 4.22]; *p* = 0.0004; I² = 84%), peroxidase levels (*n* = 180; SMD = 1.92; 95% CI [1.29; 2.55]; *p* < 0.0001; I² = 61%), and GSH levels (*n* = 98; SMD = 2.89; 95% CI [-1.94; 7.73]; *p* = 0.24, I² = 91.2%) (See Additional File 9).

Meta-regression analysis was conducted for the GPx (I² = 86.8%; *p* = 0.0001), CAT (I² = 84.2%; *p* = 0.0001); SOD (I² = 84%; *p* = 0.0001), Peroxidase (I² = 61%; *p* = 0.001), and GSH levels (I² = 91.2%; *p* = 0.0001).

To investigate the causes of SOD heterogenity, a meta-regression analysis was performed using the following variables: *Laportea* species, treatment duration, extraction method, dosage, studies methods, animal species, and tissue used. The mixed-effects models revealed that the covariates significantly contributed to the heterogeneity observed in this analysis (R^2^ = 91.81%; Q_M_, *p* < 0.0001). Analysis of individual coefficients revealed four moderators highly significant predictors, demonstrating a strong positive association with the study effect size: *Laportea* species (β = 2.68; *p* < 0.0001), duration treatment (β = 17.31; *p* < 0.0001), extraction method (β = 2.67; *p* < 0.0001), and studies methods (β = 1.34; *p* < 0.0001) (See Additional File 10A).

The subgroup analysis revealed significant differences among *Laportea* species (*p* = 0.0122), with *L. aestuans* exhibiting a stronger effect in increasing SOD levels (SMD = 5.63; 95% CI [1.96; 9.32]) compared to *L. ovalifolia* (SMD = 0.87; 95% CI [0.3; 1.45]) (See Additional File 10B). Subgroup analysis based on methodology showed significant differences (*p* < 0.0001), where *Laportea* species increased SOD levels significantly in aspirin-induced ulcerative models, Benign Prostate Hipertrophy (BPH) models, and castrated rat models (SMD = 9.33; 95% CI [6.61; 12.06]; SMD = 5.34; 95% CI [2.99; 7.69]; SMD = 0.87; 95% CI [0.29; 1.45], respectively) (See Additional File 10C). Subgroup analysis by treatment duration revealed significant differences (*p* = 0.0021), with treatment for 4–7 days showing the strongest increase in SOD levels (SMD = 9.31; 95% CI [4.76; 13.86]) compared to treatment beyond 7 days (SMD = 1.30; 95% CI [0.58; 2.01]). No significant increase in SOD levels was observed for duration treatment of 1–3 days (See Additional File 10D). In subgroup extract (*p* < 0.0001), *Laportea* methanol extract increased SOD level more potent (SMD = 9.00; 95% CI [7.09; 11.05] than ethanol and aqueous extract ((SMD = 3.22; 95% CI [0.47; 5.97]); (SMD = 0.88; 95% CI [0.25; 1.51], respectively)) (See Additional File 10E).

To investigate the causes of CAT heterogenity, a meta-regression analysis was performed using the following variables: dose, extract type, method, duration, animal species, *Laportea* species, and tested tissue. The mixed-effects models revealed that the covariates significantly contributed to the heterogeneity observed in this analysis (R^2^ = 52.81%; Q_M_, *p* < 0.0001). Analysis of individual coefficients revealed five moderators highly significant predictors, demonstrating a strong positive association with the study effect size: *Laportea* species (β = 22.42; *p* < 0.0001), duration treatment (β = 10.24; *p* < 0.0001), extraction method (β = 57.61; *p* < 0.0001), and studies methods (β = 14.63; *p* < 0.0001) (See Additional File 11A).

Regarding treatment duration, significant differences were observed (*p* = 0.0325), with short-term treatment (1–3 days) yielding the highest increase in CAT levels (SMD = 18.54, 95% CI [3.36; 33.73]) compared to medium (4–7) days (SMD = 3.55, 95% CI [2.33; 4.77]) and beyond 7 days (SMD = 2.15, 95% CI [1.01; 3.28]) (See Additional File 11B). In the extract type subgroup, significant differences were found (*p* = 0.0002), with methanol extract showing the strongest effect on increasing CAT levels (SMD = 19.07, 95% CI [4.70; 33.43]) compared to ethanol extract (SMD = 5.55, 95% CI [3.59; 7.51]) and aqueous extract (SMD = 1.62, 95% CI [0.63; 2.61]) (See Additional File 11C).

Analysis of *Laportea* species also revealed significant differences (*p* = 0.0153), with *Laportea aestuans* demonstrating a stronger effect (SMD = 10.23, 95% CI [3.34; 17.12]) in increasing CAT levels compared to *Laportea ovalifolia* (SMD = 1.61, 95% CI [0.54; 2.68]) (See Additional File 11D). In the subgroup studies methods, revealed significant differences between the study methods (*p* < 0.0001), with Diclofenac induced rat method showed the highest CAT levels (SMD = 31.26; 95% CI [22.46; 40.06] than the other methods (See Additional File 11E).

Meta-regression for GSH levels also indicated the tested factors (dose, extract type, method, duration, tested tissue) significantly contributed to heterogeneity (R^2^ = 64.34%; Q_M_, *p* < 0.005). Analysis of individual coefficients revealed four moderators highly significant predictors, demonstrating a strong positive association with the study effect size: tissue used (β = 1.79; *p* = 0.001), duration treatment (β = 5.67; *p* = 0.012), extraction method (β = 10.82; *p* < 0.003), and studies methods (β = 4.09; *p* = 0.042) (See Additional File 12A).

Dedicated subgroup analyses confirmed the influence of these factors. Significant differences were observed base on tissue type (*p* = 0.0002), where the highest increase in GSH levels was found in serum (SMD = 8.87; 95% CI [5.91;11.84]) compared to the brain (SMD = 2.90; 95% CI [1.85; 3.95]), with no significant effect in the gastric tissue (See Additional File 12B).

Futhermore, the treatment duration showed significant differences (*p* = 0.0002), prolonged treatment (> 7 days) resulted in the highest increase (SMD = 8.87; 95% CI [5.91;11.84] (See Additional File 12C). Regarding preparation, significant differences were found in extraction type subgroup (*p* = 0.003). The ethanol extract showed the strongest effect on increasing GSH levels (SMD = 8.87, 95% CI [5.91; 11.84]), in contrast to methanol extract which was non-significant (See Additional File 12D). Finally, the increase in GSH levels was maximazed within in BPH rat model (SMD = 8.87; 95% [5.92;11.84]) (See Additional File 12E).

To investigate the causes of peroxidase heterogenity, a meta-regression analysis was performed using the following variables: dose, and tested tissue. The mixed-effects models revealed that the covariates significantly not contributed to the heterogeneity observed in this analysis (R^2^ = 0.00%; Q_M_, *p* < 0.71) (See Additional File 13). A similar result was observed for GPx, where several tested variables failed to account for the heterogeneity (R^2^ = 0.00%; Q_M_, *p*< 0.55) (See Additional File 14).

Publication bias assessment was performed only for SOD, CAT, and peroxidase markers. Funnel plot analysis indicated asymmetries for all three markers: SOD (t = 4.98, *p* < 0.0001), CAT (t = 5.18, *p* < 0.0001), and peroxidase (t = 6.43, *p* < 0.0001) (See Additional File 15A – 15C).

The overall results of meta-analysis and meta regression are presented in Supplementary (see Additional File 16). This resume demonstrates that *Laportea* species exerts antinociceptive effects through coordinated anti-inflammatory and antioxidant mechanisms. Significant suppression of pro-inflammatory cytokines (IL-1β, TNF-α, IL-2, IFN-γ) and attenuation of oxidative stress markers (MDA, PCO, NO) were consistently associated with behavioral pain relief in animal models. Enhanced activity of anti-inflammatory cytokines (IL-10) and tendogenous antioxidant enzymes (GPx, GR, GST, SOD, CAT, peroxidase, and GSH) further supports the extract’s multitarget pharmacodynamic profile. Subgroup meta-regression highlights underscore the modulatory roles of extraction solvent, duration of intervention, tissue specificity, and *Laportea* species in shaping therapeutic outcomes.

## Discussion

This meta-analysis provides compelling and detailed evidence supporting the therapeutic efficacy of *Laportea* species extracts, demonstrating coordinated antinociceptive, anti-inflammatory, and antioxidant mechanisms.

*Laportea* extracts establish themselves as potent antinociceptive agents via both oral and topical routes. The oral anti-nociceptive effect is dual: a robust, unconditional reduction in writhing frequency (SMD = -6.50, *p* = 0.007) and a conditional increase in PRT (SMD = 0.95, *p* = 0.0001).

The high consistency of the writhing effect suggests a reliable peripheral mechanism, likely involving the inhibition of local inflammatory mediators, particularly prostaglandins, interleukin and TNF-α. By targeting these inflammatory mediators, which are established local nociceptive triggers, *Laportea* effectively blocks peripheral nociceptor activation [35].

In contrast, the central antinociceptive activity, indicated by a conditional increase in PRT, optimized by the tail immersion method (which is in line with the tail organ used), and methanol extraction (SMD = 10.37, respectively), yielded a far greater effect than hot plate test and ethanol extraction (SMD = 0.76, respectively). It suggests that the centrally acting components are highly sensitive to extraction polarity and pain-induction paradigms. The tail-immersion test selectively detects central antinociceptive pathways, particularly opioidergic modulation within spinal and supraspinal circuits. The specific requirement for the highly polar methanol fraction implies that the bioactive constituents responsible for these central effects possess chemical characteristics that either facilitate blood-brain barrier traversal or exhibit high affinity for specific central receptors. Related phytochemical investigations further indicate that structurally similar plant-derived compounds may also activate nitric-oxide (NO)–dependent neuromodulatory mechanisms [36, 37]. Therefore, these findings imply that while peripheral mechanisms remain robust across extraction types, central analgesic activity is extraction-dependent and model-specific, underscoring the presence of distinct multi-target bioactive constituents within the plant matrix.

Topical *Laportea* formulations also showed antinociceptive potential (SMD = -2.28, *p* < 0.001), particularly when crude extracts and transdermal patches were used (SMD = -3.71, respectively) compared to ethanol extract and cream formulation (SMD = -1.66, respectively). Crude extracts preserve a broader phytochemical profile, which may enable synergistic “entourage effects” essential for maximal nociceptive inhibiton [38]. Futhermore, transdermal patches, including lipid – based pacthes, offered enhanced bioavailability and controlled release compared to creams. This mechanism facilitates improved percutaneous penetration and ensures sustained delivery phytochemicals, thereby significantly boosting local analgesic activity [39].

Species-specific differences were also evident, with stronger antinociceptive responses in *Mus musculus* (SMD = -3.71) than *Rattus norvegicus* (SMD = -1.66), possibly due to interspecies variation in metabolic rates and nociceptive circuitry [40]. This variability underscores the need for caution in extrapolating preclinical results to the human clinical context. For the research to be successfully translated, further comparative pharmacokinetic studies are imperative to identify and account for differences in the absorption, distribution, metabolism, and excretion (ADME) of the active constituents, which may explain the observed efficacy variations between these model species.

Inflammation and oxidative stress are pivotal contributors to pain and are mechanistically intertwined in a self-perpetuating feedback loop [4, 5]. The production of pro-inflammatory cytokines, chemokines, and other mediators during inflammation, directly alters nociceptive pathways, causing hyperalgesia and allodynia [41].

Given that inflammatory mediators are central drivers of nociceptive signaling, the anti-inflammatory profile of *Laportea* provides essential mechanistic context for its antinociceptive actions. The anti-inflammatory profile of *Laportea* is clearly defined by a dual regulatory mechanism following oral administration: significant suppression of pro-inflammatory cytokines (SMD = − 2.62, *p* = 0.0002) and significant promotion of anti-inflammatory markers (SMD = 0.49, *p* = 0.043).

The findings of this meta-analysis demonstrate that *Laportea* species exert a robust systemic immunomodulatory effect through oral administration, primarily by suppressing key upstream pro-inflammatory cytokines while promoting regulatory mediators. Oral administration produced a significant overall reduction in inflammatory markers, driven by very strong decreases in IL-1β **(**SMD = -8.77) and TNF-α (SMD = -4.63), alongside moderate but significant reductions in IFN**-**γ (SMD = -1.28) and IL-2 (SMD = -0.75). These cytokines occupy important upstream positions in the inflammatory cascade, regulating macrophage activation, leukocyte recruitment, and downstream expression of COX-2 and iNOS. Therefore, the magnitude of IL-1β and TNF-α suppression suggests that *Laportea* primarily targets early intracellular signaling pathways, such as NF-κB and MAPK activation, consistent with the behavior of botanical immunomodulators reported in experimental inflammation models [42]. Heterogeneity observed only in TNF-α (I² = 84.4%) was attributable to dosage, and subgroup analysis confirmed a dose-dependent effect, with 400 mg/kg (SMD = -6.92) outperforming 200 mg/kg (SMD = -2.73). This dose-response relationship strengthens the biological plausibility of *Laportea*’s systemic action, supporting a threshold concentration needed for TNF-α modulation.

The absence of a significant reduction in edema size despite strong cytokine suppression indicates that oral *Laportea* does not sufficiently inhibit downstream effector pathways responsible for the physical manifestations of inflammation, particularly COX-2–derived prostaglandins and iNOS-derived nitric oxide. This mechanistic disconnect aligns with known inflammatory physiology, where cytokine inhibition alone may not reduce vascular leakage unless COX-2 and iNOS activities are also attenuated. This explains why edema (a late-stage, enzyme-driven outcome) did not significantly change (SMD = -2.11; *p* = 0.05) despite large cytokine changes [43]. 

In addition to suppressing pro-inflammatory markers, the extract simultaneously induced anti-inflammatory responses. There was a significant increase in anti-inflammatory markers specifically TGF-β (SMD = 0.55) and IL-10 (SMD = 0.46). The concomitant increase in these regulatory cytokines confirms that the extract acts as an immune modulator, promoting the resolution of inflammation and maintaining immune homeostasis, rather than merely inducing broad immunosuppression [44].

In the context of topical administration, while the extract provided an overall anti-inflammatory effect (SMD = -1.10, *p* < 0.043), subgroup analysis revealed a strict concentration threshold. Only the 2% topical concentration produced a significant reduction in IL-6 and paw edema. This confirms a dose-dependent threshold for topical efficacy, consistent with known challenges of transdermal phytochemical penetration, where higher concentrations are necessary to reach the dermal microenvironment and modulate local cytokine–enzyme networks [43, 45]. This finding serves as a vital practical guide for future topical formulation development, ensuring that the dose used can achieve the required therapeutic concentration at the target site.

Since inflammation, nociception, and oxidative stress are deeply interconnected biological processes, the oxidative stress findings offer essential mechanistic insight into how *Laportea* influences the upstream and downstream pathways that shape both pain and inflammatory outcomes. The collective data shows that the extract significantly reduces oxidative stress by lowering cell damage markers (MDA, NO, PCO, overall SMD = -2.42; *p* < 0.0001). Concurrently, the extract strongly upregulated endogenous antioxidant markers (SOD, CAT, GPx, GST, GSH, overal SMD = 2.77, *p* < 0.0001).

*Laportea* species extract significantly reduced oxidative stress by lowering cell damage markers, including MDA (SMD = -3.18), NO (SMD = -3.18), and PCO (SMD = -2.47). High heterogeneity was found in the NO reduction outcome (I² = 81.4%; *p* = 0.0001), which subgroup analysis showed pronounced organ specificity: *Laportea* extract significantly reduced NO levels in the liver, kidney and heart. This organ-specific targeting profile, particularly the potent reduction of NO in metabolic and excretory organs (liver and kidneys), indicates that the active metabolites such as flavonoids and phenolic acids may preferentially accumulate in, or exert their antioxidant effects upon, tissues subjected to high metabolic stress or detoxification loads. This suggests a strong potential for hepatoprotective and nephroprotective applications [46, 47]. 

The reduction of lipid peroxidation, measured by MDA levels, also exhibited high heterogeneity (I² = 69%; *p* = 0.0036), largely driven by the extraction method. The methanol extract of *Laportea* species demonstrated a significant reduction in MDA levels (SMD = -3.94), whereas the ethanol extract did not achieve a statistically significant reduction. MDA is a hallmark of oxidative damage to lipid membranes. The selectivity of the methanol extract implies that the critical antioxidant fractions responsible for scavenging reactive oxygen species and halting the chain reaction of lipid peroxidation possess characteristics that favor methanol solubility, reinforcing the requirement for a highly polar solvent to isolate lipid-protective components [48, 49]. 

*Laportea* extract significantly increased antioxidant markers: GPx (SMD = 4.38), GST (SMD = 4.08), GSH (SMD = 2.89), CAT (SMD = 2.73), SOD (SMD = 2.72), and peroxidase (SMD = 1.92). Antioxidant defense enzymes play critical roles in neutralizing ROS and protecting tissues from oxidative damage. These enzymes not only scavenge free radicals but also inhibit lipid peroxidation, maintain redox balance, and modulate intracellular signaling. Notably, NO, when reacting with superoxide, forms peroxynitrite (ONOO⁻), a cytotoxic molecule that damages proteins, lipids, and DNA, thereby exacerbating oxidative stress and inflammation [50].

Moderate to high heterogeneity was found at GPx, CAT, SOD, GSH and peroxidase (I^2^ > 50%). The mixed-effect models revealed that the covariates tested significantly contributed to the heterogeneity only in the CAT, SOD and GSH parameters.

Differences between the tested *Laportea* species are pronounced, directly impacting enzyme induction potency. *L. aestuans* exhibited a significantly stronger effect in increasing both CAT levels (SMD = 10.23) and SOD levels (SMD = 5.63), compared to *L. ovalifolia* (CAT: SMD = 1.61; SOD: SMD = 0.87). This finding establishes *L. aestuans* as the prioritized chemotype for research programs focused on enhancing enzymatic defenses against oxidative stress, likely due to a superior profile of phytochemicals that more potently induces antioxidant enzyms.

The therapeutic potential of *Laportea* species. is underpinned by a highly diverse and complex phytochemical profile, with some species, such as methanol extract of *L. bulbifera* root, contain phenolic **(**313.83 ± 4.16 mg GAE/g), condensed tannin (188.70 ± 0.43 mg GAE/g), and a phenolic acid **(**13.85 ± 0.87 mg caffeic acid equivalents (CAE)/g). Phenolic acids (e.g., chlorogenic, neochlorogenic, quercitrin) and flavonoids (e.g., quercetin derivatives, kaempferol glycosides) modulate inflammatory signaling via NF-κB, Mitogen-Activated Protein Kinase (MAPK), and NLRP3 pathways, downregulating pro-inflammatory cytokines [51–53]. These compounds also scavenge ROS through hydrogen atom donation and resonance stabilization. Flavonoids additionally promote immune balance by suppressing STAT and COX-2 activation [54, 55].

Other constituents, such as steroids and terpenoids, exert anti-inflammatory and antioxidant effects through inhibition of phospholipase A2, COX, and Lipoxygenase (LOX), and modulation of Nrf2-related antioxidant gene expression [51, 53, 56–59]. Alkaloids and lipid-based molecules like cerebrosides and ceramides further inhibit COX-2 and NO production, adding to the multi-targeted anti-inflammatory effects of *Laportea* species [60–63]. These bioactive compounds act synergistically to modulate cytokine production, stabilize cellular environments, regulate redox balance, and alleviate pain by reducing nociceptor hyperexcitability, making *Laportea* species. a promising natural remedy for managing inflammation, pain, and oxidative stress-related conditions [64].

The difference in antioxidant effects is also influenced by the duration of intervention. We group them into CAT– acute response, SOD – adaptive response, dan GSH – chronic response.

Catalase activity showed the highest increase after short treatment durations of 1–3 days (SMD = 18.54), with efficacy decreasing markedly for longer durations (4–7 days: SMD = 3.55; >7 days: SMD = 2.15). Catalase functions to rapidly detoxify hydrogen peroxide generated by SOD activity. This acute, high-magnitude response is characteristic of an immediate defense mechanism triggered by the presence of initial oxidative challenge or the extract itself, quickly restoring cellular redox balance [65]. 

In contrast, SOD levels showed no significant increase during short durations (1–3 days). Maximal increase occurred during the intermediate period of 4–7 days (SMD = 9.31), which was significantly stronger than treatment beyond 7 days (SMD = 1.30). SOD converts the highly damaging superoxide radical into hidrogen peroxide. The requirement for a longer duration suggests that the induction of SOD requires transcriptional up-regulation or synthesis of new enzyme protein, indicative of a sustained, adaptive response to protect against chronic oxidative precursor generation [65]. 

The GSH system required the longest treatment duration, with the highest increases observed after treatments exceeding seven days (SMD = 8.87). This delay reinforces that the constituents responsible for synthesizing or regulating GSH are distinct semi-polar compounds found in the ethanol fraction, and their action involves chronic maintenance mechanisms separate from the acute, primary enzyme induction pathways [66].

The meta-regression for SOD and CAT revealed distinct kinetic profiles related to treatment duration, suggesting an adaptive cellular defense strategy consistent with phytochemical-mediated Nrf2–ARE activation.

These temporal patterns of enzyme induction are consistent with well-established mechanisms of phytochemical-mediated activation of the Keap1–Nrf2–ARE pathway, which regulates transcription of antioxidant enzymes in response to oxidative or xenobiotic stimuli. Many plant-derived polyphenols, including flavonoids and lignans, have been shown to activate Nrf2, promoting the expression of SOD, CAT, GPx, and glutathione-related enzymes, providing a mechanistic basis for the enzyme-enhancing effects observed with *Laportea* extracts [67]. 

The regulation of SOD, CAT and GSH presented a unique solvent dependency. While methanol extract was overwhelmingly superior for inducing SOD, CAT, and reducing MDA, the ethanol extract exhibited the strongest effect on increasing GSH levels, while the methanol extract showed no significant effect. This divergence reflects fundamental biochemical differences in how GSH is regulated. GSH synthesis requires transcriptional activation of γ-glutamylcysteine synthetase and other Nrf2-dependent genes, a process that typically occurs more slowly than rapid enzyme induction seen in SOD and CAT pathways. Moreover, semi-polar phytochemicals—such as conjugated flavonoids and phenolic acids—are more efficiently extracted with ethanol and are well-known activators of the Nrf2–ARE axis, which enhances GSH biosynthesis during chronic oxidative challenge [68, 69]. 

The current meta-analysis provides the most robust scientific evidence to date that the antinociceptive effect of *Laportea* species is mediated by a coordinated multi-target mechanism, supporting its use as a polypharmacological agent rather than a single-compound therapy. The convergence of therapeutic benefits across three distinct physiological pathways—antinociceptive, anti-inflammatory, and antioxidant—demonstrates its strong scientific potential for managing chronic inflammatory pain. Given that chronic pain is virtually always accompanied by inflammatory and oxidative stress components, this multi-target approach offers a critical advantage over conventional single-target drugs, which often provide only palliative effects at a single node of the pathological pathway.

*Laportea*’s ability to simultaneously suppress key pro-inflammatory mediators (IL-1β, TNF-α) while promoting resolution mediators (IL-10, TGF-β) provides an intervention designed to restore cellular homeostasis rather than merely suppress symptoms. Furthermore, the finding of tissue specificity in NO reduction suggests a potential for development as an organ-protective agent (hepatoprotective and nephroprotective) in clinical settings characterized by high metabolic stress, broadening its therapeutic scope.

The in-depth analysis of methodological sensitivity serves as a framework for formulation standardization, a prerequisite step for successful translation into clinical studies. These findings convert experimental variables into non-negotiable formulation requirements. It must be established that the extraction method must be selected based on the specific therapeutic goal. For acute effects (such as lipid peroxidation reduction and central analgesia), the methanol-extracted fraction should be prioritized. Conversely, for chronic maintenance and long-term systemic benefits (such as GSH enhancement), the ethanol-extracted fraction is required.

The clear difference in antioxidant enzyme induction potency between *L. aestuans* and *L. ovalifolia* mandates the selection of the highest-potency chemotype (*L. aestuans*) for future research and production to ensure maximum efficacy.The confirmation of dose-dependent effects for TNF-α (systemic) and IL-6 / edema (topical) demands that formulations guarantee concentrations above the identified efficacy thresholds for both oral and transdermal routes of administration. Future in vivo studies should focus on definitively linking specific isolated phytochemical compounds (such as those identified in *L. bulbifera* or *L. aestuans*) to the activation of key anti-inflammatory (NF-κB, MAPK) and antioxidant (Nrf2/ARE) pathways, further validating the observed polypharmacological mechanism.

## Limitations

Language restriction: it must be recognized that limiting the language to English and Indonesian can exclude relevant research published in other languages (e.g., languages used in endemic regions of Africa or China). This limitation potentially leads to language bias, which could affect the overall effect estimate if the excluded studies reported differing or null results.

The primary limitation of this review stems from the poor methodological reporting in the primary preclinical studies, rather than the absence of efficacy. This ‘unclear risk’ is not necessarily an indication of a true bias but highlights a critical reporting bias that obscures a clear judgment of the study’s internal validity.” Consequently, the certainty of evidence for the efficacy of *Laportea* species is reduced. To facilitate accurate translation of preclinical findings to clinical practice, we strongly recommend that future researchers evaluating *Laportea* strictly adhere to established reporting guidelines, such as the ARRIVE Guidelines (Animal Research: Reporting of In Vivo Experiments and the Syrcle/Camarades criteria. Explicit reporting of methods for randomization, allocation concealment, and blinding is paramount for enhancing methodological rigor and the overall quality of evidence in this field.

Unmeasured heterogeneity: the inability of meta-regression to fully explain the heterogeneity for all outcomes points to the presence of critical unmeasured confounders, such as the exact preparation or galenic properties, beyond the basic extraction types and plant parts reported.

Publication bias: the high statistical heterogeneity (e.g., I^2^ for PRT, SOD, and CAT) and the significant findings from Egger’s test for multiple outcomes (including writhing and antioxidant markers) indicate evidence of publication bias (small study effects). This suggests that the large SMDs reported in smaller studies might be inflated, necessitating a cautious interpretation of the absolute effect magnitude.

## Conclusion

This meta-analysis demonstrates that extract of *Laportea* species possesses significant anti-inflammatory, antioxidant, and antinociceptive properties through multi-targeted mechanisms. The results provide definitive quantitative evidence that methodological precision—in terms of solvent polarity, concentration, and duration—is not merely an experimental variable but a necessary condition for eliciting the desired mechanistic pathway activation.

The extract effectively downregulates key pro-inflammatory cytokines (IL-2, IL-1β, IFN-γ, TNF-α, IL-6) reduces oxidative stress markers (MDA, PCO, NO), enhances anti inflammatory cytokines (IL-10 and TGF-β) and endogenous antioxidant defenses (SOD, CAT, GPx, GR, GST, GSH, and Peroxidase). These effects collectively contribute to reduced nociceptor sensitization and pain perception. The pharmacological efficacy is influenced by extraction methods, treatment duration, route of administration, tissue specifity, and species variation, underscoring the need for standardization in future studies. The diverse phytochemical constituents—including flavonoids, phenolic acids, alkaloids, and terpenoids—interact synergistically to modulate inflammatory and oxidative pathways. Altogether, *Laportea* species holds strong therapeutic promise as a natural agent for managing inflammation, oxidative stress, and pain. Further investigations should focus on bioavailability, pharmacokinetics, and optimized formulations, to enable translational applications in clinical settings.

## Supplementary Information

Supplementary Material 1.

Supplementary Material 2.

Supplementary Material 3.

Supplementary Material 4.

Supplementary Material 5.

Supplementary Material 6.

Supplementary Material 7.

Supplementary Material 8.

Supplementary Material 9.

Supplementary Material 10.

Supplementary Material 11.

Supplementary Material 12.

Supplementary Material 13.

Supplementary Material 14.

Supplementary Material 15.

Supplementary Material 16.

## Data Availability

All data generated or analyzed during this study are included in this published article and its additional files.

## Abbreviations

CAT: Catalase
CFA: Complete Freund Adjuvan
COX: Cyclooxygenase
GSH: Glutathione
GPx: Glutathione Peroxidase
GR: Glutathione Reductase
GST: Glutathione transferase
IFN-γ: Interferon gamma
IL: Interleukin
iNOS: inducible Nitrit Oxide Synthase
LOX: Lipoxygenase
MAPK: Mitogen-Activated Protein Kinase
MDA: Malondialdehyde
NF-kB: Nuclear factor kappa-light-chain-enhancer of activated B cells
NO: Nitrit Oxide
Nrf-2: Nuclear factor erythroid 2-related factor
PCO: Protein Carbonylation
PO: Peroxidase
PRT: Pain Reaction Time
SOD: Superoxida dismutase
TC: Total Coumarin
TGF-β: Transforming Growth Factor – Beta
TNF-α: Tumour Necrosis Factor alfa
