# Supplementary material for: Antinociceptive activity of Laportea species mediated by anti-inflammatory and antioxidant mechanisms: a systematic review and meta-analysis of in vivo animal studies
Source: BMC Complement Med Ther. 2026 Feb 3;26:85. doi: 10.1186/s12906-026-05262-0 (PMC12958739; doi:10.1186/s12906-026-05262-0)
Supplement: Supplementary file 1 — Supplementary Material 1. [file 12906_2026_5262_MOESM1_ESM.pdf]

ADDITIONAL FILE 1

Literature Search Strategies

Database	Literature search strategies
1. Pubmed	(Laportea AND (anti inflammatory or anti inflammation or analgesic or animal or rat or anti oxidant or mice or in vivo)) AND (Laportea [Title/Abstract])
2. Science-Direct:	(laportea) AND (anti inflammation OR analgesic OR antioxidant OR anti inflammatory OR animal OR in vivo OR rat OR mice)
3. Scopus	(TITLE-ABS-KEY (laportea) AND TITLE-ABS-KEY (analgesic or analgesia or anti inflammatory or anti inflammation or anti oxidant or in vivo or mice or rat))
4. EBSCO	(laportea) AND (anti inflammation OR analgesic OR antioxidant OR anti inflammatory OR animal OR in vivo OR rat OR mice)
5. Google scholar	laportea AND (inflammation OR inflammatory OR analgesic OR antioxidant OR vivo OR animal OR rat OR mice)
