# Supplementary material for: Antinociceptive activity of Laportea species mediated by anti-inflammatory and antioxidant mechanisms: a systematic review and meta-analysis of in vivo animal studies
Source: BMC Complement Med Ther. 2026 Feb 3;26:85. doi: 10.1186/s12906-026-05262-0 (PMC12958739; doi:10.1186/s12906-026-05262-0)
Supplement: Supplementary file 2 — Supplementary Material 2. [file 12906_2026_5262_MOESM2_ESM.pdf]

**ADDITIONAL FILE 2**

**ANALGESIC OUTCOME: FREQUENCY OF WRITHING**

**A. FUNNEL PLOT**

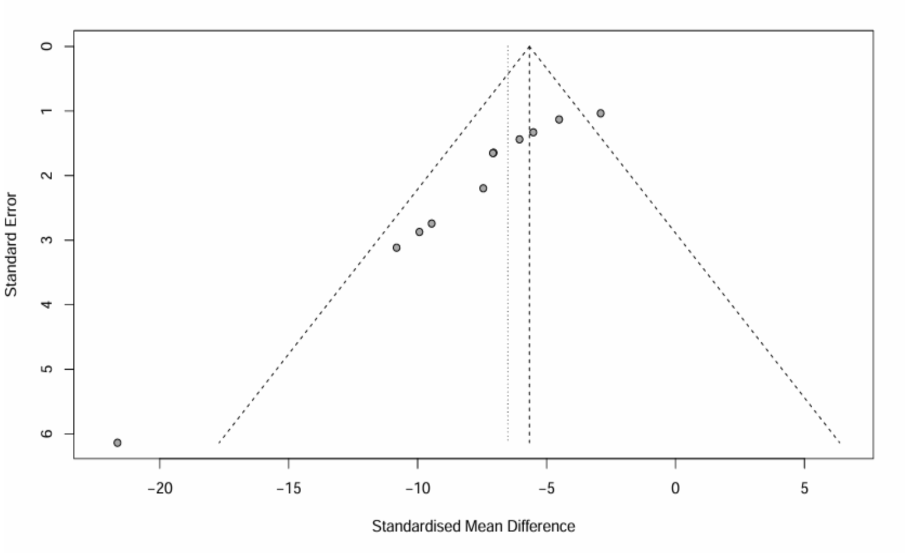


**B. EGGER'S TEST**

Test result: t = -9.30, df = 9, p-value < 0.0001

Bias estimate: -3.5554 (SE = 0.3825)

**C. Meta Reggresion**

Mixed-effects model (k= 11)

$R^2$ = 40,06 %; p = 0,20

| Variabel | β | SMD [95% CI] | p-value |
|---|---|---|---|
| laportea_sp | 3.18 | -1.99 [-8,23; 4,26] | 0,53 |
| extract | 1.93 | -3.43 [-7,20; 0,35] | 0,08 |
| dose | 1.10 | -0.39 [-2,57; 1,78] | 0,72 |
| method | 1.82 | -0.93 [-4,51; 2,64] | 0.55 |
| duration | 1.70 | -1.02 [-4,36; 2,33] | 0,55 |
