# Supplementary material for: Antinociceptive activity of Laportea species mediated by anti-inflammatory and antioxidant mechanisms: a systematic review and meta-analysis of in vivo animal studies
Source: BMC Complement Med Ther. 2026 Feb 3;26:85. doi: 10.1186/s12906-026-05262-0 (PMC12958739; doi:10.1186/s12906-026-05262-0)
Supplement: Supplementary file 3 — Supplementary Material 3. [file 12906_2026_5262_MOESM3_ESM.pdf]

ADDITIONAL FILE 3

ANALGESIC OUTCOME: PAIN REACTION TIME (PRT)

A. Funnel Plot

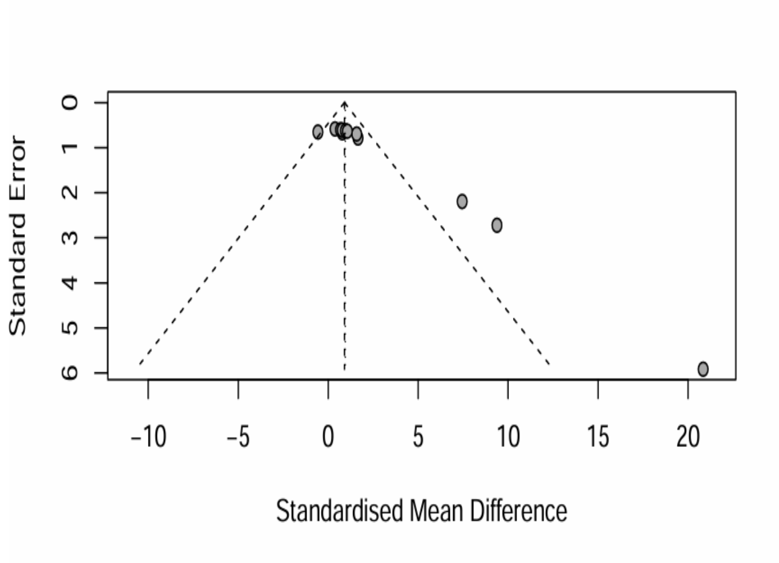

B. Egger's Test

Test result: t = 7.26, df = 10, p-value < 0.0001

Bias estimate: 4.0863 (SE = 0.5632)

C. Meta Regression

Mixed-effects model (k= 12)

R^2= 100%; β = 1.36; Q_M, p < 0.0001

Variabel	β	SMD [95% CI]	p value
dose	0,42	0,83 [-0.09; 1,69]	0,08
method	1.36	8.88 [5,58; 12,13]	<0,0001
animal species	0.49	0,05 [-0,91; 1,01]	0,88
extract	1.36	8.86 [6,16; 11,74]	<0,0001
laportea species	0,25	0,03 [-0,45; 0.50]	0,92
tissue	1.36	8.86 [6,22; 11,56]	<.00001

D. Subgroup: Extraction Type

Study or Subgroup	Experimental Mean	Experimental SD	Experimental Total	Control Mean	Control SD	Control Total	Weight (common)	Weight (random)	Std. Mean Difference IV, Fixed + Random, 95% CI
**extract = 1**									
Ashidi, 2017 (1)	0.81	0.0600	5	0.96	0.3300	5	10.6%	10.7%	−0.57 [−1.85; 0.71]
Ashidi, 2017 (2)	1.19	0.1800	5	0.96	0.3300	5	10.1%	10.3%	0.78 [−0.53; 2.09]
La Basy, 2024 (1)	16.10	37.5900	6	4.03	17.7800	6	13.2%	12.7%	0.38 [−0.77; 1.52]
La Basy, 2024 (4)	52.15	37.9900	6	1.01	90.2800	6	12.5%	12.1%	0.68 [−0.50; 1.86]
Ashidi, 2017 (3)	1.53	0.2900	5	0.96	0.3300	5	7.3%	7.9%	1.66 [0.12; 3.20]
La Basy, 2024 (2)	43.50	63.3100	6	4.03	17.7800	6	12.2%	11.9%	0.78 [−0.41; 1.98]
La Basy, 2024 (3)	72.50	53.5200	6	4.03	17.7800	6	9.3%	9.6%	1.58 [0.22; 2.95]
La Basy, 2024 (5)	68.27	15.8400	6	1.01	90.2800	6	11.7%	11.5%	0.96 [−0.26; 2.18]
La Basy, 2024 (6)	75.38	12.0500	6	1.01	90.2800	6	11.3%	11.2%	1.07 [−0.18; 2.31]
**Total (common effect, 95% CI)**			**51**			**51**	**98.3%**	**.**	**0.76 [0.34; 1.18]**
**Total (random effect, 95% CI)**							**.**	**97.9%**	**0.76 [0.34; 1.18]**
Heterogeneity: Tau^2 = < 0.0001; Chi^2 = 7.64, df = 8 (P = 0.4698); I^2 = 0%									
**extract = 2**									
Islam, 2014 (4)	2.90	0.1800	5	1.15	0.2400	5	0.9%	1.2%	7.45 [3.15; 11.75]
Islam, 2014 (5)	3.86	0.2800	5	1.15	0.2400	5	0.6%	0.8%	9.38 [4.05; 14.72]
Islam, 2014 (6)	5.28	0.0800	5	1.15	0.2400	5	0.1%	0.2%	20.84 [9.25; 32.43]
**Total (common effect, 95% CI)**			**15**			**15**	**1.7%**	**.**	**9.18 [5.97; 12.40]**
**Total (random effect, 95% CI)**							**.**	**2.1%**	**10.37 [4.95; 15.78]**
Heterogeneity: Tau^2 = 12.1781; Chi^2 = 4.52, df = 2 (P = 0.1045); I^2 = 55.7%									
**Total (common effect, 95% CI)**			**66**			**66**	**100.0%**	**.**	**0.90 [0.49; 1.32]**
**Total (random effect, 95% CI)**							**.**	**100.0%**	**0.95 [0.48; 1.42]**
**Prediction interval**									**[0.04; 1.86]**

Heterogeneity: Tau^2 = 0.1141; Chi^2 = 38.02, df = 11 (P < 0.0001); I^2 = 71.1%
Test for subgroup differences (common effect): Chi^2 = 25.87, df = 1 (P < 0.0001)
Test for subgroup differences (random effects): Chi^2 = 12.02, df = 1 (P = 0.0005)

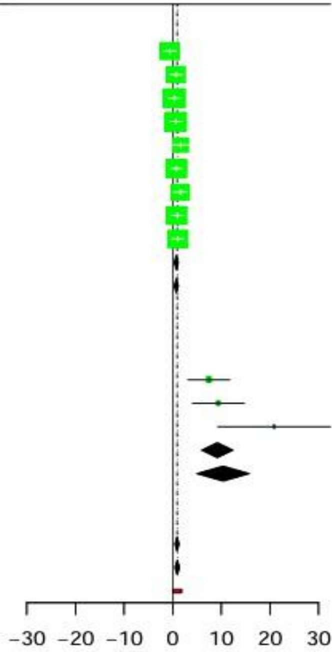

Extract 1: Ethanol
Extract 2: Methanol

E. Subgroup Methodology

Study or Subgroup	Experimental Mean	Experimental SD	Experimental Total	Control Mean	Control SD	Control Total	Weight (common)	Weight (random)	Std. Mean Difference IV, Fixed + Random, 95% CI
**method = 1**									
Ashidi, 2017 (1)	0.81	0.0600	5	0.96	0.3300	5	10.6%	10.7%	-0.57 [-1.85; 0.71]
Ashidi, 2017 (2)	1.19	0.1800	5	0.96	0.3300	5	10.1%	10.3%	0.78 [-0.53; 2.09]
La Basy, 2024 (1)	16.10	37.5900	6	4.03	17.7800	6	13.2%	12.7%	0.38 [-0.77; 1.52]
La Basy, 2024 (4)	52.15	37.9900	6	1.01	90.2800	6	12.5%	12.1%	0.68 [-0.50; 1.86]
Ashidi, 2017 (3)	1.53	0.2900	5	0.96	0.3300	5	7.3%	7.9%	1.66 [0.12; 3.20]
La Basy, 2024 (2)	43.50	63.3100	6	4.03	17.7800	6	12.2%	11.9%	0.78 [-0.41; 1.98]
La Basy, 2024 (3)	72.50	53.5200	6	4.03	17.7800	6	9.3%	9.6%	1.58 [0.22; 2.95]
La Basy, 2024 (5)	68.27	15.8400	6	1.01	90.2800	6	11.7%	11.5%	0.96 [-0.26; 2.18]
La Basy, 2024 (6)	75.38	12.0500	6	1.01	90.2800	6	11.3%	11.2%	1.07 [-0.18; 2.31]
**Total (common effect, 95% CI)**			**51**			**51**	**98.3%**	.	**0.76 [0.34; 1.18]**
**Total (random effect, 95% CI)**							.	**97.9%**	**0.76 [0.34; 1.18]**
Heterogeneity: Tau^2 = < 0.0001; Chi^2 = 7.64, df = 8 (P = 0.4698); I^2 = 0%									
**method = 2**									
Islam, 2014 (4)	2.90	0.1800	5	1.15	0.2400	5	0.9%	1.2%	7.45 [3.15; 11.75]
Islam, 2014 (5)	3.86	0.2800	5	1.15	0.2400	5	0.6%	0.8%	9.38 [4.05; 14.72]
Islam, 2014 (6)	5.28	0.0800	5	1.15	0.2400	5	0.1%	0.2%	20.84 [9.25; 32.43]
**Total (common effect, 95% CI)**			**15**			**15**	**1.7%**	.	**9.18 [5.97; 12.40]**
**Total (random effect, 95% CI)**							.	**2.1%**	**10.37 [4.95; 15.78]**
Heterogeneity: Tau^2 = 12.1781; Chi^2 = 4.52, df = 2 (P = 0.1045); I^2 = 55.7%									
**Total (common effect, 95% CI)**			**66**			**66**	**100.0%**	.	**0.90 [0.49; 1.32]**
**Total (random effect, 95% CI)**							.	**100.0%**	**0.95 [0.48; 1.42]**
**Prediction interval**									**[0.04; 1.86]**

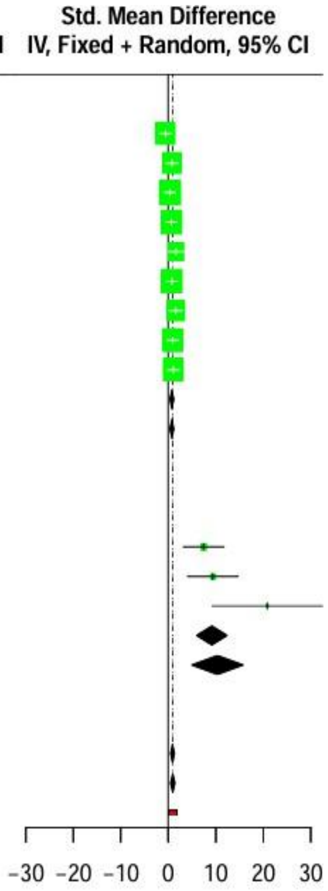

Heterogeneity: Tau^2 = 0.1141; Chi^2 = 38.02, df = 11 (P < 0.0001); I^2 = 71.1%

Test for subgroup differences (common effect): Chi^2 = 25.87, df = 1 (P < 0.0001)

Test for subgroup differences (random effects): Chi^2 = 12.02, df = 1 (P = 0.0005)

Method 1: Hot plate test

Method 2: Tail Immersion Test

F. Subgroup: Tissue used

Study or Subgroup	Experimental Mean	Experimental SD	Experimental Total	Control Mean	Control SD	Control Total	Weight (common)	Weight (random)	Std. Mean Difference IV, Fixed + Random, 95% CI
**tissue = 1**									
Ashidi, 2017 (1)	0.81	0.0600	5	0.96	0.3300	5	10.6%	10.7%	−0.57 [−1.85; 0.71]
Ashidi, 2017 (2)	1.19	0.1800	5	0.96	0.3300	5	10.1%	10.3%	0.78 [−0.53; 2.09]
La Basy, 2024 (1)	16.10	37.5900	6	4.03	17.7800	6	13.2%	12.7%	0.38 [−0.77; 1.52]
La Basy, 2024 (4)	52.15	37.9900	6	1.01	90.2800	6	12.5%	12.1%	0.68 [−0.50; 1.86]
Ashidi, 2017 (3)	1.53	0.2900	5	0.96	0.3300	5	7.3%	7.9%	1.66 [0.12; 3.20]
La Basy, 2024 (2)	43.50	63.3100	6	4.03	17.7800	6	12.2%	11.9%	0.78 [−0.41; 1.98]
La Basy, 2024 (3)	72.50	53.5200	6	4.03	17.7800	6	9.3%	9.6%	1.58 [0.22; 2.95]
La Basy, 2024 (5)	68.27	15.8400	6	1.01	90.2800	6	11.7%	11.5%	0.96 [−0.26; 2.18]
La Basy, 2024 (6)	75.38	12.0500	6	1.01	90.2800	6	11.3%	11.2%	1.07 [−0.18; 2.31]
**Total (common effect, 95% CI)**			**51**			**51**	**98.3%**	**.**	**0.76 [0.34; 1.18]**
**Total (random effect, 95% CI)**							**.**	**97.9%**	**0.76 [0.34; 1.18]**
Heterogeneity: Tau^2 = < 0.0001; Chi^2 = 7.64, df = 8 (P = 0.4698); I^2 = 0%									
**tissue = 2**									
Islam, 2014 (4)	2.90	0.1800	5	1.15	0.2400	5	0.9%	1.2%	7.45 [3.15; 11.75]
Islam, 2014 (5)	3.86	0.2800	5	1.15	0.2400	5	0.6%	0.8%	9.38 [4.05; 14.72]
Islam, 2014 (6)	5.28	0.0800	5	1.15	0.2400	5	0.1%	0.2%	20.84 [9.25; 32.43]
**Total (common effect, 95% CI)**			**15**			**15**	**1.7%**	**.**	**9.18 [5.97; 12.40]**
**Total (random effect, 95% CI)**							**.**	**2.1%**	**10.37 [4.95; 15.78]**
Heterogeneity: Tau^2 = 12.1781; Chi^2 = 4.52, df = 2 (P = 0.1045); I^2 = 55.7%									
**Total (common effect, 95% CI)**			**66**			**66**	**100.0%**	**.**	**0.90 [0.49; 1.32]**
**Total (random effect, 95% CI)**							**.**	**100.0%**	**0.95 [0.48; 1.42]**
**Prediction interval**									**[0.04; 1.86]**

Heterogeneity: Tau^2 = 0.1141; Chi^2 = 38.02, df = 11 (P < 0.0001); I^2 = 71.1%
Test for subgroup differences (common effect): Chi^2 = 25.87, df = 1 (P < 0.0001)
Test for subgroup differences (random effects): Chi^2 = 12.02, df = 1 (P = 0.0005)

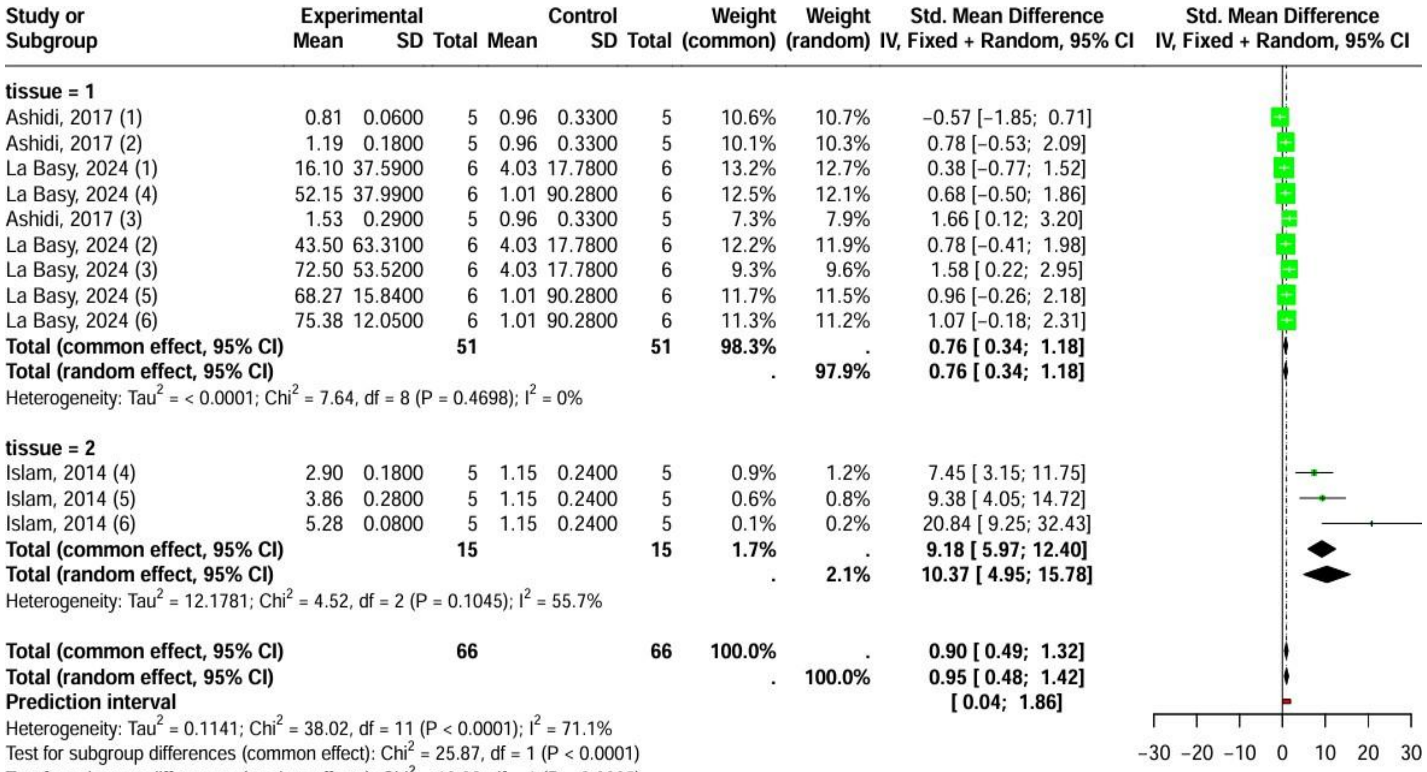

Tissue 1: Leg
Tissue 2: Tail
