# Supplementary material for: Antinociceptive activity of Laportea species mediated by anti-inflammatory and antioxidant mechanisms: a systematic review and meta-analysis of in vivo animal studies
Source: BMC Complement Med Ther. 2026 Feb 3;26:85. doi: 10.1186/s12906-026-05262-0 (PMC12958739; doi:10.1186/s12906-026-05262-0)
Supplement: Supplementary file 4 — Supplementary Material 4. [file 12906_2026_5262_MOESM4_ESM.pdf]

**ADDITIONAL FILE 4**

**ANALGESIC TOPICAL**

## A. Meta Regression

Mixed-effect model (k = 9)
$R^2$= 98.89 %; $Q_M$, p = 0.0009

| Variabel | β | SMD [95% CI] | p-value |
|---|---|---|---|
| dose | 0.42 | -0.82 [-16.32; -0.001] | 0.05 |
| extract | 0.88 | -25.92 [-43.11; -0.87] | 0.003 |
| duration | 0.69 | 0.97 [-0.39; 23.26] | 0.16 |
| animal_sp | 17.18 | -36.11 [-69.78; -0.25] | 0.04 |
| method | 0.94 | 0.93 [-0.92; 27.77] | 0.32 |
| Topical form | 10.28 | -21.48 [-41.63; -0.13] | 0.04 |

## B. Subgroup: Extraction Type

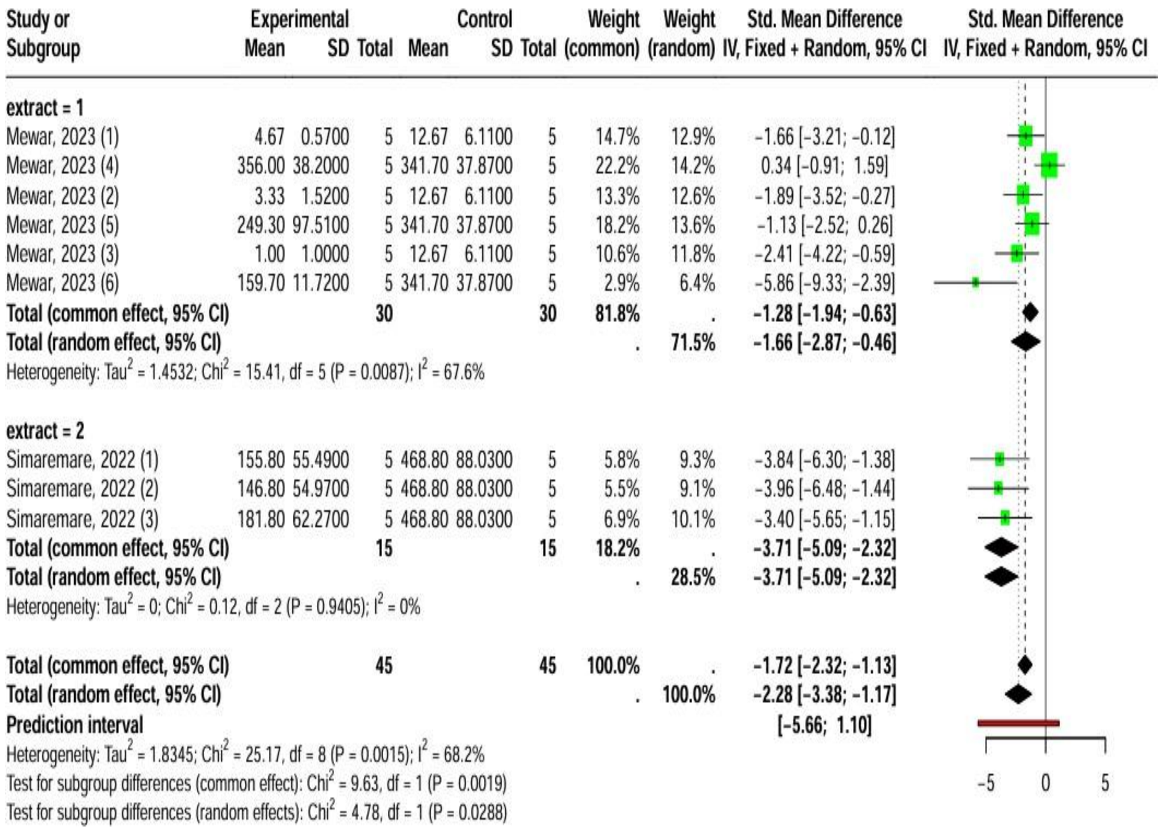


Extract 1: Ethanol
Extract 2: Crude

**C. Subgroup: Topical Form**

| Study or Subgroup | Experimental Mean | SD | Total | Control Mean | SD | Total | Weight (common) | Weight (random) | Std. Mean Difference IV, Fixed + Random, 95% CI |
|---|---|---|---|---|---|---|---|---|---|
| **form = 1** | | | | | | | | | |
| Mewar, 2023 (1) | 4.67 | 0.5700 | 5 | 12.67 | 6.1100 | 5 | 14.7% | 12.9% | −1.66 [−3.21; −0.12] |
| Mewar, 2023 (4) | 356.00 | 38.2000 | 5 | 341.70 | 37.8700 | 5 | 22.2% | 14.2% | 0.34 [−0.91; 1.59] |
| Mewar, 2023 (2) | 3.33 | 1.5200 | 5 | 12.67 | 6.1100 | 5 | 13.3% | 12.6% | −1.89 [−3.52; −0.27] |
| Mewar, 2023 (5) | 249.30 | 97.5100 | 5 | 341.70 | 37.8700 | 5 | 18.2% | 13.6% | −1.13 [−2.52; 0.26] |
| Mewar, 2023 (3) | 1.00 | 1.0000 | 5 | 12.67 | 6.1100 | 5 | 10.6% | 11.8% | −2.41 [−4.22; −0.59] |
| Mewar, 2023 (6) | 159.70 | 11.7200 | 5 | 341.70 | 37.8700 | 5 | 2.9% | 6.4% | −5.86 [−9.33; −2.39] |
| **Total (common effect, 95% CI)** | | | **30** | | | **30** | **81.8%** | . | **−1.28 [−1.94; −0.63]** |
| **Total (random effect, 95% CI)** | | | | | | | . | **71.5%** | **−1.66 [−2.87; −0.46]** |
| Heterogeneity: $Tau^2 = 1.4532$; $Chi^2 = 15.41$, df = 5 (P = 0.0087); $I^2 = 67.6\%$ | | | | | | | | | |
| **form = 2** | | | | | | | | | |
| Simaremare, 2022 (1) | 155.80 | 55.4900 | 5 | 468.80 | 88.0300 | 5 | 5.8% | 9.3% | −3.84 [−6.30; −1.38] |
| Simaremare, 2022 (2) | 146.80 | 54.9700 | 5 | 468.80 | 88.0300 | 5 | 5.5% | 9.1% | −3.96 [−6.48; −1.44] |
| Simaremare, 2022 (3) | 181.80 | 62.2700 | 5 | 468.80 | 88.0300 | 5 | 6.9% | 10.1% | −3.40 [−5.65; −1.15] |
| **Total (common effect, 95% CI)** | | | **15** | | | **15** | **18.2%** | . | **−3.71 [−5.09; −2.32]** |
| **Total (random effect, 95% CI)** | | | | | | | . | **28.5%** | **−3.71 [−5.09; −2.32]** |
| Heterogeneity: $Tau^2 = 0$; $Chi^2 = 0.12$, df = 2 (P = 0.9405); $I^2 = 0\%$ | | | | | | | | | |
| **Total (common effect, 95% CI)** | | | **45** | | | **45** | **100.0%** | . | **−1.72 [−2.32; −1.13]** |
| **Total (random effect, 95% CI)** | | | | | | | . | **100.0%** | **−2.28 [−3.38; −1.17]** |
| **Prediction interval** | | | | | | | | | **[−5.66; 1.10]** |
| Heterogeneity: $Tau^2 = 1.8345$; $Chi^2 = 25.17$, df = 8 (P = 0.0015); $I^2 = 68.2\%$ | | | | | | | | | |
| Test for subgroup differences (common effect): $Chi^2 = 9.63$, df = 1 (P = 0.0019) | | | | | | | | | |
| Test for subgroup differences (random effects): $Chi^2 = 4.78$, df = 1 (P = 0.0288) | | | | | | | | | |

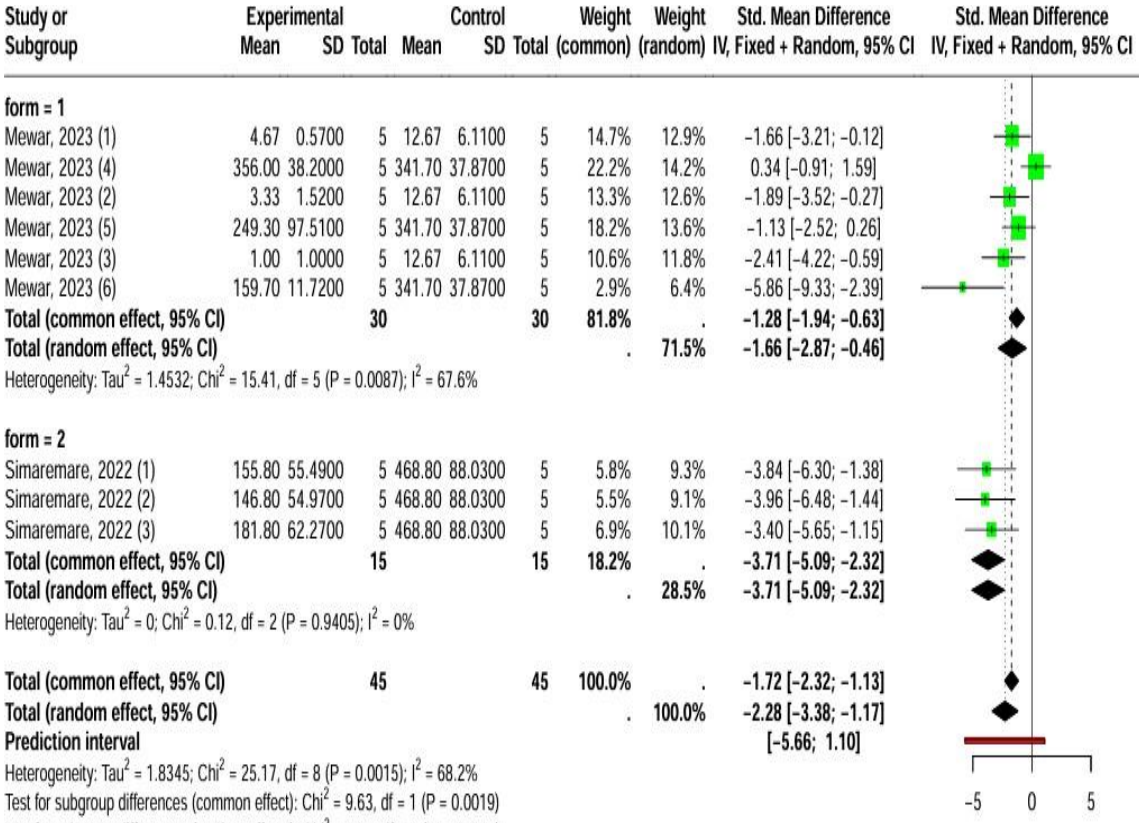


Form 1: Cream
Form 2: Patch

**D. Subgroup: Animal Species**

| Study or Subgroup | Experimental Mean | Experimental SD | Experimental Total | Control Mean | Control SD | Control Total | Weight (common) | Weight (random) | Std. Mean Difference IV, Fixed + Random, 95% CI |
|---|---|---|---|---|---|---|---|---|---|
| **animal_sp = 1** | | | | | | | | | |
| Mewar, 2023 (1) | 4.67 | 0.5700 | 5 | 12.67 | 6.1100 | 5 | 14.7% | 12.9% | −1.66 [−3.21; −0.12] |
| Mewar, 2023 (4) | 356.00 | 38.2000 | 5 | 341.70 | 37.8700 | 5 | 22.2% | 14.2% | 0.34 [−0.91; 1.59] |
| Mewar, 2023 (2) | 3.33 | 1.5200 | 5 | 12.67 | 6.1100 | 5 | 13.3% | 12.6% | −1.89 [−3.52; −0.27] |
| Mewar, 2023 (5) | 249.30 | 97.5100 | 5 | 341.70 | 37.8700 | 5 | 18.2% | 13.6% | −1.13 [−2.52; 0.26] |
| Mewar, 2023 (3) | 1.00 | 1.0000 | 5 | 12.67 | 6.1100 | 5 | 10.6% | 11.8% | −2.41 [−4.22; −0.59] |
| Mewar, 2023 (6) | 159.70 | 11.7200 | 5 | 341.70 | 37.8700 | 5 | 2.9% | 6.4% | −5.86 [−9.33; −2.39] |
| **Total (common effect, 95% CI)** | | | **30** | | | **30** | **81.8%** | . | **−1.28 [−1.94; −0.63]** |
| **Total (random effect, 95% CI)** | | | | | | | . | **71.5%** | **−1.66 [−2.87; −0.46]** |
| Heterogeneity: $Tau^2 = 1.4532$; $Chi^2 = 15.41$, df = 5 (P = 0.0087); $I^2 = 67.6\%$ | | | | | | | | | |
| **animal_sp = 2** | | | | | | | | | |
| Simaremare, 2022 (1) | 155.80 | 55.4900 | 5 | 468.80 | 88.0300 | 5 | 5.8% | 9.3% | −3.84 [−6.30; −1.38] |
| Simaremare, 2022 (2) | 146.80 | 54.9700 | 5 | 468.80 | 88.0300 | 5 | 5.5% | 9.1% | −3.96 [−6.48; −1.44] |
| Simaremare, 2022 (3) | 181.80 | 62.2700 | 5 | 468.80 | 88.0300 | 5 | 6.9% | 10.1% | −3.40 [−5.65; −1.15] |
| **Total (common effect, 95% CI)** | | | **15** | | | **15** | **18.2%** | . | **−3.71 [−5.09; −2.32]** |
| **Total (random effect, 95% CI)** | | | | | | | . | **28.5%** | **−3.71 [−5.09; −2.32]** |
| Heterogeneity: $Tau^2 = 0$; $Chi^2 = 0.12$, df = 2 (P = 0.9405); $I^2 = 0\%$ | | | | | | | | | |
| **Total (common effect, 95% CI)** | | | **45** | | | **45** | **100.0%** | . | **−1.72 [−2.32; −1.13]** |
| **Total (random effect, 95% CI)** | | | | | | | . | **100.0%** | **−2.28 [−3.38; −1.17]** |
| **Prediction interval** | | | | | | | | | **[−5.66; 1.10]** |

Heterogeneity: $Tau^2 = 1.8345$; $Chi^2 = 25.17$, df = 8 (P = 0.0015); $I^2 = 68.2\%$
Test for subgroup differences (common effect): $Chi^2 = 9.63$, df = 1 (P = 0.0019)
Test for subgroup differences (random effects): $Chi^2 = 4.78$, df = 1 (P = 0.0288)

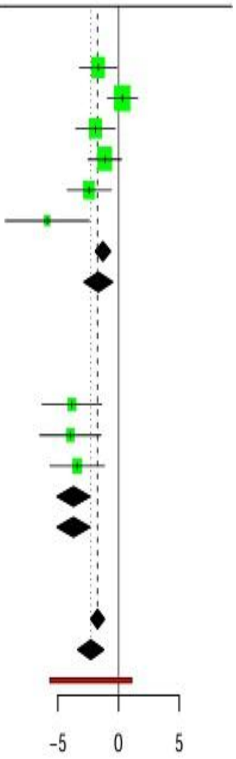


Animal species 1: Rat
Animal species 2: Mice
