# Supplementary material for: Antinociceptive activity of Laportea species mediated by anti-inflammatory and antioxidant mechanisms: a systematic review and meta-analysis of in vivo animal studies
Source: BMC Complement Med Ther. 2026 Feb 3;26:85. doi: 10.1186/s12906-026-05262-0 (PMC12958739; doi:10.1186/s12906-026-05262-0)
Supplement: Supplementary file 5 — Supplementary Material 5. [file 12906_2026_5262_MOESM5_ESM.pdf]

# ADDITIONAL FILE 5

## Oral Pro Inflammatory Markers

### TNF-α level (Subgroup: Dose)

| Study or Subgroup | Experimental Mean | Experimental SD | Experimental Total | Control Mean | Control SD | Control Total | Weight (common) | Weight (random) | Std. Mean Difference IV, Fixed + Random, 95% CI |
|---|---|---|---|---|---|---|---|---|---|
| **dose = 1** | | | | | | | | | |
| Tijani, 2022 (1) | 18.22 | 2.2000 | 8 | 25.32 | 2.7000 | 8 | 79.9% | 54.7% | -2.73 [ -4.18; -1.27] |
| **dose = 2** | | | | | | | | | |
| Tijani, 2022 (2) | 11.12 | 0.4800 | 8 | 25.32 | 2.7000 | 8 | 20.1% | 45.3% | -6.92 [ -9.83; -4.01] |
| **Total (common effect, 95% CI)** | | | **16** | | | **16** | **100.0%** | . | **-3.57 [ -4.87; -2.26]** |
| **Total (random effect, 95% CI)** | | | | | | | . | **100.0%** | **-4.63 [ -8.72; -0.53]** |
| **Prediction interval** | | | | | | | | | **[-48.26; 39.01]** |

Heterogeneity: $Tau^2 = 7.4293$; $Chi^2 = 6.39$, df = 1 (P = 0.0115); $I^2 = 84.4\%$
Test for subgroup differences (common effect): $Chi^2 = 6.39$, df = 1 (P = 0.0115)
Test for subgroup differences (random effects): $Chi^2 = 6.39$, df = 1 (P = 0.0115)

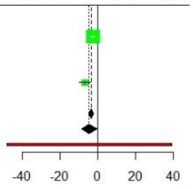


Dose 1: 200 mg/kg BW
Dose 2: 400 mg/kg BW
