# Supplementary figures and images for: Antinociceptive activity of Laportea species mediated by anti-inflammatory and antioxidant mechanisms: a systematic review and meta-analysis of in vivo animal studies
Source: BMC Complement Med Ther. 2026 Feb 3;26:85. doi: 10.1186/s12906-026-05262-0 (PMC12958739; doi:10.1186/s12906-026-05262-0)

ADDITIONAL FILE 6

Topical Inflammatory Effects

Paw Oedema Size: Subgroup Dose

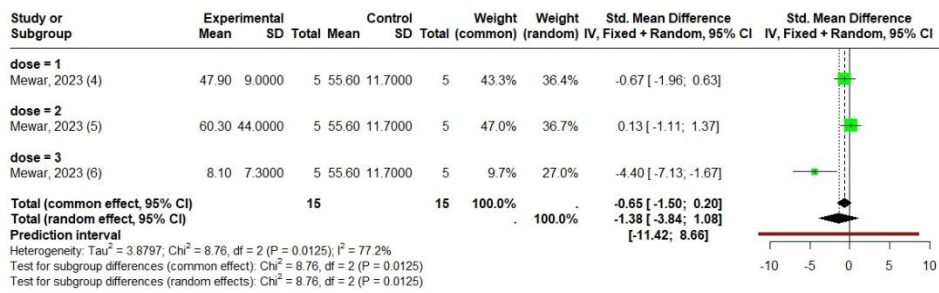

Dose 1: 0.5%

Dose 2: 1%

Dose 3: 2%

Supplement: Supplementary file 6 — Supplementary Material 6. [file 12906_2026_5262_MOESM6_ESM.pdf]
