# Supplementary material for: Antinociceptive activity of Laportea species mediated by anti-inflammatory and antioxidant mechanisms: a systematic review and meta-analysis of in vivo animal studies
Source: BMC Complement Med Ther. 2026 Feb 3;26:85. doi: 10.1186/s12906-026-05262-0 (PMC12958739; doi:10.1186/s12906-026-05262-0)
Supplement: Supplementary file 7 — Supplementary Material 7. [file 12906_2026_5262_MOESM7_ESM.pdf]

ADDITIONAL FILE 7
CELL DAMAGE: NITRITE OXIDASE

A. Funnel Plot

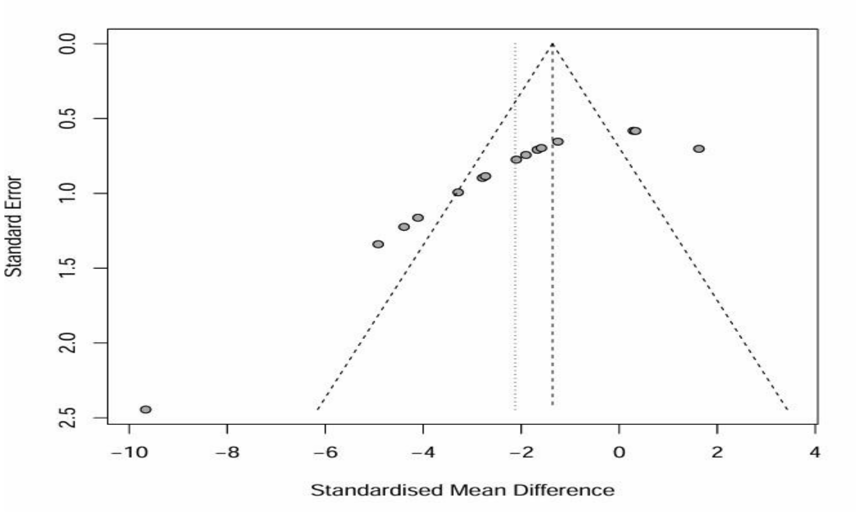

B. Egger's Test

Test result: t = -5.72, df = 13, p-value < 0.0001
Bias estimate: -6.5472 (SE = 1.1454)

C. Meta Regression

Mixed-effect model (k = 15)
R^2= 39,89 %; Q_M, p = 0.0078

Variable	β	SMD [95% CI]	p-value
tissue	0.33	10.14 [0.37; 16.57]	0.002
dose	0.55	0.13 [-0.94; 12.08]	0.811

D. Subgroup: Tissue

Study or Subgroup	Experimental Mean	Experimental SD	Experimental Total	Control Mean	Control SD	Control Total	Weight (common)	Weight (random)	Std. Mean Difference IV, Fixed + Random, 95% CI
**tissue = 3**									
Njina, 2016 (1)	17.85	1.3800	6	23.98	1.1900	6	2.8%	5.9%	-4.39 [-6.79; -1.99]
Njina, 2016 (2)	13.34	2.5600	6	23.98	1.1900	6	2.4%	5.5%	-4.92 [-7.54; -2.29]
Njina, 2016 (3)	13.72	0.7100	6	23.98	1.1900	6	0.7%	3.1%	-9.66 [-14.45; -4.87]
**Total (common effect, 95% CI)**			**18**			**18**	**5.9%**	.	**-5.24 [-6.90; -3.57]**
**Total (random effect, 95% CI)**							.	**14.5%**	**-5.50 [-7.71; -3.29]**
Heterogeneity: $Tau^2 = 1.4278$; $Chi^2 = 3.81$, df = 2 (P = 0.1486); $I^2 = 47.5\%$									
**tissue = 6**									
Njina, 2016 (10)	3.92	0.5800	6	11.22	2.8400	6	4.3%	6.5%	-3.29 [-5.23; -1.34]
Njina, 2016 (11)	6.38	0.9900	6	11.22	2.8400	6	7.1%	7.2%	-2.10 [-3.62; -0.58]
Njina, 2016 (12)	6.54	1.4900	6	11.22	2.8400	6	7.7%	7.2%	-1.90 [-3.36; -0.45]
**Total (common effect, 95% CI)**			**18**			**18**	**19.1%**	.	**-2.29 [-3.21; -1.36]**
**Total (random effect, 95% CI)**							.	**20.9%**	**-2.29 [-3.21; -1.36]**
Heterogeneity: $Tau^2 = 0$; $Chi^2 = 1.34$, df = 2 (P = 0.5126); $I^2 = 0\%$									
**tissue = 7**									
Njina, 2016 (13)	3.20	0.5000	6	3.01	0.7000	6	12.6%	7.7%	0.29 [-0.85; 1.43]
Njina, 2016 (14)	4.28	0.7400	6	3.01	0.7000	6	8.6%	7.4%	1.63 [0.25; 3.00]
Njina, 2016 (15)	2.04	0.2900	6	3.01	0.7000	6	8.5%	7.3%	-1.67 [-3.06; -0.28]
**Total (common effect, 95% CI)**			**18**			**18**	**29.7%**	.	**0.12 [-0.62; 0.86]**
**Total (random effect, 95% CI)**							.	**22.3%**	**0.09 [-1.75; 1.93]**
Heterogeneity: $Tau^2 = 2.1992$; $Chi^2 = 11.07$, df = 2 (P = 0.0039); $I^2 = 81.9\%$									
**tissue = 4**									
Njina, 2016 (4)	2.69	0.5300	6	5.30	1.1000	6	5.3%	6.8%	-2.79 [-4.55; -1.03]
Njina, 2016 (5)	2.94	0.2500	6	5.30	1.1000	6	5.4%	6.9%	-2.73 [-4.47; -0.99]
Njina, 2016 (6)	4.19	0.3600	6	5.30	1.1000	6	10.0%	7.5%	-1.25 [-2.53; 0.03]
**Total (common effect, 95% CI)**			**18**			**18**	**20.7%**	.	**-2.03 [-2.92; -1.14]**
**Total (random effect, 95% CI)**							.	**21.1%**	**-2.11 [-3.21; -1.02]**
Heterogeneity: $Tau^2 = 0.3033$; $Chi^2 = 2.76$, df = 2 (P = 0.2517); $I^2 = 27.5\%$									
**tissue = 5**									
Njina, 2016 (7)	10.50	2.3000	6	21.47	2.6200	6	3.2%	6.0%	-4.11 [-6.39; -1.83]
Njina, 2016 (8)	22.14	0.3000	6	21.47	2.6200	6	12.5%	7.6%	0.33 [-0.81; 1.47]
Njina, 2016 (9)	17.42	2.0500	6	21.47	2.6200	6	8.8%	7.4%	-1.59 [-2.95; -0.22]
**Total (common effect, 95% CI)**			**18**			**18**	**24.5%**	.	**-0.93 [-1.75; -0.11]**
**Total (random effect, 95% CI)**							.	**21.1%**	**-1.63 [-4.06; 0.80]**
Heterogeneity: $Tau^2 = 3.9223$; $Chi^2 = 13.03$, df = 2 (P = 0.0015); $I^2 = 84.7\%$									
**Total (common effect, 95% CI)**			**90**			**90**	**100.0%**	.	**-1.36 [-1.77; -0.96]**
**Total (random effect, 95% CI)**							.	**100.0%**	**-2.12 [-3.18; -1.06]**
**Prediction interval**									**[-6.29; 2.04]**
Heterogeneity: $Tau^2 = 3.4858$; $Chi^2 = 75.30$, df = 14 (P < 0.0001); $I^2 = 81.4\%$									
Test for subgroup differences (common effect): $Chi^2 = 43.29$, df = 4 (P < 0.0001)									
Test for subgroup differences (random effects): $Chi^2 = 14.80$, df = 4 (P = 0.0051)									

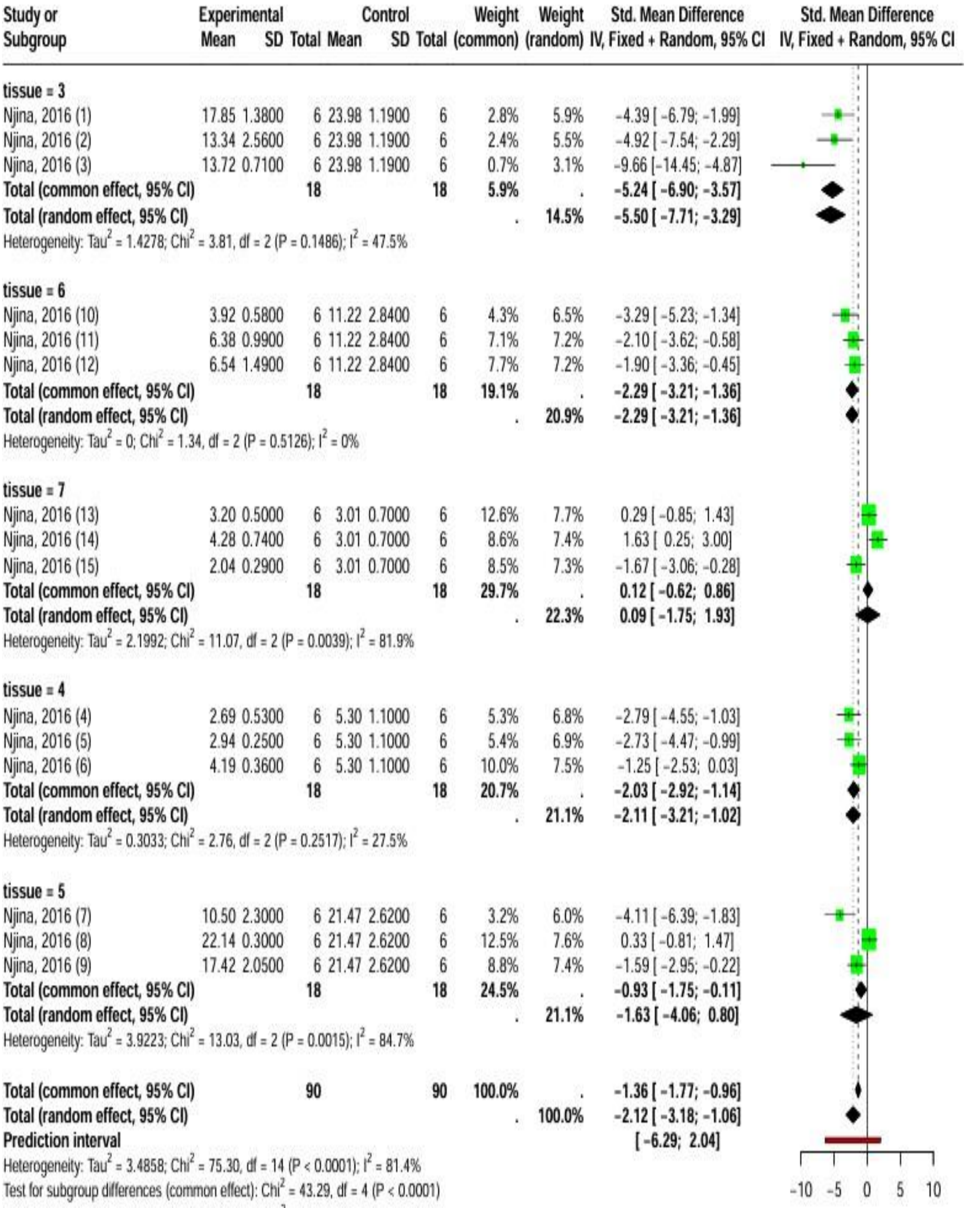

Tissue 3: liver
Tissue 4: heart
Tissue 5: lung
Tissue 6: kidney
Tissue 7: serum
