# Supplementary material for: Antinociceptive activity of Laportea species mediated by anti-inflammatory and antioxidant mechanisms: a systematic review and meta-analysis of in vivo animal studies
Source: BMC Complement Med Ther. 2026 Feb 3;26:85. doi: 10.1186/s12906-026-05262-0 (PMC12958739; doi:10.1186/s12906-026-05262-0)
Supplement: Supplementary file 8 — Supplementary Material 8. [file 12906_2026_5262_MOESM8_ESM.pdf]

ADDITIONAL FILE 8

CELL DAMAGE: Malondialdehyde

A. Meta Reggesion

Mixed-effect model (k = 7)
R^2= 92.54 %; Q_M, p = 0.026

Variable	β	SMD [95% CI]	p - value
Method	1.03	0.90 [-1.12; 2.93]	0.38
Tissue	1.03	0.90 [-1.12; 2.93]	0.38
Extract	0.94	2.35 [0,69; 3,97]	0.012
Duration	1.03	0.90 [-1.12; 2.93]	0.38
Dose	1.26	1.08 [-1.38; 3.54]	0.38

B. SUBGROUP: EXTRACT TYPE

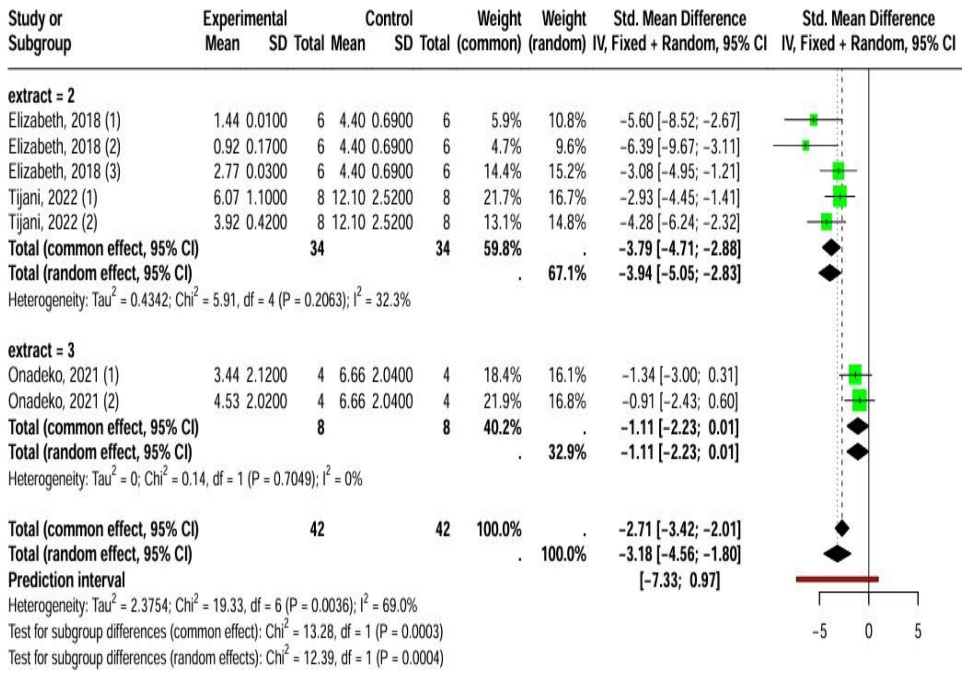

Extract 2: methanol

Extract 3: ethanol
