# Supplementary material for: Antinociceptive activity of Laportea species mediated by anti-inflammatory and antioxidant mechanisms: a systematic review and meta-analysis of in vivo animal studies
Source: BMC Complement Med Ther. 2026 Feb 3;26:85. doi: 10.1186/s12906-026-05262-0 (PMC12958739; doi:10.1186/s12906-026-05262-0)
Supplement: Supplementary file 9 — Supplementary Material 9. [file 12906_2026_5262_MOESM9_ESM.pdf]

Additional File 9

Antioxidant markers

Study or Subgroup	Experimental Mean	Experimental SD	Experimental Total	Control Mean	Control SD	Control Total	Weight (common)	Weight (random)	Std. Mean Difference IV, Fixed + Random, 95% CI
**outcome = 1**									
Elizabeth, 2018 (1)	3.92	0.5300	6	1.81	0.5800	6	0.8%	1.3%	3.50 [1.47; 5.54]
Elizabeth, 2018 (2)	4.66	1.4900	6	1.81	0.5800	6	1.3%	1.3%	2.33 [0.73; 3.92]
Elizabeth, 2018 (3)	3.35	0.2400	6	1.81	0.5800	6	0.9%	1.3%	3.20 [1.29; 5.11]
Omolola, 2018 (1)	22.80	0.0800	5	15.60	1.1400	5	0.2%	0.8%	8.04 [3.43; 12.66]
Omolola, 2018 (2)	27.50	0.4900	5	15.60	1.1400	5	0.1%	0.6%	12.24 [5.36; 19.13]
Omolola, 2018 (3)	30.60	2.0500	5	15.60	1.1400	5	0.2%	0.8%	8.16 [3.48; 12.85]
Tijani, 2022 (1)	21.46	2.0700	8	48.78	2.0700	8	0.1%	0.8%	-12.48 [-17.51; -7.44]
Tijani, 2022 (2)	47.31	2.0700	8	48.78	2.0700	8	3.3%	1.4%	-0.67 [-1.69; 0.34]
**Total (common effect, 95% CI)**			**49**			**49**	**6.8%**	.	**1.23 [0.53; 1.93]**
**Total (random effect, 95% CI)**							.	**8.4%**	**2.89 [-1.95; 7.73]**
Heterogeneity: $Tau^2 = 44.9577$; $Chi^2 = 79.29$, df = 7 ($P < 0.0001$); $I^2 = 91.2\%$									
**outcome = 2**									
Ganiyu, 2023 (1)	0.07	0.0070	5	0.05	0.0030	5	0.7%	1.2%	3.35 [1.12; 5.58]
Ganiyu, 2023 (2)	0.10	0.0070	5	0.05	0.0030	5	0.1%	0.8%	9.22 [3.97; 14.47]
Tijani, 2022 (1)	36.00	3.7600	8	32.44	3.7600	8	3.1%	1.4%	0.90 [-0.15; 1.94]
Tijani, 2022 (2)	57.33	3.7600	8	32.44	3.7600	8	0.5%	1.2%	6.26 [3.59; 8.92]
**Total (common effect, 95% CI)**			**26**			**26**	**4.4%**	.	**2.09 [1.21; 2.97]**
**Total (random effect, 95% CI)**							.	**4.6%**	**4.38 [1.08; 7.68]**
Heterogeneity: $Tau^2 = 9.1909$; $Chi^2 = 22.78$, df = 3 ($P < 0.0001$); $I^2 = 86.8\%$									
**outcome = 3**									
Elizabeth, 2018 (1)	75.73	0.0900	6	56.76	2.7100	6	0.2%	0.9%	9.13 [4.59; 13.67]
Elizabeth, 2018 (2)	83.83	0.0600	6	56.76	2.7100	6	0.1%	0.6%	13.03 [6.65; 19.41]
Elizabeth, 2018 (3)	74.87	1.1500	6	56.76	2.7100	6	0.2%	0.9%	8.03 [4.00; 12.06]
Ganiyu, 2023 (1)	1.57	0.0173	5	1.96	0.0310	5	0.1%	0.5%	-13.84 [-21.60; -6.09]
Ganiyu, 2023 (2)	1.54	0.0111	5	1.20	0.0310	5	0.1%	0.5%	13.22 [5.81; 20.64]
Njina, 2016 (1)	279.35	44.2500	6	176.29	35.4100	6	1.3%	1.3%	2.37 [0.76; 3.98]
Njina, 2016 (10)	92.68	15.7000	6	77.47	16.8700	6	2.3%	1.4%	0.86 [-0.34; 2.07]
Njina, 2016 (11)	92.26	12.9800	6	77.47	16.8700	6	2.3%	1.4%	0.91 [-0.31; 2.12]
Njina, 2016 (12)	89.64	16.6600	6	77.47	16.8700	6	2.4%	1.4%	0.67 [-0.51; 1.85]
Njina, 2016 (13)	41.37	3.9400	6	41.66	3.5300	6	2.6%	1.4%	-0.07 [-1.20; 1.06]
Njina, 2016 (14)	43.61	5.1500	6	41.66	3.5300	6	2.6%	1.4%	0.41 [-0.74; 1.56]
Njina, 2016 (15)	43.31	1.7600	6	41.66	3.5300	6	2.5%	1.4%	0.55 [-0.62; 1.71]
Njina, 2016 (2)	370.32	92.4800	6	176.29	35.4100	6	1.2%	1.3%	2.56 [0.88; 4.23]
Njina, 2016 (3)	132.21	26.7800	6	176.29	35.4100	6	2.0%	1.4%	-1.30 [-2.59; -0.00]
Njina, 2016 (4)	53.13	6.3700	6	32.04	6.9800	6	1.0%	1.3%	2.91 [1.11; 4.72]
Njina, 2016 (5)	40.06	5.2200	6	32.04	6.9800	6	2.1%	1.4%	1.20 [-0.07; 2.47]
Njina, 2016 (6)	47.83	2.6700	6	32.04	6.9800	6	1.1%	1.3%	2.76 [1.01; 4.50]
Njina, 2016 (7)	35.25	6.7700	6	41.35	7.6500	6	2.4%	1.4%	-0.78 [-1.97; 0.41]
Njina, 2016 (8)	45.67	6.5700	6	41.35	7.6500	6	2.5%	1.4%	0.56 [-0.60; 1.72]
Njina, 2016 (9)	56.12	13.7000	6	41.35	7.6500	6	2.1%	1.4%	1.23 [-0.05; 2.51]
Omolola, 2018 (1)	42.10	0.8900	5	35.50	0.9800	5	0.2%	1.0%	6.36 [2.63; 10.10]
Omolola, 2018 (2)	47.00	1.7200	5	35.50	0.9800	5	0.2%	0.9%	7.42 [3.13; 11.70]
Omolola, 2018 (3)	48.10	4.1700	5	35.50	0.9800	5	0.6%	1.2%	3.76 [1.34; 6.17]
Onadeko, 2021 (1)	59.70	25.0800	4	51.00	14.3400	4	1.7%	1.4%	0.37 [-1.04; 1.78]
Onadeko, 2021 (2)	77.61	63.2800	4	51.00	14.3400	4	1.7%	1.4%	0.50 [-0.92; 1.93]
Tijani, 2022 (1)	2.98	0.1400	8	2.00	0.1100	8	0.4%	1.1%	7.36 [4.29; 10.43]
Tijani, 2022 (2)	4.06	0.2000	8	2.00	0.1100	8	0.1%	0.8%	12.07 [7.19; 16.94]
**Total (common effect, 95% CI)**			**157**			**157**	**35.8%**	.	**1.04 [0.73; 1.35]**
**Total (random effect, 95% CI)**							.	**31.7%**	**2.72 [1.21; 4.22]**
Heterogeneity: $Tau^2 = 13.7175$; $Chi^2 = 162.33$, df = 26 ($P < 0.0001$); $I^2 = 84\%$									
**outcome = 4**									
Elizabeth, 2018 (1)	100.05	0.5800	6	66.15	1.0400	6	0.0%	0.1%	37.15 [19.21; 55.09]
Elizabeth, 2018 (2)	98.21	0.7400	6	66.15	1.0400	6	0.0%	0.2%	32.78 [16.94; 48.61]
Elizabeth, 2018 (3)	105.74	1.6000	6	66.15	1.0400	6	0.0%	0.2%	27.07 [13.98; 40.17]
Ganiyu, 2023 (1)	1.90	1.8980	5	1.76	0.0111	5	2.2%	1.4%	0.09 [-1.15; 1.33]
Ganiyu, 2023 (2)	1.82	0.0135	5	1.76	0.0111	5	0.5%	1.2%	4.02 [1.47; 6.56]
Njina, 2016 (1)	769.77	191.0000	6	442.80	94.7500	6	1.5%	1.4%	2.00 [0.51; 3.49]
Njina, 2016 (10)	153.54	20.1500	6	111.28	24.9400	6	1.7%	1.4%	1.72 [0.32; 3.12]
Njina, 2016 (11)	153.94	37.6600	6	111.28	24.9400	6	2.1%	1.4%	1.23 [-0.05; 2.51]
Njina, 2016 (12)	149.90	35.6000	6	111.28	24.9400	6	2.1%	1.4%	1.16 [-0.10; 2.42]
Njina, 2016 (13)	218.85	8.5500	6	133.11	13.8900	6	0.3%	1.0%	6.86 [3.37; 10.35]
Njina, 2016 (14)	246.12	46.7700	6	133.11	13.8900	6	1.0%	1.3%	3.02 [1.18; 4.87]
Njina, 2016 (15)	201.84	23.6400	6	133.11	13.8900	6	0.9%	1.3%	3.27 [1.33; 5.21]
Njina, 2016 (2)	644.53	139.2400	6	442.80	94.7500	6	1.8%	1.4%	1.56 [0.20; 2.92]
Njina, 2016 (3)	652.19	110.6300	6	442.80	94.7500	6	1.6%	1.4%	1.88 [0.43; 3.32]
Njina, 2016 (4)	9.13	1.6000	6	14.91	3.2900	6	1.5%	1.4%	-2.06 [-3.57; -0.56]
Njina, 2016 (5)	5.88	0.5000	6	14.91	3.2900	6	0.8%	1.3%	-3.54 [-5.59; -1.49]
Njina, 2016 (6)	18.85	5.4500	6	14.91	3.2900	6	2.3%	1.4%	0.81 [-0.39; 2.00]
Njina, 2016 (7)	69.91	16.2100	6	50.67	11.4900	6	2.0%	1.4%	1.26 [-0.02; 2.55]
Njina, 2016 (8)	130.98	21.6600	6	50.67	11.4900	6	0.6%	1.2%	4.27 [1.92; 6.62]
Njina, 2016 (9)	103.12	20.4700	6	50.67	11.4900	6	1.0%	1.3%	2.92 [1.11; 4.72]
Omolola, 2018 (1)	22.33	1.9400	5	11.36	2.3900	5	0.4%	1.2%	4.55 [1.74; 7.35]
Omolola, 2018 (2)	25.66	2.2200	5	11.36	2.3900	5	0.3%	1.1%	5.60 [2.26; 8.93]
Omolola, 2018 (3)	29.43	1.3800	5	11.36	2.3900	5	0.1%	0.8%	8.36 [3.57; 13.14]
Tijani, 2022 (1)	13.58	1.2400	8	8.03	2.0000	8	1.3%	1.3%	3.15 [1.57; 4.74]
Tijani, 2022 (2)	16.23	1.7500	8	8.03	2.0000	8	0.9%	1.3%	4.13 [2.22; 6.03]
**Total (common effect, 95% CI)**			**149**			**149**	**27.2%**	.	**1.67 [1.32; 2.02]**
**Total (random effect, 95% CI)**							.	**28.5%**	**2.73 [1.61; 3.86]**
Heterogeneity: $Tau^2 = 6.2752$; $Chi^2 = 152.38$, df = 24 ($P < 0.0001$); $I^2 = 84.2\%$									
**outcome = 5**									
Ganiyu, 2023 (1)	289.00	50.0000	5	151.00	13.0000	5	0.7%	1.2%	3.41 [1.15; 5.67]
Ganiyu, 2023 (2)	293.00	30.0000	5	151.00	13.0000	5	0.3%	1.1%	5.54 [2.24; 8.85]
**Total (common effect, 95% CI)**			**10**			**10**	**1.0%**	.	**4.09 [2.22; 5.95]**
**Total (random effect, 95% CI)**							.	**2.3%**	**4.12 [2.15; 6.09]**
Heterogeneity: $Tau^2 = 0.1912$; $Chi^2 = 1.09$, df = 1 ($P = 0.2961$); $I^2 = 8.4\%$									
**outcome = 6**									
Ganiyu, 2023 (1)	0.18	0.0150	5	0.07	0.0070	5	0.1%	0.8%	8.48 [3.63; 13.34]
Ganiyu, 2023 (2)	0.14	0.0150	5	0.07	0.0070	5	0.3%	1.1%	5.40 [2.17; 8.63]
Tijani, 2022 (1)	13.50	1.5800	8	8.09	1.8000	8	1.4%	1.3%	3.02 [1.47; 4.57]
Tijani, 2022 (2)	16.40	1.5700	8	8.09	1.8000	8	0.8%	1.3%	4.65 [2.57; 6.74]
**Total (common effect, 95% CI)**			**26**			**26**	**2.6%**	.	**4.08 [2.95; 5.21]**
**Total (random effect, 95% CI)**							.	**4.5%**	**4.56 [2.81; 6.31]**
Heterogeneity: $Tau^2 = 1.4240$; $Chi^2 = 5.9$, df = 3 ($P = 0.1166$); $I^2 = 49.1\%$									
**outcome = 7**									
Njina, 2016 (1)	318.96	31.1900	6	279.75	41.2700	6	2.2%	1.4%	0.99 [-0.24; 2.22]
Njina, 2016 (10)	34.91	7.4400	6	20.46	2.0700	6	1.3%	1.3%	2.44 [0.81; 4.07]
Njina, 2016 (11)	31.89	5.7500	6	20.46	2.0700	6	1.3%	1.3%	2.44 [0.81; 4.07]
Njina, 2016 (12)	30.42	2.4800	6	20.46	2.0700	6	0.7%	1.2%	4.02 [1.78; 6.27]
Njina, 2016 (13)	0.61	0.1000	6	0.49	0.0800	6	2.1%	1.4%	1.22 [-0.05; 2.50]
Njina, 2016 (14)	0.65	0.1300	6	0.49	0.0800	6	2.0%	1.4%	1.37 [0.06; 2.68]
Njina, 2016 (15)	0.43	0.0300	6	0.49	0.0800	6	2.3%	1.4%	-0.92 [-2.13; 0.30]
Njina, 2016 (2)	392.68	77.4300	6	279.75	41.2700	6	1.7%	1.4%	1.68 [0.29; 3.07]
Njina, 2016 (3)	406.93	42.6900	6	279.75	41.2700	6	1.1%	1.3%	2.80 [1.04; 4.55]
Njina, 2016 (4)	22.77	6.4600	6	15.37	3.3100	6	2.0%	1.4%	1.33 [0.03; 2.63]
Njina, 2016 (5)	27.49	5.3200	6	15.37	3.3100	6	1.2%	1.3%	2.52 [0.86; 4.19]
Njina, 2016 (6)	25.54	4.7000	6	15.37	3.3100	6	1.3%	1.3%	2.31 [0.72; 3.90]
Njina, 2016 (7)	78.87	13.7500	6	37.43	3.4300	6	0.7%	1.3%	3.82 [1.66; 5.97]
Njina, 2016 (8)	62.54	8.4500	6	37.43	3.4300	6	0.8%	1.3%	3.59 [1.52; 5.66]
Njina, 2016 (9)	50.83	8.5000	6	37.43	3.4300	6	1.6%	1.4%	1.91 [0.45; 3.37]
**Total (common effect, 95% CI)**			**90**			**90**	**22.2%**	.	**1.69 [1.30; 2.08]**
**Total (random effect, 95% CI)**							.	**20.1%**	**1.92 [1.29; 2.55]**
Heterogeneity: $Tau^2 = 0.9140$; $Chi^2 = 35.89$, df = 14 ($P = 0.0011$); $I^2 = 61\%$									
**Total (common effect, 95% CI)**			**507**			**507**	**100.0%**	.	**1.52 [1.34; 1.71]**
**Total (random effect, 95% CI)**							.	**100.0%**	**2.77 [2.11; 3.42]**
**Prediction interval**									**[-2.82; 8.35]**
Heterogeneity: $Tau^2 = 7.7782$; $Chi^2 = 499.88$, df = 84 ($P < 0.0001$); $I^2 = 83.2\%$									
Test for subgroup differences (common effect): $Chi^2 = 40.22$, df = 6 ($P < 0.0001$)									
Test for subgroup differences (random effects): $Chi^2 = 12.68$, df = 6 ($P = 0.0484$)									

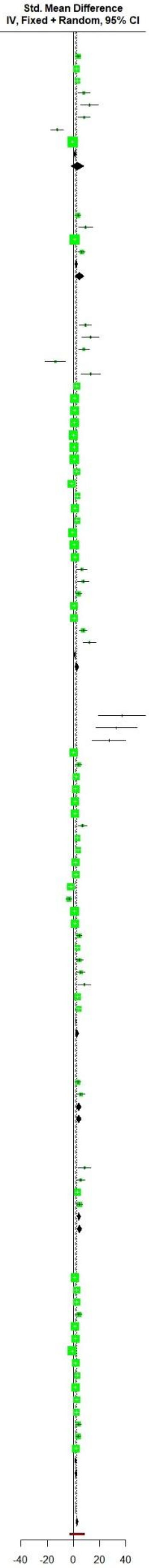

Outcome 1: Glutathione
Outcome 2: Glutatahione peroxidase
Outcome 3: Superoxidase dismutase
Outcome 4: Catalase
Outcome 4: Glutathione reductase
