# Supplementary material for: Antinociceptive activity of Laportea species mediated by anti-inflammatory and antioxidant mechanisms: a systematic review and meta-analysis of in vivo animal studies
Source: BMC Complement Med Ther. 2026 Feb 3;26:85. doi: 10.1186/s12906-026-05262-0 (PMC12958739; doi:10.1186/s12906-026-05262-0)
Supplement: Supplementary file 10 — Supplementary Material 10. [file 12906_2026_5262_MOESM10_ESM.pdf]

ADDITIONAL FILE 10

ANTIOXIDANT: SUPEROXIDE DISMUTASE

A. Meta-regression

Mixed-effect model (k = 27)
R^2= 91,81 %, QM, p < 0.0001

Variabel	β	SMD [95% CI]	p value
laportea_sp	2.68	18.54 [13.27; 23.80]	<0,0001
tissue	0.23	-0.24 [-0.69; 0.22]	0.20
duration	17.31	90.15 [56.22; 124.08]	<0.0001
dose	0.39	-0.1 5 [-0.94; 0.63]	0.64
extract	2.67	-16.34 [-21.57; -11.11]	<0.0001
method	1.34	9.27 [6.64; 11.90]	<0,0001
animal_sp	1.98	-0.76 [-4.66; 3.13]	0.69

B. Subgroup: Laportea species

Study or Subgroup	Experimental Mean	Experimental SD	Experimental Total	Control Mean	Control SD	Control Total	Weight (common)	Weight (random)	Std. Mean Difference IV, Fixed + Random, 95% CI
**laportea_sp = 2**									
Elizabeth, 2018 (1)	75.73	0.0900	6	56.76	2.7100	6	0.5%	3.1%	9.13 [4.59; 13.67]
Elizabeth, 2018 (2)	83.83	0.0600	6	56.76	2.7100	6	0.2%	2.4%	13.03 [6.65; 19.41]
Elizabeth, 2018 (3)	74.87	1.1500	6	56.76	2.7100	6	0.6%	3.3%	8.03 [4.00; 12.06]
Ganiyu, 2023 (1)	1.57	0.0173	5	1.96	0.0310	5	0.2%	2.0%	-13.84 [-21.60; -6.09]
Ganiyu, 2023 (2)	1.54	0.0111	5	1.20	0.0310	5	0.2%	2.1%	13.22 [5.81; 20.64]
Omolola, 2018 (1)	42.10	0.8900	5	35.50	0.9800	5	0.7%	3.4%	6.36 [2.63; 10.10]
Omolola, 2018 (2)	47.00	1.7200	5	35.50	0.9800	5	0.5%	3.2%	7.42 [3.13; 11.70]
Omolola, 2018 (3)	48.10	4.1700	5	35.50	0.9800	5	1.6%	3.8%	3.76 [1.34; 6.17]
Onadeko, 2021 (1)	59.70	25.0800	4	51.00	14.3400	4	4.7%	4.1%	0.37 [-1.04; 1.78]
Onadeko, 2021 (2)	77.61	63.2800	4	51.00	14.3400	4	4.6%	4.1%	0.50 [-0.92; 1.93]
Tijani, 2022 (1)	2.98	0.1400	8	2.00	0.1100	8	1.0%	3.6%	7.36 [4.29; 10.43]
Tijani, 2022 (2)	4.06	0.2000	8	2.00	0.1100	8	0.4%	2.9%	12.07 [7.19; 16.94]
**Total (common effect, 95% CI)**			**67**			**67**	**15.1%**	.	**2.79 [2.00; 3.58]**
**Total (random effect, 95% CI)**							.	**38.0%**	**5.64 [1.96; 9.32]**
Heterogeneity: $Tau^2 = 37.0524$; $Chi^2 = 101.38$, df = 11 ($P < 0.0001$); $I^2 = 89.2\%$									
**laportea_sp = 1**									
Njina, 2016 (1)	279.35	44.2500	6	176.29	35.4100	6	3.6%	4.1%	2.37 [0.76; 3.98]
Njina, 2016 (10)	92.68	15.7000	6	77.47	16.8700	6	6.5%	4.2%	0.86 [-0.34; 2.07]
Njina, 2016 (11)	92.26	12.9800	6	77.47	16.8700	6	6.4%	4.2%	0.91 [-0.31; 2.12]
Njina, 2016 (12)	89.64	16.6600	6	77.47	16.8700	6	6.8%	4.2%	0.67 [-0.51; 1.85]
Njina, 2016 (13)	41.37	3.9400	6	41.66	3.5300	6	7.3%	4.2%	-0.07 [-1.20; 1.06]
Njina, 2016 (14)	43.61	5.1500	6	41.66	3.5300	6	7.1%	4.2%	0.41 [-0.74; 1.56]
Njina, 2016 (15)	43.31	1.7600	6	41.66	3.5300	6	7.0%	4.2%	0.55 [-0.62; 1.71]
Njina, 2016 (2)	370.32	92.4800	6	176.29	35.4100	6	3.4%	4.1%	2.56 [0.88; 4.23]
Njina, 2016 (3)	132.21	26.7800	6	176.29	35.4100	6	5.6%	4.1%	-1.30 [-2.59; -0.00]
Njina, 2016 (4)	53.13	6.3700	6	32.04	6.9800	6	2.9%	4.0%	2.91 [1.11; 4.72]
Njina, 2016 (5)	40.06	5.2200	6	32.04	6.9800	6	5.8%	4.1%	1.20 [-0.07; 2.47]
Njina, 2016 (6)	47.83	2.6700	6	32.04	6.9800	6	3.1%	4.0%	2.76 [1.01; 4.50]
Njina, 2016 (7)	35.25	6.7700	6	41.35	7.6500	6	6.6%	4.2%	-0.78 [-1.97; 0.41]
Njina, 2016 (8)	45.67	6.5700	6	41.35	7.6500	6	7.0%	4.2%	0.56 [-0.60; 1.72]
Njina, 2016 (9)	56.12	13.7000	6	41.35	7.6500	6	5.8%	4.1%	1.23 [-0.05; 2.51]
**Total (common effect, 95% CI)**			**90**			**90**	**84.9%**	.	**0.73 [0.40; 1.06]**
**Total (random effect, 95% CI)**							.	**62.0%**	**0.87 [0.30; 1.45]**
Heterogeneity: $Tau^2 = 0.8468$; $Chi^2 = 38.65$, df = 14 ($P = 0.0004$); $I^2 = 63.8\%$									
**Total (common effect, 95% CI)**			**157**			**157**	**100.0%**	.	**1.04 [0.73; 1.35]**
**Total (random effect, 95% CI)**							.	**100.0%**	**2.72 [1.21; 4.22]**
**Prediction interval**									**[-5.06; 10.49]**

Heterogeneity: $Tau^2 = 13.7175$; $Chi^2 = 162.33$, df = 26 ($P < 0.0001$); $I^2 = 84.0\%$
Test for subgroup differences (common effect): $Chi^2 = 22.29$, df = 1 ($P < 0.0001$)
Test for subgroup differences (random effects): $Chi^2 = 6.28$, df = 1 ($P = 0.0122$)

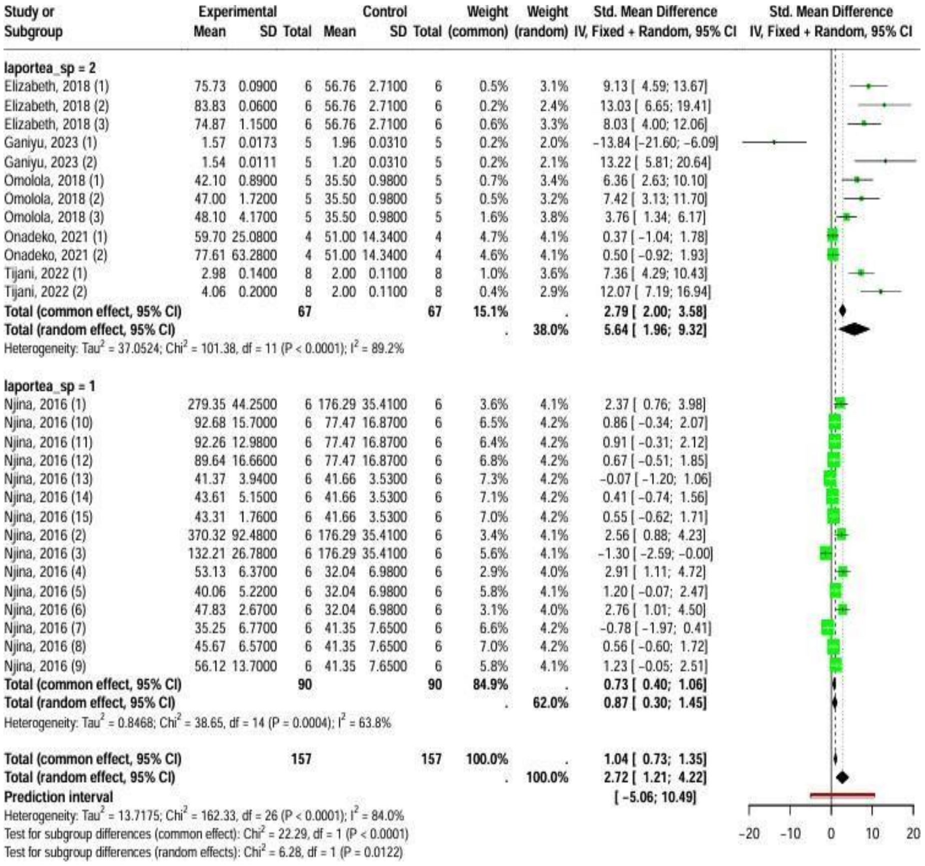

Laportea 1: L. ovalifolia
Laportea 2: L. aestuans

C. Subgroup: Method

Study or Subgroup	Experimental Mean	Experimental SD	Experimental Total	Control Mean	Control SD	Control Total	Weight (common)	Weight (random)	Std. Mean Difference IV, Fixed + Random, 95% CI
**method = 2**									
Elizabeth, 2018 (1)	75.73	0.0900	6	56.76	2.7100	6	0.5%	3.1%	9.13 [4.59; 13.67]
Elizabeth, 2018 (2)	83.83	0.0600	6	56.76	2.7100	6	0.2%	2.4%	13.03 [6.65; 19.41]
Elizabeth, 2018 (3)	74.87	1.1500	6	56.76	2.7100	6	0.6%	3.3%	8.03 [4.00; 12.06]
**Total (common effect, 95% CI)**			**18**			**18**	**1.3%**	.	**9.34 [6.61; 12.06]**
**Total (random effect, 95% CI)**							.	**8.8%**	**9.34 [6.61; 12.06]**
Heterogeneity: Tau^2 = < 0.0001; Chi^2 = 1.7, df = 2 (P = 0.4270); I^2 = 0%									
**method = 4**									
Ganiyu, 2023 (1)	1.57	0.0173	5	1.96	0.0310	5	0.2%	2.0%	-13.84 [-21.60; -6.09]
Ganiyu, 2023 (2)	1.54	0.0111	5	1.20	0.0310	5	0.2%	2.1%	13.22 [5.81; 20.64]
**Total (common effect, 95% CI)**			**10**			**10**	**0.3%**	.	**0.30 [-5.06; 5.65]**
**Total (random effect, 95% CI)**							.	**4.1%**	**-0.29 [-26.81; 26.24]**
Heterogeneity: Tau^2 = 351.2848; Chi^2 = 24.44, df = 1 (P < 0.0001); I^2 = 95.9%									
**method = 1**									
Njina, 2016 (1)	279.35	44.2500	6	176.29	35.4100	6	3.6%	4.1%	2.37 [0.76; 3.98]
Njina, 2016 (10)	92.68	15.7000	6	77.47	16.8700	6	6.5%	4.2%	0.86 [-0.34; 2.07]
Njina, 2016 (11)	92.26	12.9800	6	77.47	16.8700	6	6.4%	4.2%	0.91 [-0.31; 2.12]
Njina, 2016 (12)	89.64	16.6600	6	77.47	16.8700	6	6.8%	4.2%	0.67 [-0.51; 1.85]
Njina, 2016 (13)	41.37	3.9400	6	41.66	3.5300	6	7.3%	4.2%	-0.07 [-1.20; 1.06]
Njina, 2016 (14)	43.61	5.1500	6	41.66	3.5300	6	7.1%	4.2%	0.41 [-0.74; 1.56]
Njina, 2016 (15)	43.31	1.7600	6	41.66	3.5300	6	7.0%	4.2%	0.55 [-0.62; 1.71]
Njina, 2016 (2)	370.32	92.4800	6	176.29	35.4100	6	3.4%	4.1%	2.56 [0.88; 4.23]
Njina, 2016 (3)	132.21	26.7800	6	176.29	35.4100	6	5.6%	4.1%	-1.30 [-2.59; -0.00]
Njina, 2016 (4)	53.13	6.3700	6	32.04	6.9800	6	2.9%	4.0%	2.91 [1.11; 4.72]
Njina, 2016 (5)	40.06	5.2200	6	32.04	6.9800	6	5.8%	4.1%	1.20 [-0.07; 2.47]
Njina, 2016 (6)	47.83	2.6700	6	32.04	6.9800	6	3.1%	4.0%	2.76 [1.01; 4.50]
Njina, 2016 (7)	35.25	6.7700	6	41.35	7.6500	6	6.6%	4.2%	-0.78 [-1.97; 0.41]
Njina, 2016 (8)	45.67	6.5700	6	41.35	7.6500	6	7.0%	4.2%	0.56 [-0.60; 1.72]
Njina, 2016 (9)	56.12	13.7000	6	41.35	7.6500	6	5.8%	4.1%	1.23 [-0.05; 2.51]
**Total (common effect, 95% CI)**			**90**			**90**	**84.9%**	.	**0.73 [0.40; 1.06]**
**Total (random effect, 95% CI)**							.	**62.0%**	**0.87 [0.30; 1.45]**
Heterogeneity: Tau^2 = 0.8468; Chi^2 = 38.65, df = 14 (P = 0.0004); I^2 = 63.8%									
**method = 5**									
Omolola, 2018 (1)	42.10	0.8900	5	35.50	0.9800	5	0.7%	3.4%	6.36 [2.63; 10.10]
Omolola, 2018 (2)	47.00	1.7200	5	35.50	0.9800	5	0.5%	3.2%	7.42 [3.13; 11.70]
Omolola, 2018 (3)	48.10	4.1700	5	35.50	0.9800	5	1.6%	3.8%	3.76 [1.34; 6.17]
**Total (common effect, 95% CI)**			**15**			**15**	**2.8%**	.	**5.06 [3.22; 6.89]**
**Total (random effect, 95% CI)**							.	**10.4%**	**5.34 [2.99; 7.69]**
Heterogeneity: Tau^2 = 1.4404; Chi^2 = 2.75, df = 2 (P = 0.2529); I^2 = 27.3%									
**method = 3**									
Onadeko, 2021 (1)	59.70	25.0800	4	51.00	14.3400	4	4.7%	4.1%	0.37 [-1.04; 1.78]
Onadeko, 2021 (2)	77.61	63.2800	4	51.00	14.3400	4	4.6%	4.1%	0.50 [-0.92; 1.93]
Tijani, 2022 (1)	2.98	0.1400	8	2.00	0.1100	8	1.0%	3.6%	7.36 [4.29; 10.43]
Tijani, 2022 (2)	4.06	0.2000	8	2.00	0.1100	8	0.4%	2.9%	12.07 [7.19; 16.94]
**Total (common effect, 95% CI)**			**24**			**24**	**10.8%**	.	**1.50 [0.57; 2.44]**
**Total (random effect, 95% CI)**							.	**14.8%**	**4.73 [-0.66; 10.11]**
Heterogeneity: Tau^2 = 27.9423; Chi^2 = 36.37, df = 3 (P < 0.0001); I^2 = 91.8%									
**Total (common effect, 95% CI)**			**157**			**157**	**100.0%**	.	**1.04 [0.73; 1.35]**
**Total (random effect, 95% CI)**							.	**100.0%**	**2.72 [1.21; 4.22]**
**Prediction interval**									**[-5.06; 10.49]**
Heterogeneity: Tau^2 = 13.7175; Chi^2 = 162.33, df = 26 (P < 0.0001); I^2 = 84.0%									
Test for subgroup differences (common effect): Chi^2 = 58.42, df = 4 (P < 0.0001)									
Test for subgroup differences (random effects): Chi^2 = 47.92, df = 4 (P < 0.0001)									

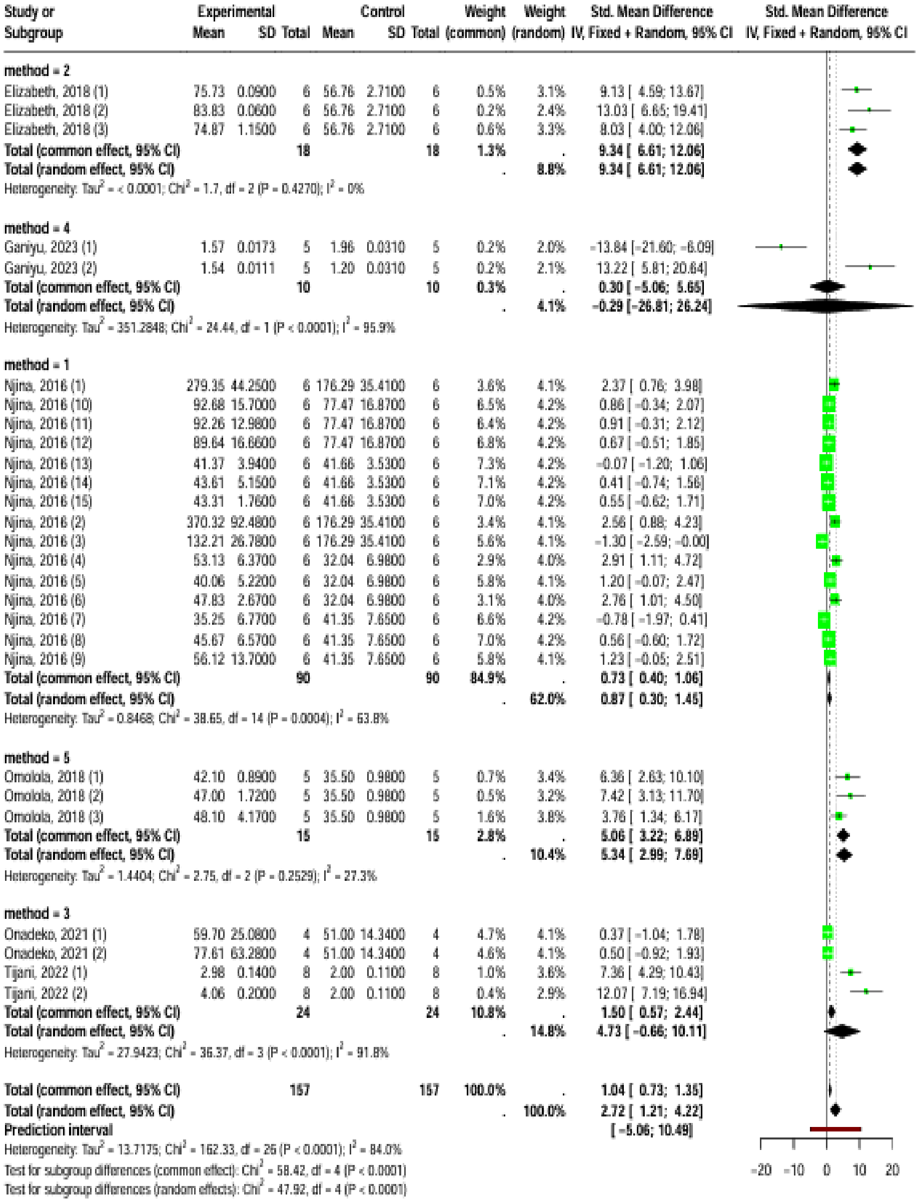

Method 1: Castrated rat

Method 2: Diclofenac induced rat

Method 3: Ulcer

Method 4: Liver injury

Method 5: BPH

D. Subgroup: Duration

Study or Subgroup	Experimental Mean	Experimental SD	Experimental Total	Control Mean	Control SD	Control Total	Weight (common)	Weight (random)	Std. Mean Difference IV, Fixed + Random, 95% CI
**duration = 1**									
Elizabeth, 2018 (1)	75.73	0.0900	6	56.76	2.7100	6	0.5%	3.1%	9.13 [4.59; 13.67]
Elizabeth, 2018 (2)	83.83	0.0600	6	56.76	2.7100	6	0.2%	2.4%	13.03 [6.65; 19.41]
Elizabeth, 2018 (3)	74.87	1.1500	6	56.76	2.7100	6	0.6%	3.3%	8.03 [4.00; 12.06]
Ganiyu, 2023 (1)	1.57	0.0173	5	1.96	0.0310	5	0.2%	2.0%	-13.84 [-21.60; -6.09]
Ganiyu, 2023 (2)	1.54	0.0111	5	1.20	0.0310	5	0.2%	2.1%	13.22 [5.81; 20.64]
Onadeko, 2021 (1)	59.70	25.0800	4	51.00	14.3400	4	4.7%	4.1%	0.37 [-1.04; 1.78]
Onadeko, 2021 (2)	77.61	63.2800	4	51.00	14.3400	4	4.6%	4.1%	0.50 [-0.92; 1.93]
**Total (common effect, 95% CI)**			**36**			**36**	**11.0%**	.	**1.46 [0.53; 2.39]**
**Total (random effect, 95% CI)**							.	**21.1%**	**4.38 [-2.24; 10.99]**
Heterogeneity: $Tau^2 = 72.8150$; $Chi^2 = 62.44$, df = 6 ($P < 0.0001$); $I^2 = 90.4\%$									
**duration = 3**									
Njina, 2016 (1)	279.35	44.2500	6	176.29	35.4100	6	3.6%	4.1%	2.37 [0.76; 3.98]
Njina, 2016 (10)	92.68	15.7000	6	77.47	16.8700	6	6.5%	4.2%	0.86 [-0.34; 2.07]
Njina, 2016 (11)	92.26	12.9800	6	77.47	16.8700	6	6.4%	4.2%	0.91 [-0.31; 2.12]
Njina, 2016 (12)	89.64	16.6600	6	77.47	16.8700	6	6.8%	4.2%	0.67 [-0.51; 1.85]
Njina, 2016 (13)	41.37	3.9400	6	41.66	3.5300	6	7.3%	4.2%	-0.07 [-1.20; 1.06]
Njina, 2016 (14)	43.61	5.1500	6	41.66	3.5300	6	7.1%	4.2%	0.41 [-0.74; 1.56]
Njina, 2016 (15)	43.31	1.7600	6	41.66	3.5300	6	7.0%	4.2%	0.55 [-0.62; 1.71]
Njina, 2016 (2)	370.32	92.4800	6	176.29	35.4100	6	3.4%	4.1%	2.56 [0.88; 4.23]
Njina, 2016 (3)	132.21	26.7800	6	176.29	35.4100	6	5.6%	4.1%	-1.30 [-2.59; -0.00]
Njina, 2016 (4)	53.13	6.3700	6	32.04	6.9800	6	2.9%	4.0%	2.91 [1.11; 4.72]
Njina, 2016 (5)	40.06	5.2200	6	32.04	6.9800	6	5.8%	4.1%	1.20 [-0.07; 2.47]
Njina, 2016 (6)	47.83	2.6700	6	32.04	6.9800	6	3.1%	4.0%	2.76 [1.01; 4.50]
Njina, 2016 (7)	35.25	6.7700	6	41.35	7.6500	6	6.6%	4.2%	-0.78 [-1.97; 0.41]
Njina, 2016 (8)	45.67	6.5700	6	41.35	7.6500	6	7.0%	4.2%	0.56 [-0.60; 1.72]
Njina, 2016 (9)	56.12	13.7000	6	41.35	7.6500	6	5.8%	4.1%	1.23 [-0.05; 2.51]
Omolola, 2018 (1)	42.10	0.8900	5	35.50	0.9800	5	0.7%	3.4%	6.36 [2.63; 10.10]
Omolola, 2018 (2)	47.00	1.7200	5	35.50	0.9800	5	0.5%	3.2%	7.42 [3.13; 11.70]
Omolola, 2018 (3)	48.10	4.1700	5	35.50	0.9800	5	1.6%	3.8%	3.76 [1.34; 6.17]
**Total (common effect, 95% CI)**			**105**			**105**	**87.6%**	.	**0.87 [0.54; 1.19]**
**Total (random effect, 95% CI)**							.	**72.4%**	**1.30 [0.59; 2.02]**
Heterogeneity: $Tau^2 = 1.7177$; $Chi^2 = 62.11$, df = 17 ($P < 0.0001$); $I^2 = 72.6\%$									
**duration = 2**									
Tijani, 2022 (1)	2.98	0.1400	8	2.00	0.1100	8	1.0%	3.6%	7.36 [4.29; 10.43]
Tijani, 2022 (2)	4.06	0.2000	8	2.00	0.1100	8	0.4%	2.9%	12.07 [7.19; 16.94]
**Total (common effect, 95% CI)**			**16**			**16**	**1.4%**	.	**8.70 [6.10; 11.30]**
**Total (random effect, 95% CI)**							.	**6.6%**	**9.32 [4.77; 13.86]**
Heterogeneity: $Tau^2 = 6.7562$; $Chi^2 = 2.56$, df = 1 ($P = 0.1093$); $I^2 = 61\%$									
**Total (common effect, 95% CI)**			**157**			**157**	**100.0%**	.	**1.04 [0.73; 1.35]**
**Total (random effect, 95% CI)**							.	**100.0%**	**2.72 [1.21; 4.22]**
**Prediction interval**									**[-5.06; 10.49]**
Heterogeneity: $Tau^2 = 13.7175$; $Chi^2 = 162.33$, df = 26 ($P < 0.0001$); $I^2 = 84.0\%$									
Test for subgroup differences (common effect): $Chi^2 = 35.21$, df = 2 ($P < 0.0001$)									
Test for subgroup differences (random effects): $Chi^2 = 12.37$, df = 2 ($P = 0.0021$)									

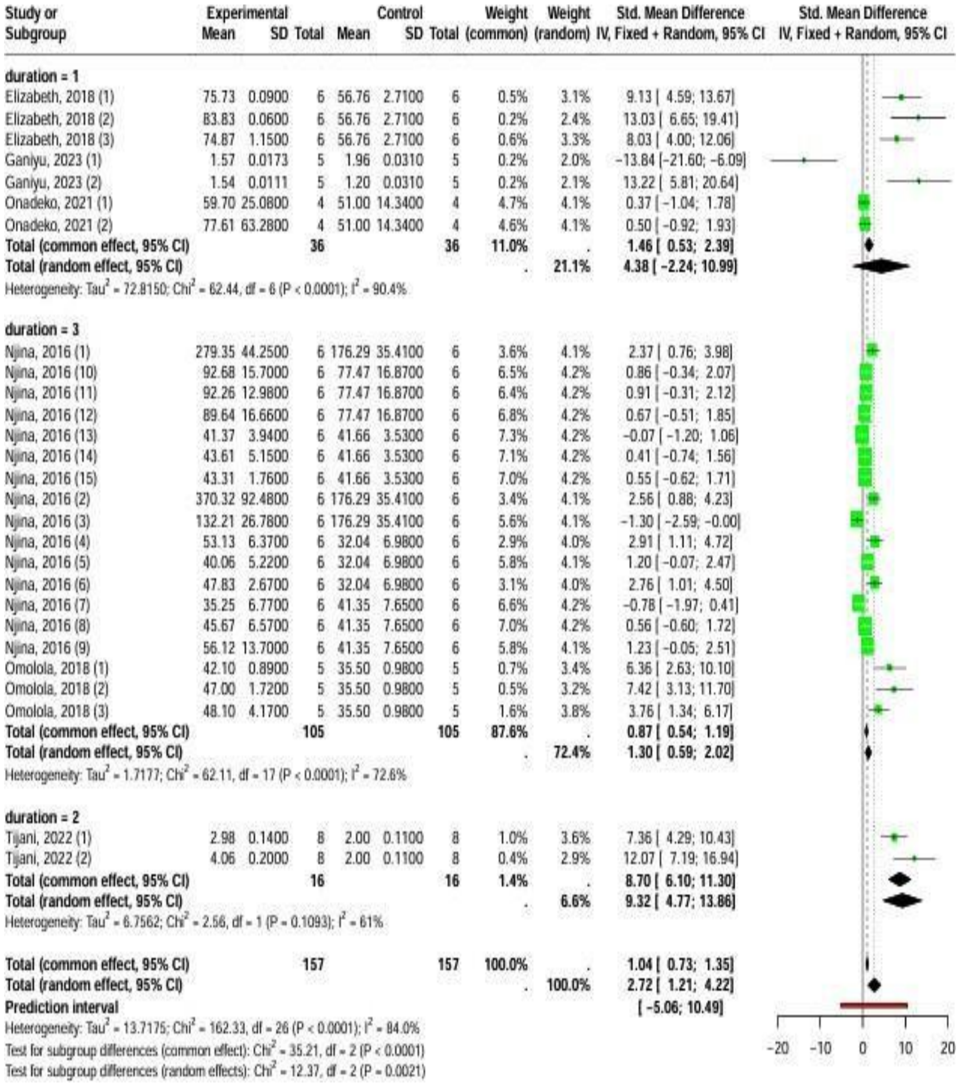

Duration 1: 1- 3 day
Duration 2: 4 - 7 day
Duration 3: > 7 day

E. Subgroup Extract

Study or Subgroup	Experimental Mean	Experimental SD	Experimental Total	Control Mean	Control SD	Control Total	Weight (common)	Weight (random)	Std. Mean Difference IV, Fixed + Random, 95% CI
**extract = 2**									
Elizabeth, 2018 (1)	75.73	0.0900	6	56.76	2.7100	6	0.5%	3.1%	9.13 [4.59; 13.67]
Elizabeth, 2018 (2)	83.83	0.0600	6	56.76	2.7100	6	0.2%	2.4%	13.03 [6.65; 19.41]
Elizabeth, 2018 (3)	74.87	1.1500	6	56.76	2.7100	6	0.6%	3.3%	8.03 [4.00; 12.06]
Tijani, 2022 (1)	2.98	0.1400	8	2.00	0.1100	8	1.0%	3.6%	7.36 [4.29; 10.43]
Tijani, 2022 (2)	4.06	0.2000	8	2.00	0.1100	8	0.4%	2.9%	12.07 [7.19; 16.94]
**Total (common effect, 95% CI)**			**34**			**34**	**2.7%**	.	**9.00 [7.12; 10.88]**
**Total (random effect, 95% CI)**							.	**15.3%**	**9.07 [7.09; 11.05]**
Heterogeneity: $Tau^2 = 0.3962$; $Chi^2 = 4.38$, df = 4 (P = 0.3574); $I^2 = 8.6\%$									
**extract = 1**									
Ganiyu, 2023 (1)	1.57	0.0173	5	1.96	0.0310	5	0.2%	2.0%	-13.84 [-21.60; -6.09]
Ganiyu, 2023 (2)	1.54	0.0111	5	1.20	0.0310	5	0.2%	2.1%	13.22 [5.81; 20.64]
Njina, 2016 (1)	279.35	44.2500	6	176.29	35.4100	6	3.6%	4.1%	2.37 [0.76; 3.98]
Njina, 2016 (10)	92.68	15.7000	6	77.47	16.8700	6	6.5%	4.2%	0.86 [-0.34; 2.07]
Njina, 2016 (11)	92.26	12.9800	6	77.47	16.8700	6	6.4%	4.2%	0.91 [-0.31; 2.12]
Njina, 2016 (12)	89.64	16.6600	6	77.47	16.8700	6	6.8%	4.2%	0.67 [-0.51; 1.85]
Njina, 2016 (13)	41.37	3.9400	6	41.66	3.5300	6	7.3%	4.2%	-0.07 [-1.20; 1.06]
Njina, 2016 (14)	43.61	5.1500	6	41.66	3.5300	6	7.1%	4.2%	0.41 [-0.74; 1.56]
Njina, 2016 (15)	43.31	1.7600	6	41.66	3.5300	6	7.0%	4.2%	0.55 [-0.62; 1.71]
Njina, 2016 (2)	370.32	92.4800	6	176.29	35.4100	6	3.4%	4.1%	2.56 [0.88; 4.23]
Njina, 2016 (3)	132.21	26.7800	6	176.29	35.4100	6	5.6%	4.1%	-1.30 [-2.59; -0.00]
Njina, 2016 (4)	53.13	6.3700	6	32.04	6.9800	6	2.9%	4.0%	2.91 [1.11; 4.72]
Njina, 2016 (5)	40.06	5.2200	6	32.04	6.9800	6	5.8%	4.1%	1.20 [-0.07; 2.47]
Njina, 2016 (6)	47.83	2.6700	6	32.04	6.9800	6	3.1%	4.0%	2.76 [1.01; 4.50]
Njina, 2016 (7)	35.25	6.7700	6	41.35	7.6500	6	6.6%	4.2%	-0.78 [-1.97; 0.41]
Njina, 2016 (8)	45.67	6.5700	6	41.35	7.6500	6	7.0%	4.2%	0.56 [-0.60; 1.72]
Njina, 2016 (9)	56.12	13.7000	6	41.35	7.6500	6	5.8%	4.1%	1.23 [-0.05; 2.51]
**Total (common effect, 95% CI)**			**100**			**100**	**85.2%**	.	**0.73 [0.39; 1.06]**
**Total (random effect, 95% CI)**							.	**66.0%**	**0.88 [0.25; 1.51]**
Heterogeneity: $Tau^2 = 1.1009$; $Chi^2 = 63.12$, df = 16 (P < 0.0001); $I^2 = 74.6\%$									
**extract = 3**									
Omolola, 2018 (1)	42.10	0.8900	5	35.50	0.9800	5	0.7%	3.4%	6.36 [2.63; 10.10]
Omolola, 2018 (2)	47.00	1.7200	5	35.50	0.9800	5	0.5%	3.2%	7.42 [3.13; 11.70]
Omolola, 2018 (3)	48.10	4.1700	5	35.50	0.9800	5	1.6%	3.8%	3.76 [1.34; 6.17]
Onadeko, 2021 (1)	59.70	25.0800	4	51.00	14.3400	4	4.7%	4.1%	0.37 [-1.04; 1.78]
Onadeko, 2021 (2)	77.61	63.2800	4	51.00	14.3400	4	4.6%	4.1%	0.50 [-0.92; 1.93]
**Total (common effect, 95% CI)**			**23**			**23**	**12.2%**	.	**1.50 [0.62; 2.38]**
**Total (random effect, 95% CI)**							.	**18.6%**	**3.22 [0.47; 5.97]**
Heterogeneity: $Tau^2 = 7.9249$; $Chi^2 = 21.54$, df = 4 (P = 0.0002); $I^2 = 81.4\%$									
**Total (common effect, 95% CI)**			**157**			**157**	**100.0%**	.	**1.04 [0.73; 1.35]**
**Total (random effect, 95% CI)**							.	**100.0%**	**2.72 [1.21; 4.22]**
**Prediction interval**									**[-5.06; 10.49]**
Heterogeneity: $Tau^2 = 13.7175$; $Chi^2 = 162.33$, df = 26 (P < 0.0001); $I^2 = 84.0\%$									
Test for subgroup differences (common effect): $Chi^2 = 73.29$, df = 2 (P < 0.0001)									
Test for subgroup differences (random effects): $Chi^2 = 61.07$, df = 2 (P < 0.0001)									

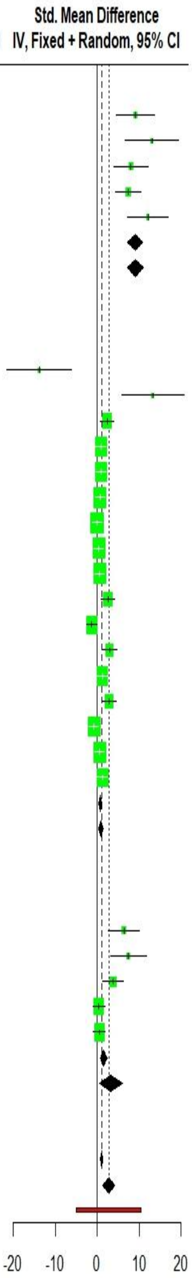

Extract 1: Aqueous
Extract 2: Methanol
Extract 3: Ethanol
