# Supplementary material for: Antinociceptive activity of Laportea species mediated by anti-inflammatory and antioxidant mechanisms: a systematic review and meta-analysis of in vivo animal studies
Source: BMC Complement Med Ther. 2026 Feb 3;26:85. doi: 10.1186/s12906-026-05262-0 (PMC12958739; doi:10.1186/s12906-026-05262-0)
Supplement: Supplementary file 11 — Supplementary Material 11. [file 12906_2026_5262_MOESM11_ESM.pdf]

ADDITIONAL FILE 11

ANTIOXIDANT: CATALASE

A. Meta Regression

Mixed-effect model (k = 25)
R^2 = 52.81%, Q_M, p < 0,0001

Variable	β	SMD [95% CI]	p - value
Laportea_sp	22.42	-105.88 [-149.84; -61.94]	<0.0001
Tissue	0,34	0.67 [-0.03; 1.37]	0,06
Duration	10.24	-55.54 [-75.60; -35.47]	<0.0001
Dose	0,64	0.26 [-1.00; 1.51]	0,69
Extract	57,61	11,21 [35,64; 79,59]	<0,0001
Method	14.63	-58.77 [-87.43; -30.10]	<0,0001
Animal_sp	0.85	0.0206 [-1.65; 1.79]	0.98

B. Forest Plot Subgroup Duration

Study or Subgroup	Experimental Mean	Experimental SD	Experimental Total	Control Mean	Control SD	Control Total	Weight (common)	Weight (random)	Std. Mean Difference IV, Fixed + Random, 95% CI
**duration = 1**									
Elizabeth, 2018 (1)	100.05	0.5800	6	66.15	1.0400	6	0.0%	0.4%	37.15 [19.21; 55.09]
Elizabeth, 2018 (2)	98.21	0.7400	6	66.15	1.0400	6	0.0%	0.5%	32.78 [16.94; 48.61]
Elizabeth, 2018 (3)	105.74	1.6000	6	66.15	1.0400	6	0.1%	0.6%	27.07 [13.98; 40.17]
Ganiyu, 2023 (1)	1.90	1.8980	5	1.76	0.0111	5	8.0%	4.9%	0.09 [-1.15; 1.33]
Ganiyu, 2023 (2)	1.82	0.0135	5	1.76	0.0111	5	1.9%	4.1%	4.02 [1.47; 6.56]
**Total (common effect, 95% CI)**			**28**			**28**	**10.1%**	.	**1.33 [0.22; 2.43]**
**Total (random effect, 95% CI)**							.	**10.5%**	**18.54 [3.36; 33.73]**
Heterogeneity: $Tau^2 = 264.7923$; $Chi^2 = 53.44$, df = 4 ($P < 0.0001$); $I^2 = 92.5\%$									
**duration = 3**									
Njina, 2016 (1)	769.77	191.0000	6	442.80	94.7500	6	5.6%	4.8%	2.00 [0.51; 3.49]
Njina, 2016 (10)	153.54	20.1500	6	111.28	24.9400	6	6.3%	4.9%	1.72 [0.32; 3.12]
Njina, 2016 (11)	153.94	37.6600	6	111.28	24.9400	6	7.6%	4.9%	1.23 [-0.05; 2.51]
Njina, 2016 (12)	149.90	35.6000	6	111.28	24.9400	6	7.8%	4.9%	1.16 [-0.10; 2.42]
Njina, 2016 (13)	218.85	8.5500	6	133.11	13.8900	6	1.0%	3.5%	6.86 [3.37; 10.35]
Njina, 2016 (14)	246.12	46.7700	6	133.11	13.8900	6	3.6%	4.6%	3.02 [1.18; 4.87]
Njina, 2016 (15)	201.84	23.6400	6	133.11	13.8900	6	3.3%	4.5%	3.27 [1.33; 5.21]
Njina, 2016 (2)	644.53	139.2400	6	442.80	94.7500	6	6.7%	4.9%	1.56 [0.20; 2.92]
Njina, 2016 (3)	652.19	110.6300	6	442.80	94.7500	6	5.9%	4.8%	1.88 [0.43; 3.32]
Njina, 2016 (4)	9.13	1.6000	6	14.91	3.2900	6	5.5%	4.8%	-2.06 [-3.57; -0.56]
Njina, 2016 (5)	5.88	0.5000	6	14.91	3.2900	6	3.0%	4.5%	-3.54 [-5.59; -1.49]
Njina, 2016 (6)	18.85	5.4500	6	14.91	3.2900	6	8.6%	5.0%	0.81 [-0.39; 2.00]
Njina, 2016 (7)	69.91	16.2100	6	50.67	11.4900	6	7.5%	4.9%	1.26 [-0.02; 2.55]
Njina, 2016 (8)	130.98	21.6600	6	50.67	11.4900	6	2.2%	4.3%	4.27 [1.92; 6.62]
Njina, 2016 (9)	103.12	20.4700	6	50.67	11.4900	6	3.8%	4.6%	2.92 [1.11; 4.72]
Omolola, 2018 (1)	22.33	1.9400	5	11.36	2.3900	5	1.6%	4.0%	4.55 [1.74; 7.35]
Omolola, 2018 (2)	25.66	2.2200	5	11.36	2.3900	5	1.1%	3.6%	5.60 [2.26; 8.93]
Omolola, 2018 (3)	29.43	1.3800	5	11.36	2.3900	5	0.5%	2.7%	8.36 [3.57; 13.14]
**Total (common effect, 95% CI)**			**105**			**105**	**81.6%**	.	**1.52 [1.13; 1.91]**
**Total (random effect, 95% CI)**							.	**80.1%**	**2.15 [1.01; 3.28]**
Heterogeneity: $Tau^2 = 4.9217$; $Chi^2 = 88.25$, df = 17 ($P < 0.0001$); $I^2 = 80.7\%$									
**duration = 2**									
Tijani, 2022 (1)	13.58	1.2400	8	8.03	2.0000	8	4.9%	4.8%	3.15 [1.57; 4.74]
Tijani, 2022 (2)	16.23	1.7500	8	8.03	2.0000	8	3.4%	4.6%	4.13 [2.22; 6.03]
**Total (common effect, 95% CI)**			**16**			**16**	**8.3%**	.	**3.55 [2.33; 4.77]**
**Total (random effect, 95% CI)**							.	**9.3%**	**3.55 [2.33; 4.77]**
Heterogeneity: $Tau^2 = 0$; $Chi^2 = 0.59$, df = 1 ($P = 0.4421$); $I^2 = 0\%$									
**Total (common effect, 95% CI)**			**149**			**149**	**100.0%**	.	**1.67 [1.32; 2.02]**
**Total (random effect, 95% CI)**							.	**100.0%**	**2.73 [1.61; 3.86]**
**Prediction interval**									**[-2.57; 8.04]**
Heterogeneity: $Tau^2 = 6.2752$; $Chi^2 = 152.38$, df = 24 ($P < 0.0001$); $I^2 = 84.2\%$									
Test for subgroup differences (common effect): $Chi^2 = 10.10$, df = 2 ($P = 0.0064$)									
Test for subgroup differences (random effects): $Chi^2 = 6.85$, df = 2 ($P = 0.0325$)									

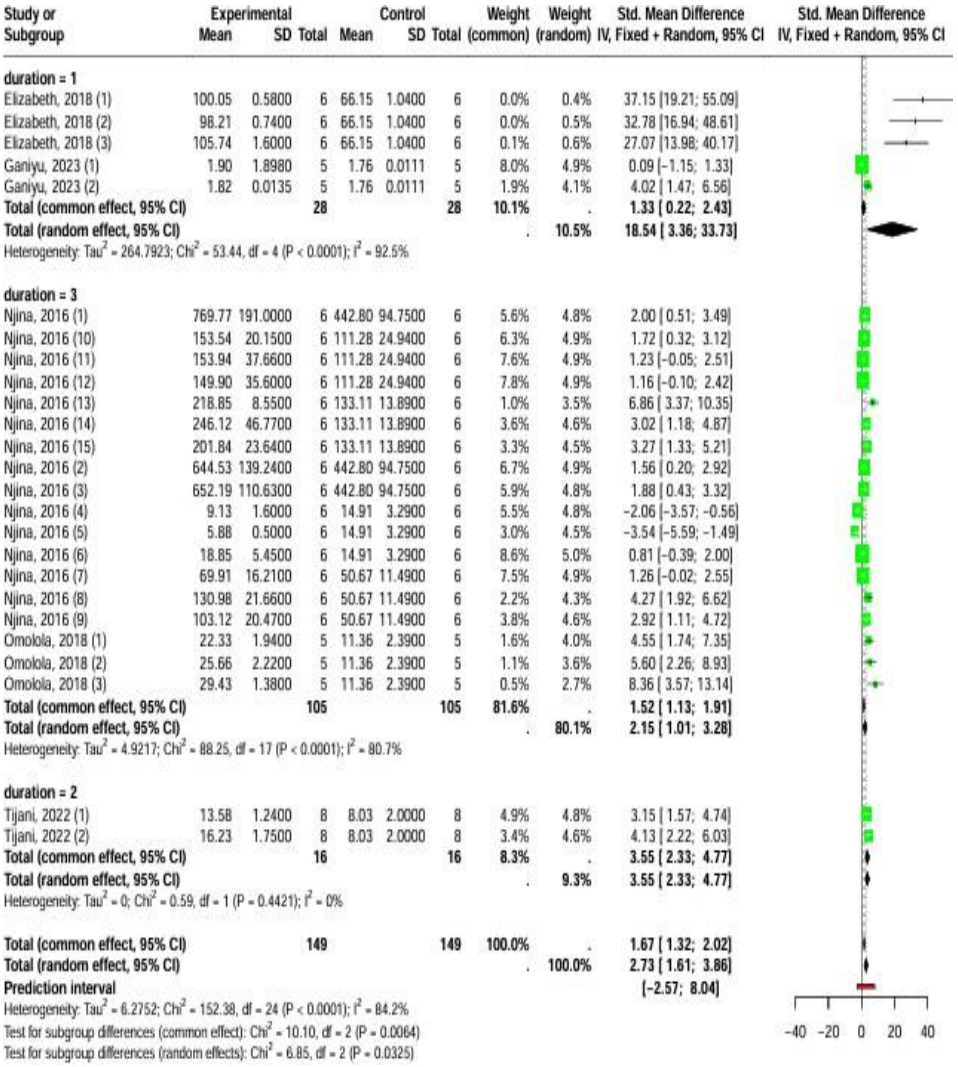

Duration 1: 1- 3 day
Duration 2: 4-7 day
Duration 3: > 7 day

C. Forest Plot Subgroup Extract

Study or Subgroup	Experimental Mean	Experimental SD	Experimental Total	Control Mean	Control SD	Control Total	Weight (common)	Weight (random)	Std. Mean Difference IV, Fixed + Random, 95% CI
**extract = 2**									
Elizabeth, 2018 (1)	100.05	0.5800	6	66.15	1.0400	6	0.0%	0.4%	37.15 [19.21; 55.09]
Elizabeth, 2018 (2)	98.21	0.7400	6	66.15	1.0400	6	0.0%	0.5%	32.78 [16.94; 48.61]
Elizabeth, 2018 (3)	105.74	1.6000	6	66.15	1.0400	6	0.1%	0.6%	27.07 [13.98; 40.17]
Tijani, 2022 (1)	13.58	1.2400	8	8.03	2.0000	8	4.9%	4.8%	3.15 [1.57; 4.74]
Tijani, 2022 (2)	16.23	1.7500	8	8.03	2.0000	8	3.4%	4.6%	4.13 [2.22; 6.03]
**Total (common effect, 95% CI)**			**34**			**34**	**8.5%**	.	**4.07 [2.87; 5.28]**
**Total (random effect, 95% CI)**							.	**10.8%**	**19.07 [4.70; 33.44]**
Heterogeneity: $Tau^2 = 233.7980$; $Chi^2 = 38.84$, df = 4 ($P < 0.0001$); $I^2 = 89.7\%$									
**extract = 1**									
Ganiyu, 2023 (1)	1.90	1.8980	5	1.76	0.0111	5	8.0%	4.9%	0.09 [-1.15; 1.33]
Ganiyu, 2023 (2)	1.82	0.0135	5	1.76	0.0111	5	1.9%	4.1%	4.02 [1.47; 6.56]
Njina, 2016 (1)	769.77	191.0000	6	442.80	94.7500	6	5.6%	4.8%	2.00 [0.51; 3.49]
Njina, 2016 (10)	153.54	20.1500	6	111.28	24.9400	6	6.3%	4.9%	1.72 [0.32; 3.12]
Njina, 2016 (11)	153.94	37.6600	6	111.28	24.9400	6	7.6%	4.9%	1.23 [-0.05; 2.51]
Njina, 2016 (12)	149.90	35.6000	6	111.28	24.9400	6	7.8%	4.9%	1.16 [-0.10; 2.42]
Njina, 2016 (13)	218.85	8.5500	6	133.11	13.8900	6	1.0%	3.5%	6.86 [3.37; 10.35]
Njina, 2016 (14)	246.12	46.7700	6	133.11	13.8900	6	3.6%	4.6%	3.02 [1.18; 4.87]
Njina, 2016 (15)	201.84	23.6400	6	133.11	13.8900	6	3.3%	4.5%	3.27 [1.33; 5.21]
Njina, 2016 (2)	644.53	139.2400	6	442.80	94.7500	6	6.7%	4.9%	1.56 [0.20; 2.92]
Njina, 2016 (3)	652.19	110.6300	6	442.80	94.7500	6	5.9%	4.8%	1.88 [0.43; 3.32]
Njina, 2016 (4)	9.13	1.6000	6	14.91	3.2900	6	5.5%	4.8%	-2.06 [-3.57; -0.56]
Njina, 2016 (5)	5.88	0.5000	6	14.91	3.2900	6	3.0%	4.5%	-3.54 [-5.59; -1.49]
Njina, 2016 (6)	18.85	5.4500	6	14.91	3.2900	6	8.6%	5.0%	0.81 [-0.39; 2.00]
Njina, 2016 (7)	69.91	16.2100	6	50.67	11.4900	6	7.5%	4.9%	1.26 [-0.02; 2.55]
Njina, 2016 (8)	130.98	21.6600	6	50.67	11.4900	6	2.2%	4.3%	4.27 [1.92; 6.62]
Njina, 2016 (9)	103.12	20.4700	6	50.67	11.4900	6	3.8%	4.6%	2.92 [1.11; 4.72]
**Total (common effect, 95% CI)**			**100**			**100**	**88.3%**	.	**1.30 [0.92; 1.67]**
**Total (random effect, 95% CI)**							.	**79.0%**	**1.62 [0.63; 2.61]**
Heterogeneity: $Tau^2 = 3.5545$; $Chi^2 = 77.6$, df = 16 ($P < 0.0001$); $I^2 = 79.4\%$									
**extract = 3**									
Omolola, 2018 (1)	22.33	1.9400	5	11.36	2.3900	5	1.6%	4.0%	4.55 [1.74; 7.35]
Omolola, 2018 (2)	25.66	2.2200	5	11.36	2.3900	5	1.1%	3.6%	5.60 [2.26; 8.93]
Omolola, 2018 (3)	29.43	1.3800	5	11.36	2.3900	5	0.5%	2.7%	8.36 [3.57; 13.14]
**Total (common effect, 95% CI)**			**15**			**15**	**3.2%**	.	**5.55 [3.59; 7.51]**
**Total (random effect, 95% CI)**							.	**10.2%**	**5.55 [3.59; 7.51]**
Heterogeneity: $Tau^2 = < 0.0001$; $Chi^2 = 1.81$, df = 2 ($P = 0.4040$); $I^2 = 0\%$									
**Total (common effect, 95% CI)**			**149**			**149**	**100.0%**	.	**1.67 [1.32; 2.02]**
**Total (random effect, 95% CI)**							.	**100.0%**	**2.73 [1.61; 3.86]**
**Prediction interval**									**[-2.57; 8.04]**

Heterogeneity: $Tau^2 = 6.2752$; $Chi^2 = 152.38$, df = 24 ($P < 0.0001$); $I^2 = 84.2\%$
Test for subgroup differences (common effect): $Chi^2 = 34.14$, df = 2 ($P < 0.0001$)
Test for subgroup differences (random effects): $Chi^2 = 17.42$, df = 2 ($P = 0.0002$)

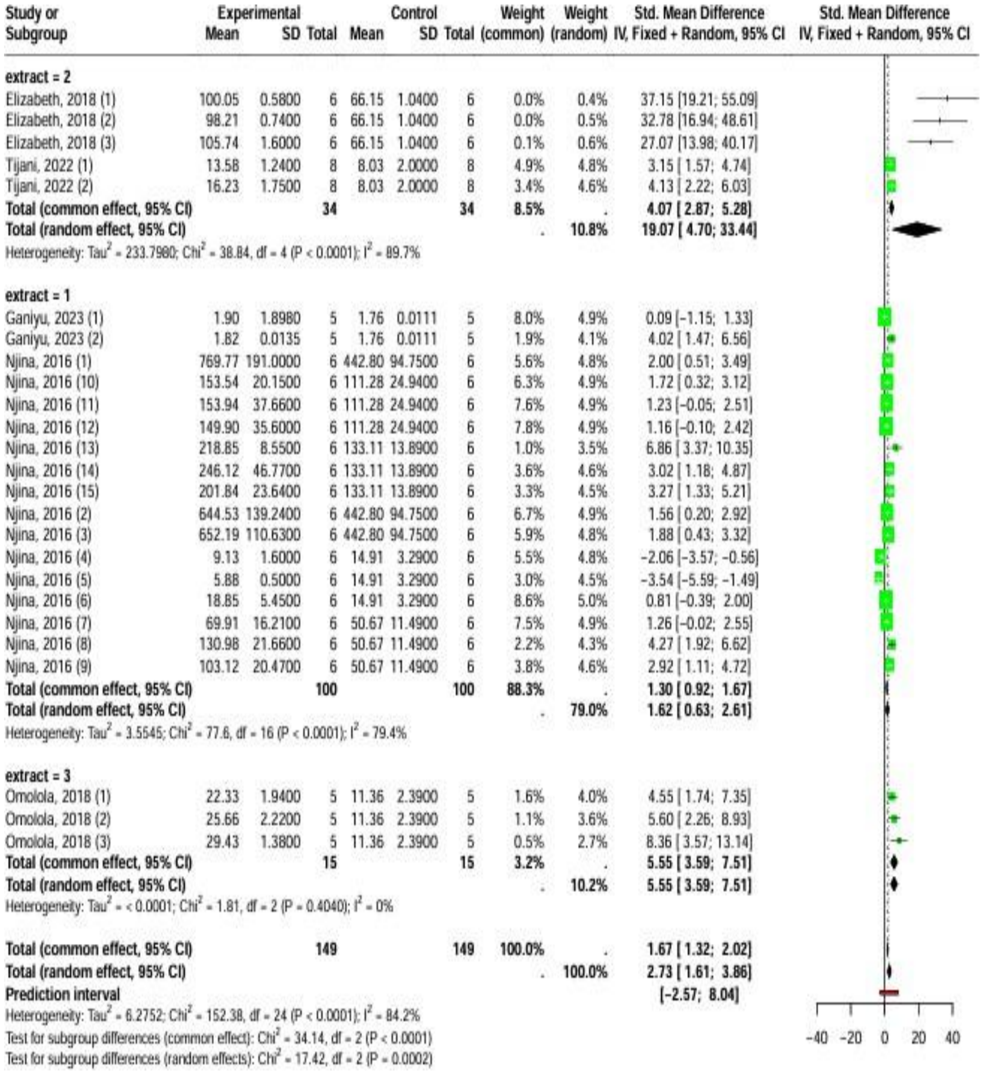

Extract 1; Aquous
Extract 2: Methanol
Extract 3: Ethanol

D. Forest Plot Subgroup Laportea species

Study or Subgroup	Experimental Mean	Experimental SD	Experimental Total	Control Mean	Control SD	Control Total	Weight (common)	Weight (random)	Std. Mean Difference IV, Fixed + Random, 95% CI
**laportea_sp = 2**									
Elizabeth, 2018 (1)	100.05	0.5800	6	66.15	1.0400	6	0.0%	0.4%	37.15 [19.21; 55.09]
Elizabeth, 2018 (2)	98.21	0.7400	6	66.15	1.0400	6	0.0%	0.5%	32.78 [16.94; 48.61]
Elizabeth, 2018 (3)	105.74	1.6000	6	66.15	1.0400	6	0.1%	0.6%	27.07 [13.98; 40.17]
Ganiyu, 2023 (1)	1.90	1.8980	5	1.76	0.0111	5	8.0%	4.9%	0.09 [-1.15; 1.33]
Ganiyu, 2023 (2)	1.82	0.0135	5	1.76	0.0111	5	1.9%	4.1%	4.02 [1.47; 6.56]
Omolola, 2018 (1)	22.33	1.9400	5	11.36	2.3900	5	1.6%	4.0%	4.55 [1.74; 7.35]
Omolola, 2018 (2)	25.66	2.2200	5	11.36	2.3900	5	1.1%	3.6%	5.60 [2.26; 8.93]
Omolola, 2018 (3)	29.43	1.3800	5	11.36	2.3900	5	0.5%	2.7%	8.36 [3.57; 13.14]
Tijani, 2022 (1)	13.58	1.2400	8	8.03	2.0000	8	4.9%	4.8%	3.15 [1.57; 4.74]
Tijani, 2022 (2)	16.23	1.7500	8	8.03	2.0000	8	3.4%	4.6%	4.13 [2.22; 6.03]
**Total (common effect, 95% CI)**			**59**			**59**	**21.7%**	.	**2.81 [2.06; 3.57]**
**Total (random effect, 95% CI)**							.	**30.1%**	**10.23 [3.34; 17.12]**
Heterogeneity: $Tau^2 = 108.2495$; $Chi^2 = 71.68$, df = 9 ($P < 0.0001$); $I^2 = 87.4\%$									
**laportea_sp = 1**									
Njina, 2016 (1)	769.77	191.0000	6	442.80	94.7500	6	5.6%	4.8%	2.00 [0.51; 3.49]
Njina, 2016 (10)	153.54	20.1500	6	111.28	24.9400	6	6.3%	4.9%	1.72 [0.32; 3.12]
Njina, 2016 (11)	153.94	37.6600	6	111.28	24.9400	6	7.6%	4.9%	1.23 [-0.05; 2.51]
Njina, 2016 (12)	149.90	35.6000	6	111.28	24.9400	6	7.8%	4.9%	1.16 [-0.10; 2.42]
Njina, 2016 (13)	218.85	8.5500	6	133.11	13.8900	6	1.0%	3.5%	6.86 [3.37; 10.35]
Njina, 2016 (14)	246.12	46.7700	6	133.11	13.8900	6	3.6%	4.6%	3.02 [1.18; 4.87]
Njina, 2016 (15)	201.84	23.6400	6	133.11	13.8900	6	3.3%	4.5%	3.27 [1.33; 5.21]
Njina, 2016 (2)	644.53	139.2400	6	442.80	94.7500	6	6.7%	4.9%	1.56 [0.20; 2.92]
Njina, 2016 (3)	652.19	110.6300	6	442.80	94.7500	6	5.9%	4.8%	1.88 [0.43; 3.32]
Njina, 2016 (4)	9.13	1.6000	6	14.91	3.2900	6	5.5%	4.8%	-2.06 [-3.57; -0.56]
Njina, 2016 (5)	5.88	0.5000	6	14.91	3.2900	6	3.0%	4.5%	-3.54 [-5.59; -1.49]
Njina, 2016 (6)	18.85	5.4500	6	14.91	3.2900	6	8.6%	5.0%	0.81 [-0.39; 2.00]
Njina, 2016 (7)	69.91	16.2100	6	50.67	11.4900	6	7.5%	4.9%	1.26 [-0.02; 2.55]
Njina, 2016 (8)	130.98	21.6600	6	50.67	11.4900	6	2.2%	4.3%	4.27 [1.92; 6.62]
Njina, 2016 (9)	103.12	20.4700	6	50.67	11.4900	6	3.8%	4.6%	2.92 [1.11; 4.72]
**Total (common effect, 95% CI)**			**90**			**90**	**78.3%**	.	**1.35 [0.96; 1.75]**
**Total (random effect, 95% CI)**							.	**69.9%**	**1.61 [0.54; 2.68]**
Heterogeneity: $Tau^2 = 3.6925$; $Chi^2 = 69.5$, df = 14 ($P < 0.0001$); $I^2 = 79.9\%$									
**Total (common effect, 95% CI)**			**149**			**149**	**100.0%**	.	**1.67 [1.32; 2.02]**
**Total (random effect, 95% CI)**							.	**100.0%**	**2.73 [1.61; 3.86]**
**Prediction interval**									**[-2.57; 8.04]**

Heterogeneity: $Tau^2 = 6.2752$; $Chi^2 = 152.38$, df = 24 ($P < 0.0001$); $I^2 = 84.2\%$

Test for subgroup differences (common effect): $Chi^2 = 11.20$, df = 1 ($P = 0.0008$)

Test for subgroup differences (random effects): $Chi^2 = 5.88$, df = 1 ($P = 0.0153$)

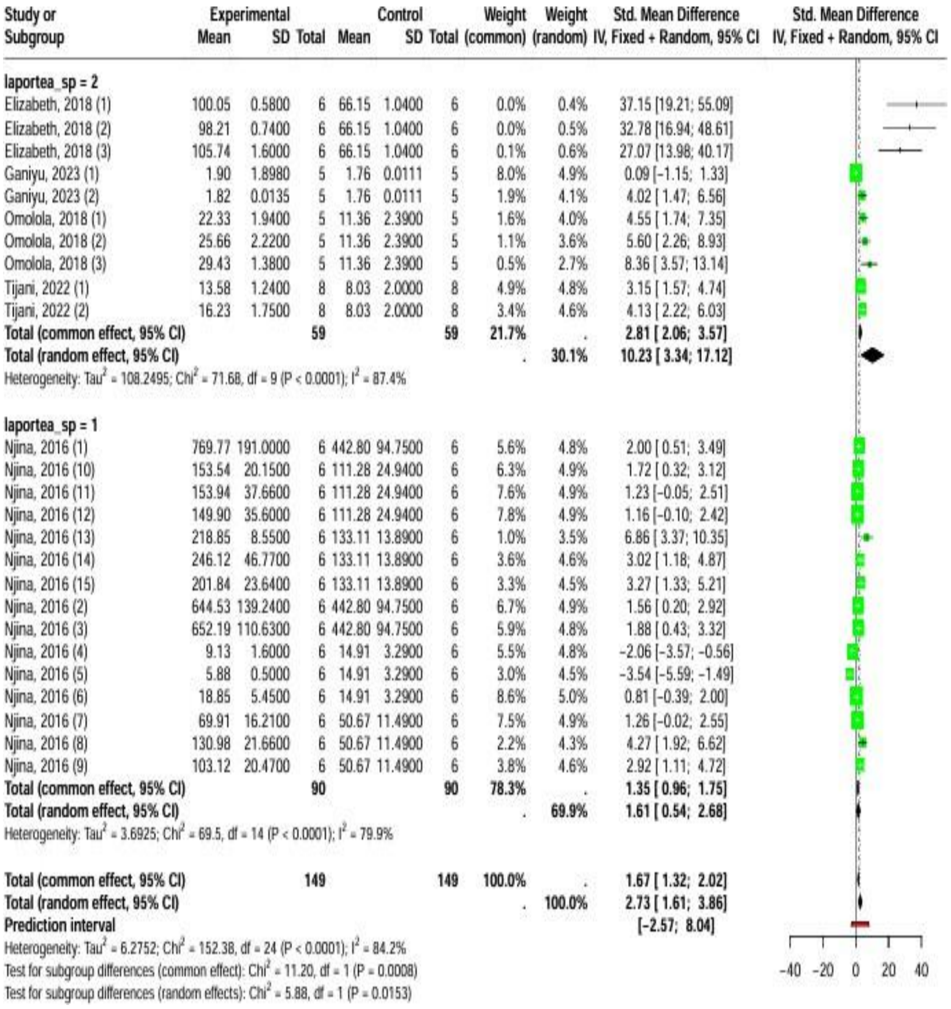

Laportea 1: L. ovalifolia
Laportea 2: L. aestuans

E. Subgroup Method

Study or Subgroup	Experimental Mean	Experimental SD	Experimental Total	Control Mean	Control SD	Control Total	Weight (common)	Weight (random)	Std. Mean Difference IV, Fixed + Random, 95% CI
**method = 2**									
Elizabeth, 2018 (1)	100.05	0.5800	6	66.15	1.0400	6	0.0%	0.4%	37.15 [19.21; 55.09]
Elizabeth, 2018 (2)	98.21	0.7400	6	66.15	1.0400	6	0.0%	0.5%	32.78 [16.94; 48.61]
Elizabeth, 2018 (3)	105.74	1.6000	6	66.15	1.0400	6	0.1%	0.6%	27.07 [13.98; 40.17]
**Total (common effect, 95% CI)**			**18**			**18**	**0.2%**	.	**31.26 [22.46; 40.05]**
**Total (random effect, 95% CI)**							.	**1.5%**	**31.26 [22.46; 40.05]**
Heterogeneity: Tau^2 = 0; Chi^2 = 0.84, df = 2 (P = 0.6563); I^2 = 0%									
**method = 4**									
Ganiyu, 2023 (1)	1.90	1.8980	5	1.76	0.0111	5	8.0%	4.9%	0.09 [-1.15; 1.33]
Ganiyu, 2023 (2)	1.82	0.0135	5	1.76	0.0111	5	1.9%	4.1%	4.02 [1.47; 6.56]
**Total (common effect, 95% CI)**			**10**			**10**	**9.9%**	.	**0.85 [-0.27; 1.96]**
**Total (random effect, 95% CI)**							.	**9.1%**	**1.89 [-1.94; 5.72]**
Heterogeneity: Tau^2 = 6.6637; Chi^2 = 7.39, df = 1 (P = 0.0066); I^2 = 86.5%									
**method = 1**									
Njina, 2016 (1)	769.77	191.0000	6	442.80	94.7500	6	5.6%	4.8%	2.00 [0.51; 3.49]
Njina, 2016 (10)	153.54	20.1500	6	111.28	24.9400	6	6.3%	4.9%	1.72 [0.32; 3.12]
Njina, 2016 (11)	153.94	37.6600	6	111.28	24.9400	6	7.6%	4.9%	1.23 [-0.05; 2.51]
Njina, 2016 (12)	149.90	35.6000	6	111.28	24.9400	6	7.8%	4.9%	1.16 [-0.10; 2.42]
Njina, 2016 (13)	218.85	8.5500	6	133.11	13.8900	6	1.0%	3.5%	6.86 [3.37; 10.35]
Njina, 2016 (14)	246.12	46.7700	6	133.11	13.8900	6	3.6%	4.6%	3.02 [1.18; 4.87]
Njina, 2016 (15)	201.84	23.6400	6	133.11	13.8900	6	3.3%	4.5%	3.27 [1.33; 5.21]
Njina, 2016 (2)	644.53	139.2400	6	442.80	94.7500	6	6.7%	4.9%	1.56 [0.20; 2.92]
Njina, 2016 (3)	652.19	110.6300	6	442.80	94.7500	6	5.9%	4.8%	1.88 [0.43; 3.32]
Njina, 2016 (4)	9.13	1.6000	6	14.91	3.2900	6	5.5%	4.8%	-2.06 [-3.57; -0.56]
Njina, 2016 (5)	5.88	0.5000	6	14.91	3.2900	6	3.0%	4.5%	-3.54 [-5.59; -1.49]
Njina, 2016 (6)	18.85	5.4500	6	14.91	3.2900	6	8.6%	5.0%	0.81 [-0.39; 2.00]
Njina, 2016 (7)	69.91	16.2100	6	50.67	11.4900	6	7.5%	4.9%	1.26 [-0.02; 2.55]
Njina, 2016 (8)	130.98	21.6600	6	50.67	11.4900	6	2.2%	4.3%	4.27 [1.92; 6.62]
Njina, 2016 (9)	103.12	20.4700	6	50.67	11.4900	6	3.8%	4.6%	2.92 [1.11; 4.72]
**Total (common effect, 95% CI)**			**90**			**90**	**78.3%**	.	**1.35 [0.96; 1.75]**
**Total (random effect, 95% CI)**							.	**69.9%**	**1.61 [0.54; 2.68]**
Heterogeneity: Tau^2 = 3.6925; Chi^2 = 69.5, df = 14 (P < 0.0001); I^2 = 79.9%									
**method = 5**									
Omolola, 2018 (1)	22.33	1.9400	5	11.36	2.3900	5	1.6%	4.0%	4.55 [1.74; 7.35]
Omolola, 2018 (2)	25.66	2.2200	5	11.36	2.3900	5	1.1%	3.6%	5.60 [2.26; 8.93]
Omolola, 2018 (3)	29.43	1.3800	5	11.36	2.3900	5	0.5%	2.7%	8.36 [3.57; 13.14]
**Total (common effect, 95% CI)**			**15**			**15**	**3.2%**	.	**5.55 [3.59; 7.51]**
**Total (random effect, 95% CI)**							.	**10.2%**	**5.55 [3.59; 7.51]**
Heterogeneity: Tau^2 = < 0.0001; Chi^2 = 1.81, df = 2 (P = 0.4040); I^2 = 0%									
**method = 3**									
Tijani, 2022 (1)	13.58	1.2400	8	8.03	2.0000	8	4.9%	4.8%	3.15 [1.57; 4.74]
Tijani, 2022 (2)	16.23	1.7500	8	8.03	2.0000	8	3.4%	4.6%	4.13 [2.22; 6.03]
**Total (common effect, 95% CI)**			**16**			**16**	**8.3%**	.	**3.55 [2.33; 4.77]**
**Total (random effect, 95% CI)**							.	**9.3%**	**3.55 [2.33; 4.77]**
Heterogeneity: Tau^2 = 0, Chi^2 = 0.59, df = 1 (P = 0.4421); I^2 = 0%									
**Total (common effect, 95% CI)**			**149**			**149**	**100.0%**	.	**1.67 [1.32; 2.02]**
**Total (random effect, 95% CI)**							.	**100.0%**	**2.73 [1.61; 3.86]**
**Prediction interval**									**[-2.57; 8.04]**

Heterogeneity: Tau^2 = 6.2752; Chi^2 = 152.38, df = 24 (P < 0.0001); I^2 = 84.2%
Test for subgroup differences (common effect): Chi^2 = 72.24, df = 4 (P < 0.0001)
Test for subgroup differences (random effects): Chi^2 = 53.77, df = 4 (P < 0.0001)

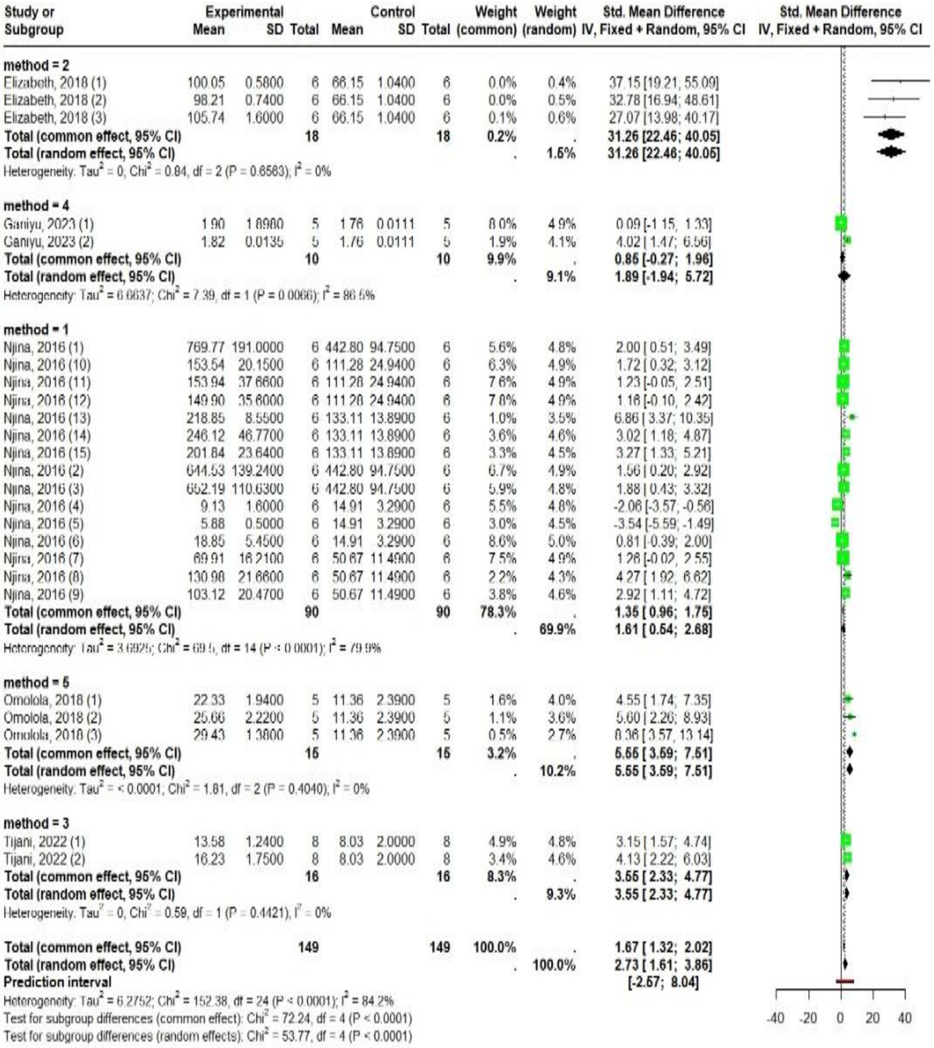

Method 1: castrated rat
Method 2: Diclofenac induced rat
Method 3: ulcer model
Method 4: liver injury
Method 5: BPH
