# Supplementary material for: Antinociceptive activity of Laportea species mediated by anti-inflammatory and antioxidant mechanisms: a systematic review and meta-analysis of in vivo animal studies
Source: BMC Complement Med Ther. 2026 Feb 3;26:85. doi: 10.1186/s12906-026-05262-0 (PMC12958739; doi:10.1186/s12906-026-05262-0)
Supplement: Supplementary file 12 — Supplementary Material 12. [file 12906_2026_5262_MOESM12_ESM.pdf]

ADDITIONAL FILE 12

Antioxidant: GSH

A. Meta regression

Mixed-effect models (k = 8)
R^2 = 64.34%; Q_M, p = 0.005)

Variable	β	SMD [95% CI]	p-value
Dose	3.81	0.97 [-6.49; 8.44]	0.79
Tissue	1.79	5.88 [2.36; 9.36]	0.001
Duration	5.67	-14.17 [-25.28; -3.06]	0.012
Extract	10.82	31.81 [10.61; 53.01]	0.003
Method	4.09	-8.29 [16.30; -0.28]	0.042

B. Subgroup Tissue

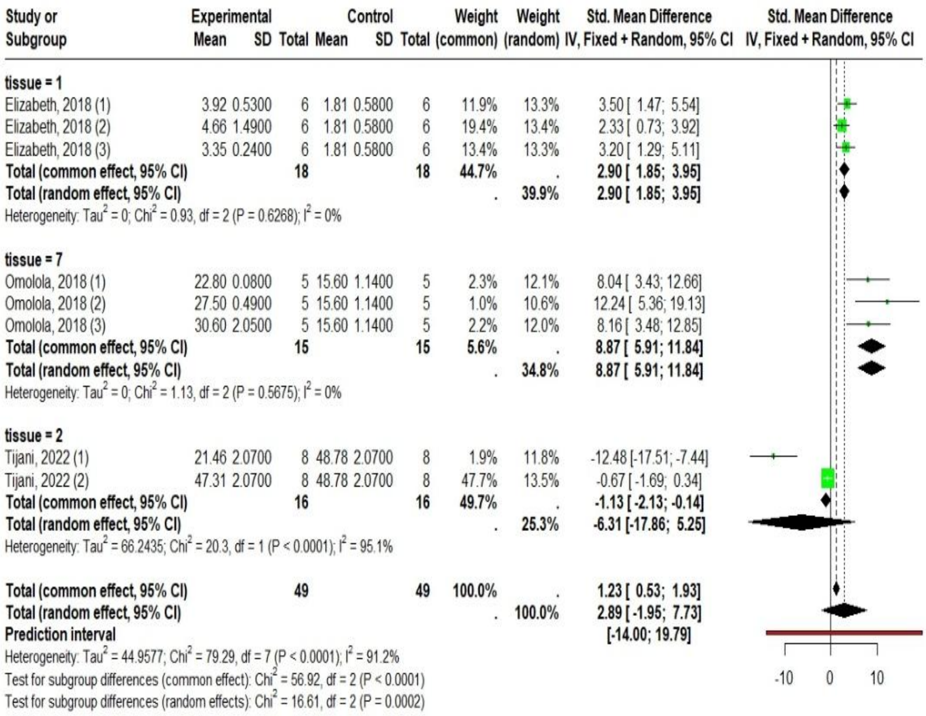

Tissue 1: brain
Tissue 2: gaster
Tissue 7: serum

c. Subgroup Duration

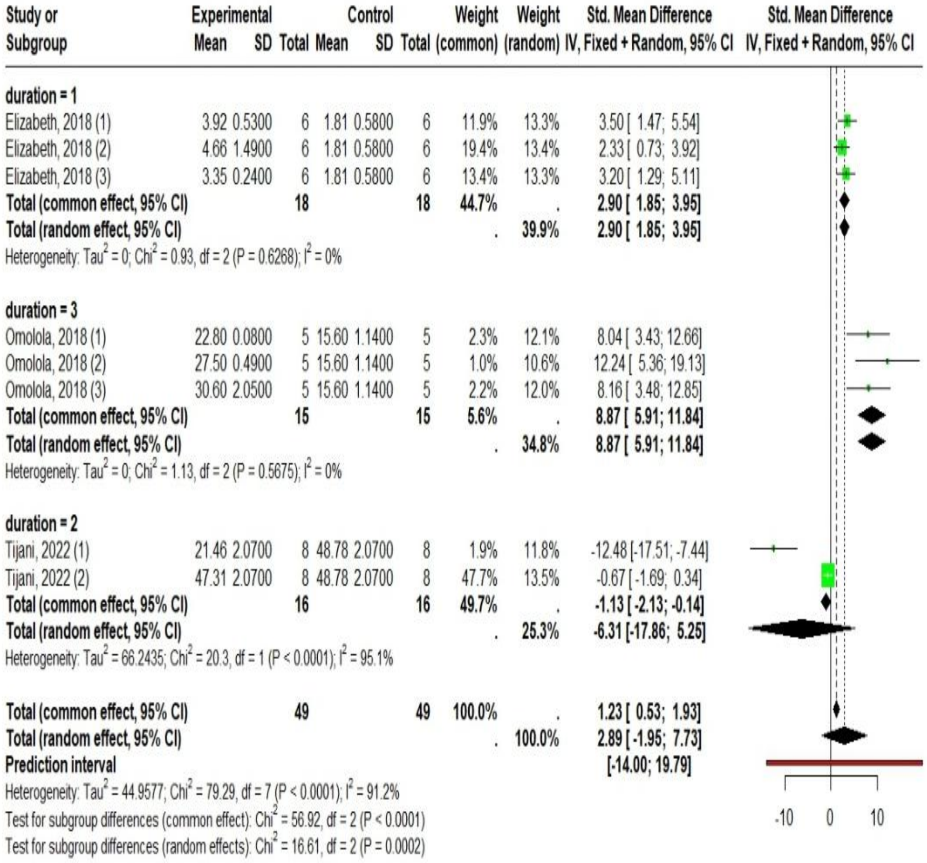

Duration 1: 1- 3 days
Duration 2: 4 – 7 days
Duration 3: > 7 days

D.Subgroup Extract

Study or Subgroup	Experimental Mean	SD	Total	Control Mean	SD	Total	Weight (common)	Weight (random)	Std. Mean Difference IV, Fixed + Random, 95% CI
**extract = 2**									
Elizabeth, 2018 (1)	3.92	0.5300	6	1.81	0.5800	6	11.9%	13.3%	3.50 [1.47; 5.54]
Elizabeth, 2018 (2)	4.66	1.4900	6	1.81	0.5800	6	19.4%	13.4%	2.33 [0.73; 3.92]
Elizabeth, 2018 (3)	3.35	0.2400	6	1.81	0.5800	6	13.4%	13.3%	3.20 [1.29; 5.11]
Tijani, 2022 (1)	21.46	2.0700	8	48.78	2.0700	8	1.9%	11.8%	-12.48 [-17.51; -7.44]
Tijani, 2022 (2)	47.31	2.0700	8	48.78	2.0700	8	47.7%	13.5%	-0.67 [-1.69; 0.34]
**Total (common effect, 95% CI)**			**34**			**34**	**94.4%**	.	**0.78 [0.06; 1.50]**
**Total (random effect, 95% CI)**							.	**65.2%**	**-0.51 [-5.92; 4.91]**
Heterogeneity: $Tau^2 = 36.3938$; $Chi^2 = 51.17$, df = 4 ($P < 0.0001$); $I^2 = 92.2\%$									
**extract = 3**									
Omolola, 2018 (1)	22.80	0.0800	5	15.60	1.1400	5	2.3%	12.1%	8.04 [3.43; 12.66]
Omolola, 2018 (2)	27.50	0.4900	5	15.60	1.1400	5	1.0%	10.6%	12.24 [5.36; 19.13]
Omolola, 2018 (3)	30.60	2.0500	5	15.60	1.1400	5	2.2%	12.0%	8.16 [3.48; 12.85]
**Total (common effect, 95% CI)**			**15**			**15**	**5.6%**	.	**8.87 [5.91; 11.84]**
**Total (random effect, 95% CI)**							.	**34.8%**	**8.87 [5.91; 11.84]**
Heterogeneity: $Tau^2 = 0$; $Chi^2 = 1.13$, df = 2 ($P = 0.5675$); $I^2 = 0\%$									
**Total (common effect, 95% CI)**			**49**			**49**	**100.0%**	.	**1.23 [0.53; 1.93]**
**Total (random effect, 95% CI)**							.	**100.0%**	**2.89 [-1.95; 7.73]**
**Prediction interval**									**[-14.00; 19.79]**
Heterogeneity: $Tau^2 = 44.9577$; $Chi^2 = 79.29$, df = 7 ($P < 0.0001$); $I^2 = 91.2\%$									
Test for subgroup differences (common effect): $Chi^2 = 26.98$, df = 1 ($P < 0.0001$)									
Test for subgroup differences (random effects): $Chi^2 = 8.86$, df = 1 ($P = 0.0029$)									

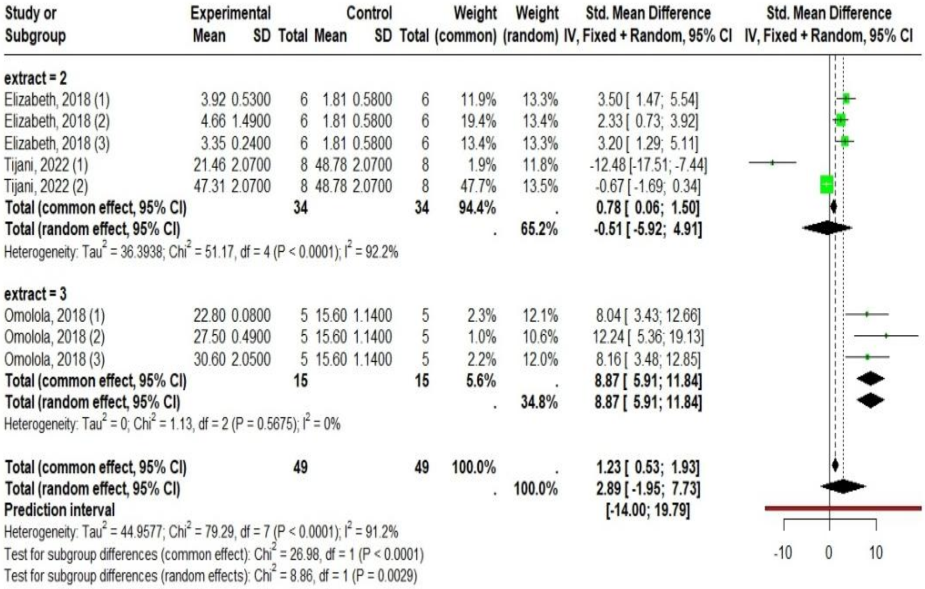

Extract 2: methanol
Extract 3: ethanol

E. Subgroup Method

Study or Subgroup	Experimental Mean	Experimental SD	Experimental Total	Control Mean	Control SD	Control Total	Weight (common)	Weight (random)	Std. Mean Difference IV, Fixed + Random, 95% CI
**method = 2**									
Elizabeth, 2018 (1)	3.92	0.5300	6	1.81	0.5800	6	11.9%	13.3%	3.50 [1.47; 5.54]
Elizabeth, 2018 (2)	4.66	1.4900	6	1.81	0.5800	6	19.4%	13.4%	2.33 [0.73; 3.92]
Elizabeth, 2018 (3)	3.35	0.2400	6	1.81	0.5800	6	13.4%	13.3%	3.20 [1.29; 5.11]
**Total (common effect, 95% CI)**			**18**			**18**	**44.7%**	.	**2.90 [1.85; 3.95]**
**Total (random effect, 95% CI)**							.	**39.9%**	**2.90 [1.85; 3.95]**
Heterogeneity: $Tau^2 = 0$; $Chi^2 = 0.93$, df = 2 (P = 0.6268); $I^2 = 0\%$									
**method = 5**									
Omolola, 2018 (1)	22.80	0.0800	5	15.60	1.1400	5	2.3%	12.1%	8.04 [3.43; 12.66]
Omolola, 2018 (2)	27.50	0.4900	5	15.60	1.1400	5	1.0%	10.6%	12.24 [5.36; 19.13]
Omolola, 2018 (3)	30.60	2.0500	5	15.60	1.1400	5	2.2%	12.0%	8.16 [3.48; 12.85]
**Total (common effect, 95% CI)**			**15**			**15**	**5.6%**	.	**8.87 [5.91; 11.84]**
**Total (random effect, 95% CI)**							.	**34.8%**	**8.87 [5.91; 11.84]**
Heterogeneity: $Tau^2 = 0$; $Chi^2 = 1.13$, df = 2 (P = 0.5675); $I^2 = 0\%$									
**method = 3**									
Tijani, 2022 (1)	21.46	2.0700	8	48.78	2.0700	8	1.9%	11.8%	-12.48 [-17.51; -7.44]
Tijani, 2022 (2)	47.31	2.0700	8	48.78	2.0700	8	47.7%	13.5%	-0.67 [-1.69; 0.34]
**Total (common effect, 95% CI)**			**16**			**16**	**49.7%**	.	**-1.13 [-2.13; -0.14]**
**Total (random effect, 95% CI)**							.	**25.3%**	**-6.31 [-17.86; 5.25]**
Heterogeneity: $Tau^2 = 66.2435$; $Chi^2 = 20.3$, df = 1 (P < 0.0001); $I^2 = 95.1\%$									
**Total (common effect, 95% CI)**			**49**			**49**	**100.0%**	.	**1.23 [0.53; 1.93]**
**Total (random effect, 95% CI)**							.	**100.0%**	**2.89 [-1.95; 7.73]**
**Prediction interval**									**[-14.00; 19.79]**

Heterogeneity: $Tau^2 = 44.9577$; $Chi^2 = 79.29$, df = 7 (P < 0.0001); $I^2 = 91.2\%$
Test for subgroup differences (common effect): $Chi^2 = 56.92$, df = 2 (P < 0.0001)
Test for subgroup differences (random effects): $Chi^2 = 16.61$, df = 2 (P = 0.0002)

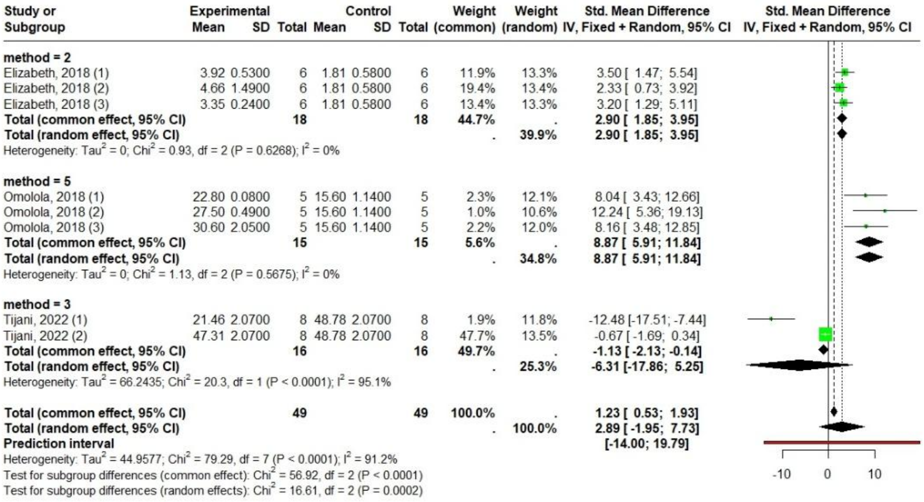

Method 2:Diclofenac induced rat
Method 3:Ulcer method
Method 5: BPH rat
