# Supplementary material for: Antinociceptive activity of Laportea species mediated by anti-inflammatory and antioxidant mechanisms: a systematic review and meta-analysis of in vivo animal studies
Source: BMC Complement Med Ther. 2026 Feb 3;26:85. doi: 10.1186/s12906-026-05262-0 (PMC12958739; doi:10.1186/s12906-026-05262-0)
Supplement: Supplementary file 13 — Supplementary Material 13. [file 12906_2026_5262_MOESM13_ESM.pdf]

ADDITIONAL FILE 13

Antioxidant: Peroxidase

A. Meta Regression

Mixed-effects model: (k = 15)
R^2 = 0,00%; Q_M, p = 0.71

Variabel	β	SMD (95% CI)	p- value
dose	0,006	0,4 [-0,79; 0,81]	0,98
tissue	-0,193	0,23 [-0,64; 0,26]	0,40
