# Supplementary material for: Antinociceptive activity of Laportea species mediated by anti-inflammatory and antioxidant mechanisms: a systematic review and meta-analysis of in vivo animal studies
Source: BMC Complement Med Ther. 2026 Feb 3;26:85. doi: 10.1186/s12906-026-05262-0 (PMC12958739; doi:10.1186/s12906-026-05262-0)
Supplement: Supplementary file 14 — Supplementary Material 14. [file 12906_2026_5262_MOESM14_ESM.pdf]

ADDITIONAL FILE 14

Antioxidant: Glutathione peroxidase

A. Meta regression

Mixed-effects models (k = 4)

R^2 = 0.00%; Q_M, p = 0.55

Variabel	β	SMD [95% CI]	pval
Tissue	23.623	39.44[-53.68; 100.953]	0.5493
Dose	-23.623	39.44[-100.93; 53;687]	0.5493
Extract	-23.623	39.44[-53.68; 100.953]	0.5493
Method	23.623	39.44[-53.68; 100.953]	0.5493
Animal species	11.811	19.77[-26.84; 50.47]	0.5493
Duration	-23.623	39.44[-100.93; 53;687]	0.5493
