# Supplementary material for: Antinociceptive activity of Laportea species mediated by anti-inflammatory and antioxidant mechanisms: a systematic review and meta-analysis of in vivo animal studies
Source: BMC Complement Med Ther. 2026 Feb 3;26:85. doi: 10.1186/s12906-026-05262-0 (PMC12958739; doi:10.1186/s12906-026-05262-0)
Supplement: Supplementary file 15 — Supplementary Material 15. [file 12906_2026_5262_MOESM15_ESM.pdf]

ADDITIONAL FILE 15
PUBLICATION BIAS

A. ANTIOXIDANT: SUPEROXIDE DISMUTASE

Funnel Plot

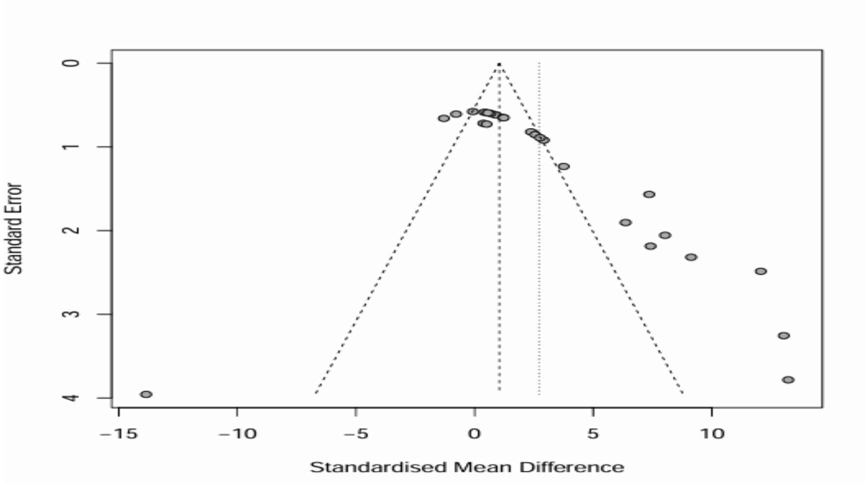

Egger's Test
Test result: t = 4.98, df = 25, p-value < 0.0001 Bias estimate: 3.9723 (SE = 0.7983)

B. ANTIOXIDANT: CATALASE

Funnel Plot

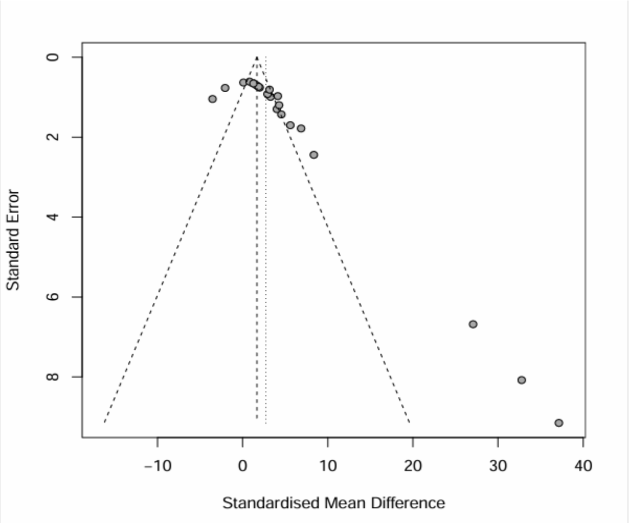

Egger's Test
Test result: t = 5.18, df = 23, p-value < 0.0001
Bias estimate: 4.2981 (SE = 0.8303)

c. Antioxidant: Peroxidase

Funnel Plot

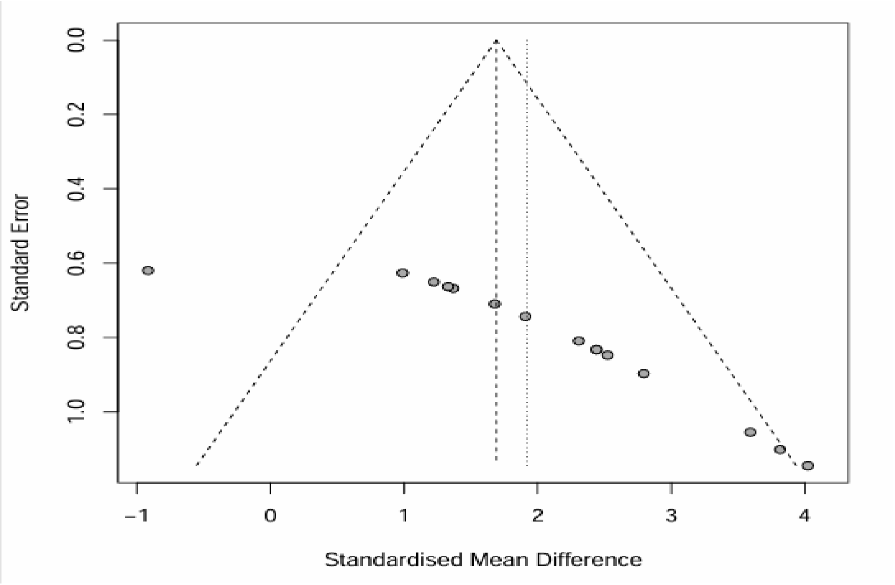

Egger's Test

Test result: t = 6.43, df = 13, p-value < 0.0001

Bias estimate: 7.3119 (SE = 1.1376)
