# Supplementary material for: Antinociceptive activity of Laportea species mediated by anti-inflammatory and antioxidant mechanisms: a systematic review and meta-analysis of in vivo animal studies
Source: BMC Complement Med Ther. 2026 Feb 3;26:85. doi: 10.1186/s12906-026-05262-0 (PMC12958739; doi:10.1186/s12906-026-05262-0)
Supplement: Supplementary file 16 — Supplementary Material 16. [file 12906_2026_5262_MOESM16_ESM.pdf]

Additional File 16

Table 2. Meta-analysis and meta regression result of *Laportea* species extract in *in-vivo* research

Pharmacological Effect / Outcome	SMD [95% CI]	p	I^2; p	Subgroup differences	Subgroup Meta-Regression highlights	
				(p)	Variables	SMD (95% CI)
Oral Antinociceptive						
1. Writhing	-6.50 [-8.05; 4.96]	< 0.0001	58.3%; 0.007	-	No significant moderators (p = 0.20)	
2. PRT	0.95 [0.48; 1.42]	0.0001	71.1%; 0.0001	-	1. extract type	
					a. methanol	10.37 [4.95; 15.78]
					b. ethanol	0.76 [0.34; 1.18]
					2. method	
					a. Tail immersion	10.37 [4.95; 15.78]
					b.hot plate test	0.76 [0.34; 1.18]
					3. tissue / organ	
					a. tail	10.37 [4.95; 15.78]
					b. paw	0.76 [0.34; 1.18]
Topical Analgesic	-2.28 [-3.38; 1.17]	< 0.001	62.2%; 0.0015	-	1. extract type	
					a. crude	-3.71 [-5.1; -2.32]
					b. ethanol	-1.66 [-2.87; -0.46]
					2. topical formulation	
					a. patch	-3.71 [-5.1; -2.32]
					b. cream	-1.66 [-2.87; -0.46]
					3. animal species	
					a. *Mus musculus* (mice)	-3.71 [-5.1; -2.32]
					b. *Rattus norvegicus* (rat)	-1.66 [-2.87; -0.46]
Oral Anti-inflammatory						
1. Pro – inflammatory	-2.62 [-3.99; -1.24]	< 0.0002	82.5%; 0.0001	0.0001		
a.IL-1β	-8.77 [-12.43; -5.10]	< 0.0001	47.7%; 0.17		Low heterogeneity	
b.TNF-α	-4.63 [-8.72; -0.53]	0.026	84.4%; 0.0115		Dosage	
					a.400 mg/kgBW	-6.92 [-9.83; -4.02]
					b.200 mg/kgBW	-2.73 [-4.18; -1.27]
c.IFN-γ	-1.28 [-1.80; -0.76]	< 0.0001	0%; 0.43		Low heterogeneity	
d.IL-2	-0.75 [-1.23; -0.26]	0.0025	0%; 0.54		Low heterogeneity	
e.oedema	-2.11 [-4.27; 0.04]	0.05	80.3%; 0.0001		no significant result	
2. Anti-inflammatory	0.49 [0.15; 0.83]	< 0.0043	46.8%; 0.09	0.86	Low heterogeneity	
a.TGF-β	0.55 [-0.30; 1.40]	0.2052	68.2%; 0.043		Homogenous data	
b.IL-10	0.46 [-0.01; 0.93]	0.0559	35.1%; 0.214		Low heterogeneity	
Topical Anti-inflammatory	-1.10 [-2.16; -0.04]	< 0.0043	62.3%; 0.02	0.82		
a.IL-6	-1.06 [-2.31; 0.19]	0.0968	53.6%; 0.12		Low heterogenity	
b.paw oedema	-1.37 [-3.83; 1.07]	0.2707	77.2%; 0.0125		Dosage	
					a.2 %	-4.40 [-7.13; -1.67]
					b.0.5 %	-0.67 [-1.96; 0.63]
					c.1 %	0.13 [-1.11; 1.37]
Oxidative-stress effect						
1.Cell Damage	-2.42 [-3.17; -1.68]	< 0.0001	77.7%; 0.0001	0.49		
a.PCO	-2.47 [-3.426; -1.52]	<0.0001	0%; 0.80		Low heterogeneity	
b.NO	-2.12 [-3.18; -1.063]	<0.0001	81.4%; 0.0001		tissue / organ	
					1.liver	-5.50 [-7.71; -3.29]
					2.kidney	-2.28 [-3.21; -1.36]
					3.heart	-2.11 [-3.21; -1.01]
c.MDA	-3.179 [4.56; -1.79]	<0.0001	69%; 0.0036		extract type	
					1.methanol	-3.94 [-5.05; -2.82]
					2.ethanol	-1.10 [-2.22; 0.01]

Table 2. Meta-analysis and meta regression result of *Laportea* species extract in *in-vivo* research (continued)						
Pharmacological Effect / Outcome	SMD [95% CI]	p	I^2; p	Subgroup differences	Subgroup Meta-Regression highlights	
				(p)	Variables	SMD (95% CI)
2. Antioxidant	2.77 [2.11; 3.42]	<0.0001	83.2%; 0.0001	0.05		
a.GSH	2.89 [-1.94; 7.73]	0.24	91.2%; 0.0001		1.tissue used	
					a.serum	8.87 [5.91; 11.84]
					b.brain	2.90 [1.85;3.95]
					2. duration treatment	
					a.>7 days	8.87 [5.91; 11.84]
					b.1-3 days	2.90 [1.85;3.95]
					3.extraction type	
					a.ethanol	8.87 [5.91; 11.84]
					b.methanol	-0.51 [-5.92; 4.91]
					4.studies methods	
					a.rat with BPH	8.87 [5.91; 11.84]
					b. Diclofenac induced rat	2.90 [1.85;3.95]
b.GPx	4.38 [1.08; 7.68]	0.01	86.8%; 0.0001		no variables significant	
c.SOD	2.72 [1.21; 4.22]	0.0004	84%; 0.0001		1. *Laportea* species	
					a. *L.aestuans*	5.63 [1.96; 9.32]
					b. *L.ovalifolia*	0.87 [0.3; 1.45]
					2. method	
					a. Aspirin-induced ulcerative model	9.33 [6.61; 12.06]
					b.BPH models	5.34 [2.99; 7.69]
					c.Castarated rat model	0.87 [0.29; 1.45]
					3. treatment duration	
					a.4- 7 days	9.31 [4.76; 13.86]
					b.> 7 days	1.30 [0.58; 2.01]
					4. extraction type	
					a.methanol	9.00 [7.09; 11.05]
					b.ethanol	3.22 [0.47; 5.97]
					c.aqueous	0.88 [0.25; 1.51]
4.CAT	2.73 [1.61; 3.86]	<0.0001	84.2%; 0.0001		1. treatment duration	
					a.1 - 3 days	18.54 [3.36; 33.73]
					b.4 - 7 days	3.55 [2.33; 4.77]
					c.> 7 days	2.15 [1.01; 3.28]
					2. extract type	
					a. methanol	19.07 [4.70; 33.43]
					b. ethanol	5.55 [3.59; 7.51]
					c.aqueous	1.62 [0.63; 2.61]
					3. *Laportea* species	
					a. *L.*aestuans	10.23 [3.34; 17.12]
					b..*L.*ovalifolia	1.61 [0.54; 2.68]
					4. study methods	
					a.Diclofenac induced rat	31.26 [22.46; 40.06]
					b.BPH induced rat	5.55 [3.59; 7.51]
5.Peroxidase	1.92 [1.29; 2.55]	<0.0001	61%; 0.001		no significant variables	
6.GR	4.09 [2.22; 5.95]	<0.0001	8.4%; 0.29		Low heterogeneity	
7.GST	4.08 [2.95; 5.21]	<0.0001	49.1%; 0.12		Low heterogeneity	
